# Trans-Ethnic Fine-Mapping of Lipid Loci Identifies Population-Specific Signals and Allelic Heterogeneity That Increases the Trait Variance Explained

**DOI:** 10.1371/journal.pgen.1003379

**Published:** 2013-03-21

**Authors:** Ying Wu, Lindsay L. Waite, Anne U. Jackson, Wayne H-H. Sheu, Steven Buyske, Devin Absher, Donna K. Arnett, Eric Boerwinkle, Lori L. Bonnycastle, Cara L. Carty, Iona Cheng, Barbara Cochran, Damien C. Croteau-Chonka, Logan Dumitrescu, Charles B. Eaton, Nora Franceschini, Xiuqing Guo, Brian E. Henderson, Lucia A. Hindorff, Eric Kim, Leena Kinnunen, Pirjo Komulainen, Wen-Jane Lee, Loic Le Marchand, Yi Lin, Jaana Lindström, Oddgeir Lingaas-Holmen, Sabrina L. Mitchell, Narisu Narisu, Jennifer G. Robinson, Fred Schumacher, Alena Stančáková, Jouko Sundvall, Yun-Ju Sung, Amy J. Swift, Wen-Chang Wang, Lynne Wilkens, Tom Wilsgaard, Alicia M. Young, Linda S. Adair, Christie M. Ballantyne, Petra Bůžková, Aravinda Chakravarti, Francis S. Collins, David Duggan, Alan B. Feranil, Low-Tone Ho, Yi-Jen Hung, Steven C. Hunt, Kristian Hveem, Jyh-Ming J. Juang, Antero Y. Kesäniemi, Johanna Kuusisto, Markku Laakso, Timo A. Lakka, I-Te Lee, Mark F. Leppert, Tara C. Matise, Leena Moilanen, Inger Njølstad, Ulrike Peters, Thomas Quertermous, Rainer Rauramaa, Jerome I. Rotter, Jouko Saramies, Jaakko Tuomilehto, Matti Uusitupa, Tzung-Dau Wang, Michael Boehnke, Christopher A. Haiman, Yii-Der I. Chen, Charles Kooperberg, Themistocles L. Assimes, Dana C. Crawford, Chao A. Hsiung, Kari E. North, Karen L. Mohlke

**Affiliations:** 1 Department of Genetics, University of North Carolina, Chapel Hill, North Carolina, United States of America; 2 HudsonAlpha Institute for Biotechnology, Huntsville, Alabama, United States of America; 3 Department of Biostatistics and Center for Statistical Genetics, University of Michigan, Ann Arbor, Michigan, United States of America; 4 Division of Endocrine and Metabolism, Department of Internal Medicine, Taichung Veterans General Hospital, Taichung, Taiwan; 5 School of Medicine, National Yang-Ming University, Taipei, Taiwan; 6 College of Medicine, National Defense Medical Center, Taipei, Taiwan; 7 Department of Statistics and Biostatistics, Rutgers University, Piscataway, New Jersey, United States of America; 8 Department of Epidemiology, University of Alabama at Birmingham, Birmingham, Alabama, United States of America; 9 The Human Genetics Center, University of Texas Health Science Center, Houston, Texas, United States of America; 10 Genome Technology Branch, National Human Genome Research Institute, National Institutes of Health, Bethesda, Maryland, United States of America; 11 Public Health Sciences, Fred Hutchinson Cancer Research Center, Seattle, Washington, United States of America; 12 University of Hawaii Cancer Center, Honolulu, Hawaii, United States of America; 13 Department of Molecular Physiology and Biophysics, Center for Human Genetics Research, Vanderbilt University, Nashville, Tennessee, United States of America; 14 Departments of Family Medicine and Epidemiology, Alpert Medical School, Brown University, Providence, Rhode Island, United States of America; 15 Department of Epidemiology, University of North Carolina, Chapel Hill, North Carolina, United States of America; 16 Medical Genetics Institute, Cedars-Sinai Medical Center, Los Angeles, California, United States of America; 17 Department of Preventive Medicine, Keck School of Medicine, University of Southern California, Los Angeles, California, United States of America; 18 Office of Population Genomics, National Human Genome Research Institute, National Institutes of Health, Bethesda, Maryland, United States of America; 19 Diabetes Prevention Unit, National Institute for Health and Welfare, Helsinki, Finland; 20 Kuopio Research Institute of Exercise Medicine, Kuopio, Finland; 21 Department of Medical Research, Taichung Veterans General Hospital, Taichung, Taiwan; 22 HUNT Research Centre, Department of Public Health and General Practice, Norwegian University of Science and Technology, Levanger, Norway; 23 University of Iowa, Iowa City, Iowa, United States of America; 24 Department of Medicine, University of Eastern Finland and Kuopio University Hospital, Kuopio, Finland; 25 National Institute for Health and Welfare, Disease Risk Unit, Helsinki, Finland; 26 Division of Biostatistics, Washington University School of Medicine, St. Louis, Missouri, United States of America; 27 Division of Biostatistics and Bioinformatics, Institute of Population Health Sciences, National Health Research Institutes, Zhunan, Taiwan; 28 Department of Community Medicine, Faculty of Health Sciences, University of Tromsø, Tromsø, Norway; 29 Department of Nutrition, University of North Carolina, Chapel Hill, North Carolina, United States of America; 30 Baylor College of Medicine, Houston, Texas, United States of America; 31 Department of Biostatistics, University of Washington, Seattle, Washington, United States of America; 32 Center for Complex Disease Genomics, McKusick-Nathans Institute of Genetic Medicine, Johns Hopkins University School of Medicine, Baltimore, Maryland, United States of America; 33 Translational Genomics Research Institute, Phoenix, Arizona, United States of America; 34 Office of Population Studies Foundation, University of San Carlos, Cebu, Philippines; 35 Department of Internal Medicine and Department of Medical Research and Education, Taipei Veterans General Hospital, Taipei, Taiwan; 36 Division of Endocrinology and Metabolism, Tri-Service General Hospital, National Defense Medical Center, Taipei, Taiwan; 37 Department of Internal Medicine, University of Utah, Salt Lake City, Utah, United States of America; 38 Cardiovascular Center and Division of Cardiology, Department of Internal Medicine, National Taiwan University Hospital and National Taiwan University College of Medicine, Taipei, Taiwan; 39 Institute of Clinical Medicine, Department of Medicine, University of Oulu and Clinical Research Center, Oulu University Hospital, Oulu, Finland; 40 Institute of Biomedicine/Physiology, University of Eastern Finland, Kuopio Campus, Kuopio, Finland; 41 Department of Human Genetics, University of Utah School of Medicine, Salt Lake City, Utah, United States of America; 42 Department of Genetics, Rutgers University, Piscataway, New Jersey, United States of America; 43 Department of Medicine, Kuopio University Hospital, Kuopio, Finland; 44 Pirkanmaa Hospital District, Tampere, Finland; 45 School of Public Health, University of Washington, Seattle, Washington, United States of America; 46 Department of Medicine, Stanford University School of Medicine, Stanford, California, United States of America; 47 Department of Clinical Physiology and Nuclear Medicine, Kuopio University Hospital, Kuopio, Finland; 48 South Karelia Central Hospital, Lappeenranta, Finland; 49 South Ostrobothnia Central Hospital, Seinäjoki, Finland; 50 Red RECAVA Grupo RD06/0014/0015, Hospital Universitario La Paz, Madrid, Spain; 51 Centre for Vascular Prevention, Danube-University Krems, Krems, Austria; 52 Institute of Public Health and Clinical Nutrition, University of Eastern Finland, Kuopio, Finland; 53 Research Unit, Kuopio University Hospital, Kuopio, Finland; 54 Carolina Center for Genome Sciences, University of North Carolina, Chapel Hill, North Carolina, United States of America; Georgia Institute of Technology, United States of America

## Abstract

Genome-wide association studies (GWAS) have identified ∼100 loci associated with blood lipid levels, but much of the trait heritability remains unexplained, and at most loci the identities of the trait-influencing variants remain unknown. We conducted a trans-ethnic fine-mapping study at 18, 22, and 18 GWAS loci on the Metabochip for their association with triglycerides (TG), high-density lipoprotein cholesterol (HDL-C), and low-density lipoprotein cholesterol (LDL-C), respectively, in individuals of African American (n = 6,832), East Asian (n = 9,449), and European (n = 10,829) ancestry. We aimed to identify the variants with strongest association at each locus, identify additional and population-specific signals, refine association signals, and assess the relative significance of previously described functional variants. Among the 58 loci, 33 exhibited evidence of association at *P*<1×10^−4^ in at least one ancestry group. Sequential conditional analyses revealed that ten, nine, and four loci in African Americans, Europeans, and East Asians, respectively, exhibited two or more signals. At these loci, accounting for all signals led to a 1.3- to 1.8-fold increase in the explained phenotypic variance compared to the strongest signals. Distinct signals across ancestry groups were identified at *PCSK9* and *APOA5*. Trans-ethnic analyses narrowed the signals to smaller sets of variants at *GCKR*, *PPP1R3B*, *ABO*, *LCAT*, and *ABCA1*. Of 27 variants reported previously to have functional effects, 74% exhibited the strongest association at the respective signal. In conclusion, trans-ethnic high-density genotyping and analysis confirm the presence of allelic heterogeneity, allow the identification of population-specific variants, and limit the number of candidate SNPs for functional studies.

## Author Summary

Lipid traits are heritable, but many of the DNA variants that influence lipid levels remain unknown. In a genomic region, more than one variant may affect gene expression or function, and the frequencies of these variants can differ across populations. Genotyping densely spaced variants in individuals with different ancestries may increase the chance of identifying variants that affect gene expression or function. We analyzed high-density genotyped variants for association with TG, HDL-C, and LDL-C in African Americans, East Asians, and Europeans. At several genomic regions, we provide evidence that two or more variants can influence lipid traits; across loci, these additional signals increase the proportion of trait variation that can be explained by genes. At some association signals shared across populations, combining data from individuals of different ancestries narrowed the set of likely functional variants. At *PCSK9* and *APOA5*, the data suggest that different variants influence trait levels in different populations. Variants previously reported to alter gene expression or function frequently exhibited the strongest association at those signals. The multiple signals and population-specific characteristics of the loci described here may be shared by genetic loci for other complex traits.

## Introduction

Genome-wide association studies (GWAS) have identified many common genetic variants associated with human diseases and complex traits (www.genome.gov/gwastudies), including ∼100 loci associated with triglycerides (TG), high-density lipoprotein cholesterol (HDL-C), low-density lipoprotein cholesterol (LDL-C), or total cholesterol [1]–[5]. A majority of the lead SNPs at these loci have shown small effect sizes, leaving much of the trait heritability unexplained. Some of this missing heritability may be due to the incomplete coverage of functional common or rare variants and the poor representation of appropriate proxies on commercial genotyping arrays [6], [7]. Other missing heritability may result from a failure to detect the full spectrum of causative variants present at GWAS-identified loci.

Fine-mapping of GWAS signals should increase the power to detect variants that influence trait variability. Genotyping of additional variants at GWAS loci can identify SNPs with stronger evidence of association than the reported GWAS index SNPs and may help detect or further localize the underlying causal variants [7], [8]. The Metabochip is a high-density custom genotyping array designed to replicate and fine-map known GWAS signals for metabolic and atherosclerotic/cardiovascular endpoints, and more extensively, to identify all signals around the index SNPs [9], [10]. The fine-mapping SNPs spanned a wide range of allele frequencies including rare (minor allele frequency (MAF)<0.005) and less common (0.005≤MAF<0.05) SNPs selected from the catalogs of the International HapMap Project and the August 2009 release of the 1000 Genomes Project. SNPs annotated as nonsynonymous, essential splice site or stop codon were included regardless of MAF, design score, or the presence of nearby SNPs [10]. The Metabochip contains densely spaced SNPs at 18, 22, and 18 loci previously reported for TG, HDL-C, and LDL-C, respectively.

Allelic heterogeneity, in which different variants at the same gene/locus affect the same phenotype, is a frequent characteristic of both single-gene and complex disorders. Recently GWAS have identified more than one independent signal at loci associated with coronary artery disease [11] and type 2 diabetes [12], [13]. Among a set of 30 lipid loci reported through GWAS, secondary SNPs that exhibited weak to moderate LD with the corresponding index SNPs and displayed little change of association in conditional analyses were detected at seven loci including *CETP*, *LIPC*, *APOA5*, *APOE*, *LDLR*, *ABCG8*, and *LPL* [4]. More than one association signal also was detected at 26 of 95 lipid loci reported by the Global Lipids Genetics Consortium [5]. However, allelic heterogeneity has not been comprehensively evaluated for common traits including lipid traits across ethnically diverse populations, especially in non-European populations such as African Americans and East Asians.

Due to divergent evolutionary and migratory histories, patterns of linkage disequilibrium (LD) vary across ancestry groups [14]. Greater haplotype diversity in some ancestry groups, especially in African ancestry populations, may facilitate the localization of functional variants that show association signals delimited in part due to weaker LD with neighboring SNPs [14], [15]. A recent multi-ethnic analysis of lipid associated loci demonstrated that genetic determinants at many lipid loci differed between European Americans and African Americans [16]. For example, in African Americans from the PAGE consortium [9], [17], a reported regulatory variant rs12740374 at *CELSR2/PSRC1/SORT1* locus [18] was more strongly associated with LDL-C compared to many nearby variants demonstrating similar strength of association in European ancestry individuals [5]. High-density genotyping enables trans-ethnic fine-mapping studies to narrow the set of plausible candidate functional variants at GWAS loci without introducing uncertainty through imputation [19].

In this study, we analyzed high-density genotyped SNPs on the Metabochip for their associations with TG, HDL-C, and LDL-C in 6,832 African Americans, 9,449 East Asians, and 10,829 Europeans at 58 known lipid loci. We sought to (i) identify the variants with the strongest evidence of association at each locus in populations with different ancestries and in the combined trans-ethnic samples; (ii) investigate allelic heterogeneity and population-specific signals at the established lipid loci; (iii) explore whether high-density genotyping in diverse ethnic populations would narrow the sets of plausible candidate functional variants for further study; and (iv) assess whether the variants reported to have functional effects on gene expression or protein function during the past 30 years of biological study exhibited the strongest evidence of association at the corresponding GWAS signals.

## Results

### Loci with evidence of association in diverse populations and in the combined trans-ethnic samples

Descriptions of the collection, phenotyping, and genotyping of study samples for each study site are provided in Table S1. Given that all 58 loci have *a priori* genome-wide significant evidence of association with one or more of these three lipid traits, we used a *P* value threshold of 1×10^−4^ as an approximate correction for the mean of 451 SNPs tested at each locus in African Americans (Table S2). An average of 273 SNPs per locus was tested in East Asians and an average of 291 in Europeans, but we applied the same, more conservative, *P* value threshold of 1×10^−4^ to these two groups as well.

A total of 33 loci (nine for TG, 14 for HDL-C, and 10 for LDL-C) exhibited evidence of association at *P*<1×10^−4^ in at least one of the three ancestry groups, including 22 loci in African Americans, 17 in East Asians, and 31 in Europeans (Table S3A–S3C). The variants that reached this threshold of significance were common (MAF≥0.05), except at three loci (*PCSK9* and *ABO* for LDL-C, and *APOA5* for HDL-C) in African Americans and two loci (*PCSK9* and *TOP1*, both for LDL-C) in European ancestry individuals. When individuals of diverse ancestry groups were combined, 11, 15, and 12 loci showed evidence of significant association with TG, HDL-C, and LDL-C, respectively (Table S4A–S4C). Among these 38 loci, six loci had not reached the *P* value threshold of 10^−4^ within any individual ancestry group, including *CETP* and *NAT* for TG, *GALNT2* and *MMAB* for HDL-C, and *TRIB1* and *TIMD4* for LDL-C. One locus, *COBLL1*, was significantly associated with HDL-C in Europeans alone (*P* = 8.5×10^−5^), but displayed less evidence of association in the combined trans-ethnic samples (*P* = 1.6×10^−4^).

### Loci with evidence of multiple signals at a locus, and often population-specific signals

To assess the presence of two or more signals at each locus that exhibited evidence of association in at least one ancestry group, we performed sequential conditional analyses by adding the most strongly associated SNP to the regression model as a covariate and testing the association with each of the remaining regional SNPs independently. A set of sequential conditional analyses were followed by inclusion of the strongest SNP in each conditional model until the most strongly associated SNP showed a conditional *P* value>10^−4^ and was not annotated as a nonsense or nonsynonymous substitution. We also investigated whether association signals were population-specific, which we defined as association signals with variants that are not variable in the samples from the other two ancestry groups in this study or in the 1000 Genomes Project populations that represent those groups among total European ancestry (EUR), total East Asian ancestry (ASN), or total west African ancestry (AFR).

In African Americans, sequential conditional analyses revealed that 10 of the 22 loci with evidence of association exhibited two or more signals at *P*<10^−4^ (Table 1). Two loci (*PCSK9* and the *TOMM40*-*APOE-APOC4* cluster; both for LDL-C) each had seven signals, four loci (*APOB* for LDL-C, *LDLR* for LDL-C, *LCAT* for HDL-C, and *CETP* for HDL-C) had three signals, and another four loci (*APOB*, *APOC1*, *APOA5*, and *LPL*; all for TG) had two signals. Among the 10 loci with two or more signals, all these signals led to an average 1.8-fold increase in the amount of phenotypic variance (R^2^) compared to that explained by the strongest signals alone (See Method) in African Americans. Among these 34 signals, 15 were represented by less common (0.005≤MAF<0.05, n = 11) or rare (MAF<0.005, n = 4) variants. In addition, 15 signals at eight loci were African American-specific. If we only include SNPs that meet a locus-specific *P*-value threshold based on the number of genotyped SNPs (Table S2), *LPL* for TG and *APOB* for both TG and LDL each had one signal, and the seven loci with multiple signals still showed an average of 1.8-fold increase in the explained phenotypic variance.

**Table 1:** Lipid loci with multiple and population-specific signals in African Americans.

SNP	Annotation	Effect/non-effect allele	African American (n = 6,832)						Variance explained by the strongest signal^d^	Variance explained by all signals^d^	East Asian (n = 9,449)			European (n = 10,829)		
			EAF	LD (*r* ^2^/D′)^a^	β^b^	*P*_initial_	β^b^	*P*_conditional_ c			EAF	β^b^	*P* e	EAF	β^b^	*P* e
*PCSK9* f for LDL-C																
rs28362286	*PCSK9*-C679X	A/C	0.009	----	−0.956	4.8E-17	----	----	1.3%	3.6%	0	----	----	0	----	----
rs28362263	*PCSK9*-A443T	A/G	0.097	0.00/1.00	−0.206	3.1E-09	−0.218	2.7E-10			0	----	----	0	----	----
rs28362261	*PCSK9*-N425S	A/G	0.985	0.00/1.00	0.361	2.1E-05	0.396	3.2E-06			0	----	----	0	----	----
rs67608943	*PCSK9*-Y142X	C/G	0.996	0.00/0.00	0.925	1.0E-07	0.798	4.2E-06			0	----	----	0	----	----
rs72646508	*PCSK9*-L253F	T/C	0.003	0.00/1.00	−0.720	9.2E-05	−0.773	2.9E-05			0	----	----	0	----	----
rs11800243	*PCSK9*-intron	A/G	0.044	0.00/1.00	−0.152	2.4E-03	−0.198	7.7E-05			0.030	−0.070	0.091	0.025	−0.006	0.89
rs11591147	*PCSK9*-R46L	T/G	0.003	0.00/1.00	−0.595	2.3E-03	−0.678	5.3E-04			0	----	----	0.040	−0.384	2.8E-30
*TOMM40-APOE-APOC4* for LDL-C																
rs7412	*APOE*-R176C	T/C	0.110	----	−0.536	6.7E-75	----	----	4.1%	6.6%	0.086	−0.411	1.1E-64	0.056	−0.505	5.4E-76
rs115299243	*APOE*-intron	A/G	0.980	0.00/0.99	0.391	3.4E-09	0.436	1.6E-11			0	----	----	0	----	----
rs1038026	*TOMM40*-intron	A/G	0.351	0.03/0.70	0.183	7.5E-21	0.102	2.6E-07			0.664	0.118	3.0E-16	0.508	−0.019	0.13
rs157588	*TOMM40*-intron	T/C	0.824	0.01/0.43	−0.009	0.72	0.153	2.0E-07			0.339	−0.119	1.1E-16	0.518	0.024	0.056
rs769449	*APOE*-intron	A/G	0.024	0.00/0.86	0.302	1.1E-06	0.295	4.1E-06			0.086	0.173	2.8E-12	0.160	0.121	1.7E-12
rs73939904	*APOC4*-upstream	A/C	0.940	0.00/0.16	0.143	2.5E-04	0.158	3.8E-05			0.986	0.135	0.076	0	----	----
rs8106922	*TOMM40*-intron	A/G	0.756	0.04/0.97	−0.110	4.1E-07	−0.097	9.9E-05			0.784	−0.027	0.10	0.531	−0.058	4.9E-06
*LDLR* for LDL-C																
rs73015011	----	T/C	0.820	----	0.194	5.7E-16	----	----	1.0%	1.8%	0.987	0.140	0.020	0.896	0.202	1.5E-22
rs114197570	*LDLR*-upstream	T/C	0.010	0.05/1.00	−0.689	3.0E-13	−0.549	1.2E-08			0	----	----	0	----	----
rs113190300	*LDLR*-upstream	T/C	0.048	0.22/1.00	0.046	0.29	0.241	1.4E-06			0	----	----	0	----	----
*LCAT* for HDL-C																
rs255054	*DPEP3*-upstream	A/G	0.789	----	−0.044	3.2E-07	----	----	0.3%	0.5%	0.904	−0.028	1.7E-03	0.819	−0.023	4.9E-04
rs114763908	*NFATC3*-intron	A/G	0.016	0.00/1.00	0.109	1.1E-04	0.118	2.5E-05			0	----	----	0	----	----
rs2230093	*NFATC3*-L100S	T/C	0.990	0.00/1.00	0.129	2.5E-04	0.120	6.3E-04			0	----	----	0	----	----
*APOB* for LDL-C																
rs568938	----	T/C	0.426	----	0.109	1.1E-08	----	----	0.6%	1.0%	0.966	0.000	0.99	0.771	0.078	1.9E-07
rs73920524	----	A/G	0.947	0.04/1.00	0.205	9.4E-07	0.167	8.8E-05			0.999	0.549	0.093	0	----	----
rs72653060	*APOB*-F299V	A/C	0.998	0.00/1.00	−1.128	2.6E-04	−1.151	1.8E-04			0	----	----	0	----	----
*CETP* for HDL-C																
rs247617	*CETP*-upstream	T/C	0.259	----	0.110	1.1E-42	----	----	2.6%	4.8%	0.166	0.071	3.0E-26	0.284	0.090	1.3E-58
rs5883	*CETP*-F287F	T/C	0.101	0.02/0.69	0.088	1.3E-13	0.109	1.8E-20			0.010	0.017	0.70	0.046	0.055	5.0E-06
rs17231520	*CETP*-5′UTR	A/G	0.069	0.21/0.99	0.175	2.2E-37	0.113	1.1E-13			0	----	----	0.002	−0.022	0.85
*APOC1-APOE* for TG																
rs12721054	*APOC1*-3′UTR	A/G	0.881	----	0.113	3.6E-19	----	----	1.0%	1.6%	0	----	----	0	----	----
rs769455	*APOE*-R163C	T/C	0.020	0.00/0.73	0.185	3.4E-10	0.174	2.6E-09			0	----	----	0	----	----
*APOB* for TG																
rs676210	*APOB*-P2739L	A/G	0.157	----	−0.052	4.0E-06	----	----	0.3%	0.6%	0.722	0.002	0.79	0.260	−0.047	2.1E-10
rs6752026	*APOB*-P145S	A/G	0.121	0.02/0.98	−0.033	8.8E-03	−0.043	8.9E-04			0	----	----	0	----	----
*APOA5* for TG																
rs3135506	*APOA5*-S19W	C/G	0.058	----	0.136	8.4E-15	----	----	0.9%	1.5%	0.005	0.136	0.17	0.058	0.121	3.3E-18
rs79624460	*BUD13*-intron	T/C	0.083	0.00/0.88	−0.102	4.8E-12	−0.095	1.4E-10			0	----	----	0	----	----
*LPL* for TG																
rs75551077	----	C/G	0.135	----	−0.072	1.3E-09	----	----	0.5%	0.6%	0.093	−0.087	6.4E-11	0.088	−0.075	3.6E-11
rs71778131	*LPL*-3′UTR	A/G	0.049	0.01/1.00	0.086	7.7E-06	0.077	7.3E-05			0	----	----	0.019	0.069	4.3E-03

a LD (r^2^/D′) with SNP showing the strongest evidence of association at each locus.

b β: effect size from an additive model and corresponding to the effect allele, in the unit of mmol/L for HDL-C, LDL-C and natural log transformed TG.

c *P* values of sequential conditional analyses, in which we added the SNP with the strongest evidence of association into the regression model as a covariate and tested for the next strongest SNP until the strongest SNP showed a conditional *P* value>10^−4^ and had no annotation suggesting potential function.

d Variance explained was estimated based on PAGE samples (n = 5,593).

e *P* values of initial association in East Asians and Europeans.

f Conditional analyses at LDL-C locus *PCSK9* were restricted to 5,593 PAGE samples because SNPs rs67608943 (Y142X), rs72646508 (L253F) and rs11591147 (R46L) were not polymorphic in HyperGEN samples.

**Figure 1:**
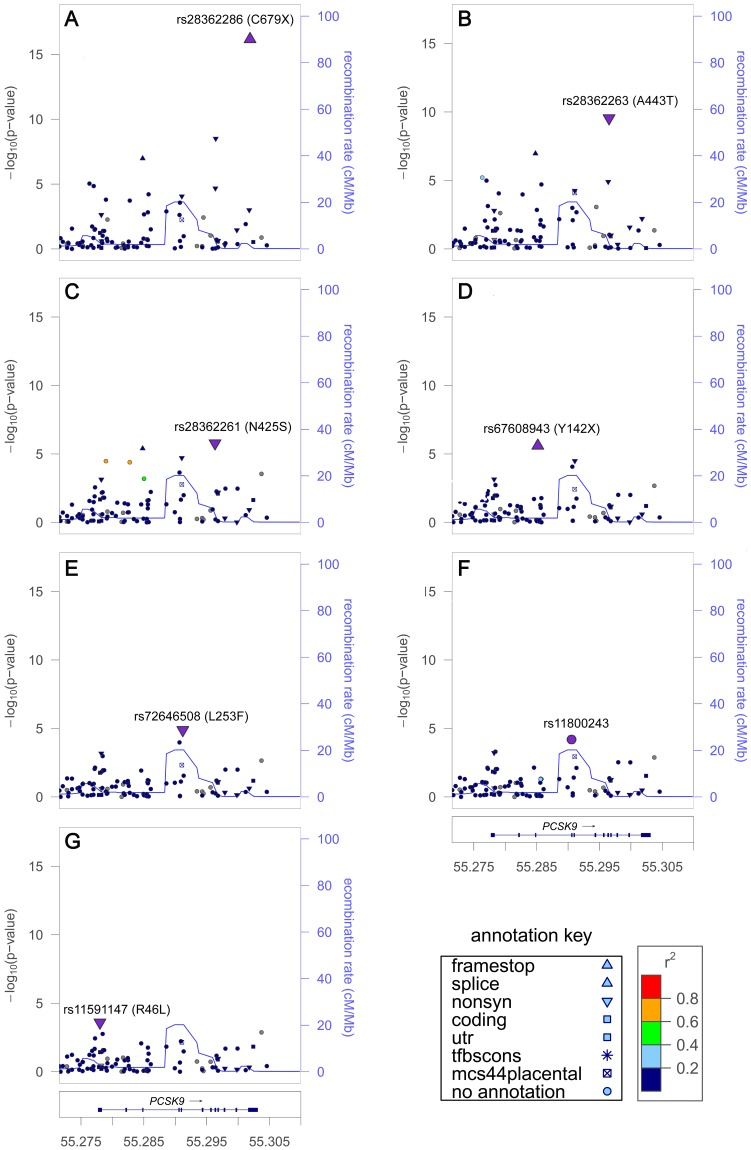
LDL-C locus *PCSK9* exhibited seven signals in African Americans. Initial association in the main analysis (A). Residual association in sequential conditional analysis by sequentially adding the lead SNPs into the regression model (B–G). Each SNP was colored according to its LD (*r^2^*) in the PAGE consortium, with the strongest SNP colored in purple and symbols designating genomic annotation defined in the ‘annotation key’. Genomic coordinates refer to build 36 (hg18).

The seven signals at *PCSK9* in African Americans included six nonsense or nonsynonymous variants previously shown to associate with LDL-C levels and to affect PCSK9 expression or function [20]–[22], along with an unreported intronic variant (Table 1). The strongest signals were a nonsense variant rs28362286 (C679X, Figure 1A) and a nonsynonymous variant rs28362263 (A443T, Figure 1B), which showed no reduction of association evidence when conditioned on C679X. Conditional analysis on both C679X and A443T yielded a third signal at rs28362261 (N425S, Figure 1C); and further conditional analyses successively implicated rs67608943 (Y142X, Figure 1D), rs72646508 (L253F, Figure 1E), and an intronic variant rs11800243 (Figure 1F). The seventh signal, which did not reach the *P_conditional_*<10^−4^ threshold, was represented by the nonsynonymous variant rs11591147 (R46L, Figure 1G) that exhibited the strongest and directionally consistent evidence of association with LDL-C in Europeans (*P_initial_* = 2.8×10^−30^, Table 2). The seven signals were weakly correlated with each other in African American individuals, and all pairwise LD *r^2^* values were less than 0.02. Among the seven *PCSK9* signals, the top five were African American-specific, and six were either less common or rare in African Americans. The lead SNP C679X accounted for 1.3% of the explained LDL-C phenotypic variance and the seven signals together explained 3.6% of the phenotypic variance in African Americans. *PCSK9* exhibited two signals in Europeans (R46L and rs2495477, Table 2), but no SNP reached *P_initial_*<10^−4^ in East Asians.

**Table 2:** Lipid loci with multiple signals in Europeans.

SNP	Annotation	Effect/non-effect allele	European (n = 10,829)						Variance explained by the strongest signal^d^	Variance explained by all signals^d^	African American (n = 6,832)			East Asian (n = 9,449)		
			EAF	LD (*r* ^2^/D′)^a^	β^b^	*P*_initial_	β^b^	*P*_conditional_ c			EAF	β^b^	*P* e	EAF	β^b^	*P* e
*APOA5* for TG																
rs3741298	*ZNF259*-intron	T/C	0.778	----	−0.108	9.7E-44	----	----	1.8%	2.4%	0.673	−0.034	9.8E-05	0.643	−0.073	1.2E-20
rs75919952	----	T/C	0.041	0.03/0.33	−0.039	0.018	−0.128	7.8E-14			0.012	0.030	0.45	0.042	−0.023	0.24
rs2075290	*ZNF259*-intron	T/C	0.918	0.40/1.00	−0.151	4.4E-37	−0.058	9.3E-05			0.943	−0.040	0.023	0.771	−0.093	2.1E-25
*TOMM40-APOE-APOC4* for LDL-C																
rs7412	*APOE*-R176C	T/C	0.056	----	−0.505	5.4E-76	----	----	3.4%	4.0%	0.110	−0.536	6.7E-75	0.086	−0.411	1.1E-64
rs56131196	*APOC1*-3′UTR	A/G	0.262	0.02/1.00	0.126	1.9E-18	0.092	1.5E-10			0.193	0.030	0.26	0.106	0.125	2.7E-08
rs35136575	----	C/G	0.739	0.01/1.00	0.056	9.6E-05	0.075	2.6E-07			0.817	0.014	0.55	0.904	−0.003	0.91
*CETP* for HDL-C																
rs56156922	----	T/C	0.716	----	−0.090	4.7E-59	----	----	2.3%	3.6%	0.854	−0.071	2.0E-12	0.830	−0.071	1.1E-26
rs12720922	*CETP*-intron	A/G	0.177	0.05/0.68	−0.098	6.7E-50	−0.073	2.2E-26			0.332	−0.017	0.026	0.137	−0.058	6.9E-16
rs5883	*CETP*-F287F	T/C	0.046	0.03/1.00	0.055	5.0E-06	0.065	6.2E-08			0.101	0.088	1.3E-13	0.010	0.017	0.70
*PCSK9* for LDL-C																
rs11591147	*PCSK9*-R46L	T/G	0.040	----	−0.384	2.8E-30	----	----	1.2%	1.3%	0.003	−0.595	2.3E-03	----	----	----
rs2495477	*PCSK9*-intron	A/G	0.571	0.00/0.21	0.086	1.3E-11	0.057	9.4E-06			0.292	0.055	7.4E-03	0.761	0.048	2.6E-03
*GCKR* for TG																
rs1260326	*GCKR*-P446L	T/C	0.350	----	0.069	4.4E-24	----	----	0.9%	1.0%	0.149	0.065	2.2E-08	0.484	0.056	1.5E-13
rs13399758	*CAD*-intron	T/C	0.949	0.04/1.00	0.083	2.7E-08	0.060	7.6E-05			0.483	0.023	5.4E-03	0.999	0.191	0.21
*LIPC* for HDL-C																
rs10468017	----	T/C	0.327	----	0.051	2.5E-21	----	----	0.8%	1.4%	0.160	0.020	0.045	0.183	0.031	1.3E-06
rs1077834	*LIPC*-5′UTR	T/C	0.751	0.02/0.13	−0.050	1.9E-17	−0.047	3.0E-15			0.481	−0.034	2.2E-06	0.598	−0.037	1.8E-13
*APOB* for LDL-C																
rs934198	----	T/G	0.298	----	0.116	3.7E-17	----	----	0.7%	0.8%	0.138	0.057	0.037	0.138	0.058	3.3E-03
rs668948	----	A/G	0.810	0.13/1.00	0.102	2.5E-10	0.068	5.1E-05			0.446	0.100	1.2E-07	0.966	−0.001	0.99
*LPL* for TG																
rs15285	3′UTR	T/C	0.258	----	−0.061	1.1E-16	----	----	0.6%	0.8%	0.505	−0.042	2.1E-07	0.187	−0.055	2.0E-08
rs34770253	----	T/C	0.802	0.39/1.00	0.014	0.098	−0.044	1.5E-05			0.860	−0.018	0.12	0.905	0.008	0.55
*LPL* for HDL-C																
rs15285	3′UTR	T/C	0.258	----	0.035	1.4E-09	----	----	0.3%	0.4%	0.505	0.047	3.5E-11	0.182	0.022	6.8E-04
rs4407894	----	T/C	0.360	0.24/1.00	−0.031	4.3E-09	−0.023	7.3E-05			0.167	−0.014	0.13	0.637	−0.018	3.7E-04

a LD (*r* ^2^/D′) with SNP showing the strongest evidence of association at each locus.

b β: effect size from an additive model and corresponding to the effect allele, in the unit of mmol/L for HDL-C, LDL-C and natural log transformed TG.

c *P* values of sequential conditional analyses, in which we added the SNP with the strongest evidence of association into the regression model as a covariate and tested for the next strongest SNP until the strongest SNP showed a conditional *P* value>10^−4^ and had no annotation suggesting potential function.

d Variance explained by SNPs at each locus was estimated based on European samples.

e *P* values of initial association in African Americans and East Asians.

At the *TOMM40-APOE-APOC4* cluster, the seven signals in African Americans explained 6.6% of the LDL-C phenotypic variance compared to 4.1% explained by the strongest signal R176C, which had reported functional effects [23] (Table 1, Figure S1). These seven signals were not entirely independent of one another. The fourth signal, rs157588, showed association with LDL-C (*P* = 2.0×10^−7^) only after conditioning on the top three signals, but not in the original unconditioned association analysis (*P* = 0.72). The trait-decreasing allele (G allele: freq = 0.176) of rs157588 was present on haplotypes containing the trait-increasing allele of the third signal rs1038026 (A allele: freq = 0.351), thus the association of the fourth signal increased in significance after accounting for linkage disequilibrium (*r^2^/D′* = 0.35/0.92) with the third signal at the same locus. Haplotype analysis revealed that compared to the reference A-A (increasing-increasing) haplotype, the G-G (decreasing-decreasing) haplotype only displayed modest association with LDL-C (*P* = 7.5×10^−3^), but the A–G (rs1038026 increasing- rs157588 decreasing) haplotype showed significant association with decreased level of LDL-C (*P* = 1.5×10^−10^) (Table S5). In Europeans (Table 2) and East Asians (Table 3), three and two signals were identified at *TOMM40*-*APOE-APOC4*, respectively. The known functional variant R176C exhibited the strongest evidence of association across the three ancestry groups, with effect sizes of −0.536, −0.505, and −0.411 mmol/L in individuals of African American, European, and East Asian ancestry, respectively (Table 1). However, another *APOE* variant rs429358 (C130R), that together with R176C, defines the three major isoforms of *APOE* (ε2, ε3, and ε4) [7], [24], was not successfully genotyped, therefore the LDL-C association with either C130R or the *APOE* haplotype was unavailable in this study.

**Table 3:** Lipid loci with multiple signals in East Asians.

SNP	Annotation	Effect/non-effect allele	East Asian (n = 9,449)						Variance explained by the strongest signal^d^	Variance explained by all signals^d^	African American (n = 6,832)			European (n = 10,829)		
			EAF	LD (*r* ^2^/D′)^a^	β^b^	*P*_initial_	β^b^	*P*_conditional_ c			EAF	β^b^	*P* e	EAF	β^b^	*P* e
*APOA5* for TG																
rs651821	*APOA5*: -3A>G	T/C	0.725	----	−0.145	7.2E-68	----	----	2.6%	3.4%	0.851	−0.037	1.4E-03	0.921	−0.151	8.5E-36
rs2075291	*APOA5*-G185C	A/C	0.064	0.09/1.00	0.201	3.7E-37	0.106	7.2E-10			0.002	0.204	0.028	0.0003	0.269	0.23
rs11604424	*ZNF259*-intron	T/C	0.650	0.39/1.00	−0.075	2.0E-21	−0.045	4.8E-05			0.725	−0.020	0.032	0.765	−0.101	6.5E-40
*TOMM40-APOE-APOC4* for LDL-C																
rs7412	*APOE*-R176C	T/C	0.086	----	−0.411	1.1E-64	----	----	8.0%	9.0%	0.110	−0.536	6.7E-75	0.056	−0.505	5.4E-76
rs769449	*APOE*-intron	A/G	0.086	0.00/1.00	0.173	2.8E-12	0.191	3.8E-06			0.024	0.302	1.1E-06	0.160	0.121	1.7E-12
*CETP* for HDL-C																
rs17231506	*CETP*-5′UTR	T/C	0.168	----	0.073	3.6E-28	----	----	1.0%	2.3%	0.146	0.071	2.9E-12	0.284	0.090	2.2E-58
rs7499892	*CETP*-intron	T/C	0.164	0.00/1.00	−0.052	2.8E-15	−0.065	2.0E-07			0.372	−0.066	1.4E-16	0.173	−0.097	4.6E-48
*ABO* for LDL-C																
rs9411476	*ABO*-downstream	A/G	0.162	----	0.106	1.1E-08	----	----	0.8%	1.8%	0.121	0.043	0.14	0.005	−0.190	0.037
rs191637055	*ADAMTSL*-intron	A/C	0.998	0.00/1.00	−0.688	1.1E-03	−1.055	4.0E-05			0.977	−0.014	0.83	0	----	----

a LD (*r* ^2^/D′) with SNP showing the strongest evidence of association at each locus.

b β: effect size from an additive model and corresponding to the effect allele, in the unit of mmol/L for HDL-C, LDL-C and natural log transformed TG.

c *P* values of sequential conditional analyses, in which we added the SNP with the strongest evidence of association into the regression model as a covariate and tested for the next strongest SNP until the strongest SNP showed a conditional *P* value>10^−4^ and had no annotation suggesting potential function.

d Variance explained by SNPs at each locus was estimated based on CLHNS samples (n = 1,716).

e *P* values of initial association in African Americans and Europeans.

In Europeans, 21 signals at nine of the 31 loci exhibited multiple signals for at least one of the three lipid traits at *P*<10^−4^ (Table 2). Three loci (*APOA5* for TG, *TOMM40*-*APOE-APOC4* cluster for LDL-C, and *CETP* for HDL-C) each had three signals while another six loci (*PCSK9* for LDL-C, *GCKR* for TG, *LIPC* for HDL-C, *APOB* for LDL-C, and *LPL* for both TG and HDL-C) each had two signals. At the nine loci that had two or more signals, all association signals resulted in an average of 1.3-fold increase in the explained phenotypic variance compared to the strongest signals alone across loci. At *PCSK9*, rs11591147 (R46L) exhibited the strongest evidence of association in Europeans. As reported above, R46L also represented the seventh signal in African Americans. R46L accounted for 1.2% of the total variation in LDL-C levels in Europeans compared the 0.16% in African Americans. This SNP was not variable in the 1000 Genomes Project ASN samples (East Asian ancestry) and the >9,000 East Asian individuals in this study.

In East Asians, we observed three signals at the TG locus *APOA5*, and two signals at three loci including *TOMM40*-*APOE-APOC4* cluster for LDL-C, *CETP* for HDL-C, and *ABO* for LDL-C (Table 3). At the four loci that exhibited multiple signals, all the association signals increased the explained phenotypic variance by an average of 1.3-fold compared to the strongest signal across loci. The second signal at *APOA5* was the nonsynonymous variant G185C previously reported to affect the protein function [25]. Although G185C was not unique to East Asians, the frequency was very low in African Americans (MAF = 0.002, *P* = 0.028) and Europeans (MAF = 0.0003, *P* = 0.23), and the low allele frequency meant that this study had less than 5% statistical power to detect the association in these groups.

At *APOA5*, which exhibited multiple signals in all three populations (Table 1, Table 2, Table 3), the strongest TG-associated SNPs differed and were not in high LD (*r^2^*<0.8) with each other in any of the ancestry groups. In African Americans, the two signals S19W (MAF = 0.058, *P* = 8.4×10^−15^) and rs79624460 (MAF = 0.083, *P* = 4.8×10^−12^), showed no evidence of significant association in East Asians (Table 1), likely due to the low allele frequency and the limited power (∼10%) to detect the association. The three signals at *APOA5* in East Asians were only modestly associated with TG in African Americans (all *P*>10^−3^, Table 3). The SNP LD *r^2^* values between the African American and East Asian signals were less than 0.02 in both populations, suggesting that they represent distinct *APOA5* signals in the two ancestry groups. In addition, the *APOA5* signal rs3741298 (*P* = 9.7×10^−44^, MAF = 0.222) in Europeans exhibited evidence of association with TG in African Americans (*P* = 9.8×10^−5^, MAF = 0.327) and East Asians (*P* = 1.2×10^−20^, MAF = 0.357), but the significance levels of the association with rs3741298 were substantially attenuated by conditioning on the strongest signals S19W in African Americans (*P* = 0.10) and rs651821 in East Asians (*P* = 0.88). In Europeans, the associations with rs3741298 were partially removed when conditioning on S19W and rs651821 (*P_conditional_* = 1.7×10^−28^ and 3.1×10^−17^, respectively). The European signal rs3741298 was moderately correlated with the African American signal S19W (LD *r^2^* = 0.21 and 0.10 in the 1000 Genomes Project EUR samples (European ancestry) and in PAGE African American samples, respectively), and with the East Asian signal rs651821 (LD *r^2^* = 0.31 and 0.28 in 1000 Genomes Project EUR and ASN samples, respectively). Notably, the effect sizes of the two reported functional variants S19W [26] and G185C [25] at *APOA5* were similar across the three groups (S19W, African American: 0.136; East Asian: 0.136; European: 0.121 and G185C, African American: 0.204; East Asian: 0.201; European: 0.269 mmol/L in log_e_ scale) despite the limited power to detect significant evidence of association at low allele frequencies. These findings support the hypothesis that causative variants may have a similar genetic impact on trait variation across populations if not influenced by hidden gene-gene or gene-environment interactions [27]. We also observed that the second European signal rs75919952 exhibited nominal evidence of association (*P*_initial_ = 0.018, MAF = 0.041), but was not associated with TG in the other two groups (Table 2). The lack of association may be due to insufficient power (15% and 55% in African Americans and East Asians, respectively; assuming α = 0.05) corresponding to the lower allele frequency (MAF = 0.012) in African Americans, the smaller sample sizes in both populations, or underlying interactions.

### Trans-ethnic high-density genotyping narrowed the region of association signals

**Figure 2:**
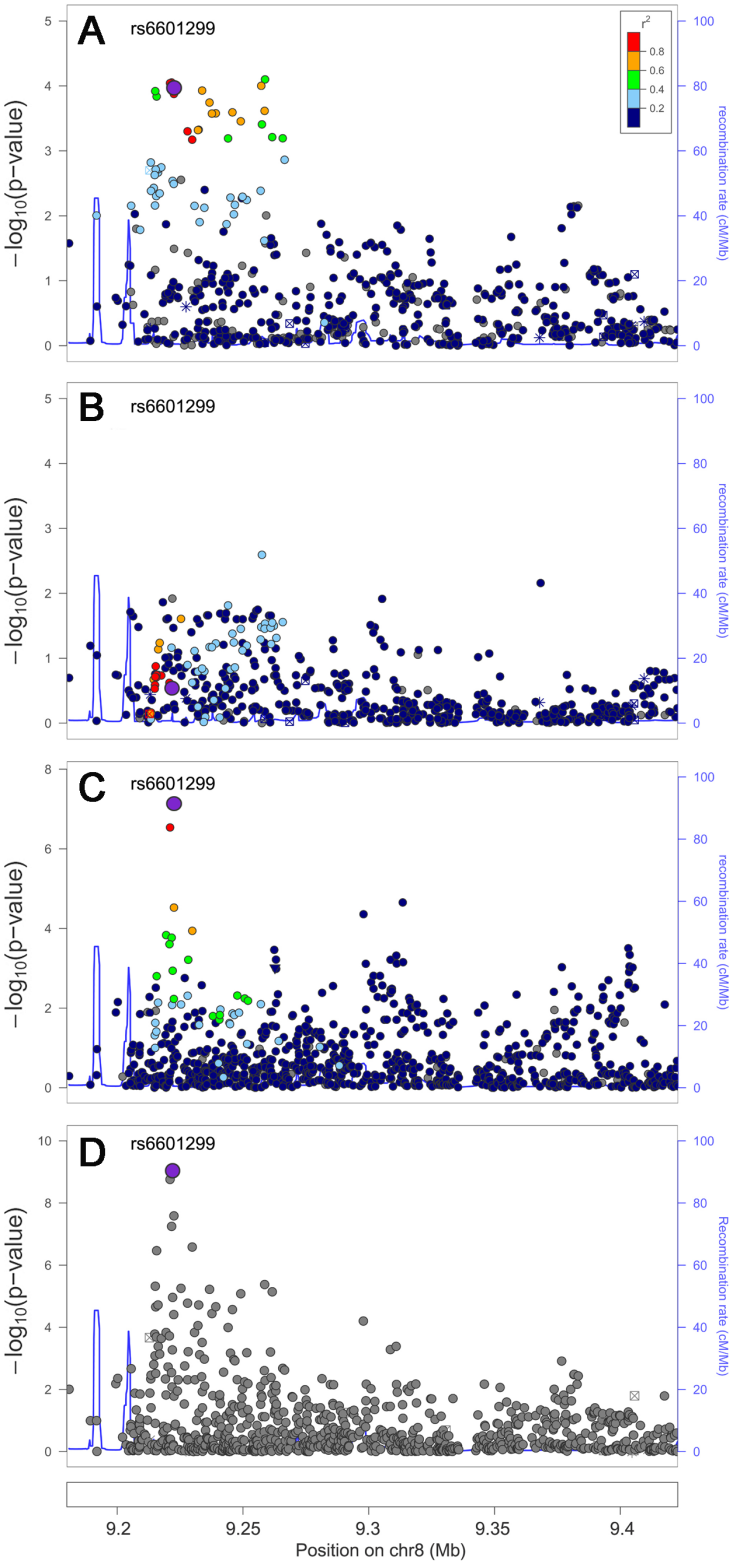
Trans-ethnic high-density genotyping narrows the association signal at the HDL-C locus *PPP1R3B*. Association in Europeans (A), East Asians (B), African Americans (C) and in a combined trans-ethnic meta-analysis (D). Index SNP rs6601299 colored in purple is the variant showing strongest evidence of association in the combined trans-ethnic meta-analysis.

We next examined whether trans-ethnic meta-analysis or comparison across ancestries would refine the association signals by narrowing the genomic regions where functional variants might be expected to reside. The trans-ethnic analysis allowed the refinement of association signals at loci of *GCKR*, *PPP1R3B*, *ABO*, *LCAT*, and *ABCA1* (Table 4, Table S3A–S3C). The signal at *GCKR* was localized to the reported functional variant P446L [28] due to the limited LD in African Americans (Figure S2A–S2D). Notably, there were seven and six variants in high LD (*r^2^*>0.8) with P446L in the 1000 Genomes Project ASN and EUR samples, but no SNP with LD *r^2^*>0.8 in African American individuals. At the signal ∼200 kb from the *PPP1R3B* gene for which no functional regulatory variant(s) have been reported, the association signal was narrowed from 4 SNPs spanning 36 kb (*P*<10^−4^) in Europeans to two highly correlated SNPs located 1 kb apart in African Americans (rs6601299, *P* = 8.0×10^−8^ and rs4841132, *P* = 2.9×10^−7^; LD *r^2^*>0.94) (Figure 2). The lead SNP rs6601299 was in high LD with 11 variants in the 1000 Genomes Project EUR samples but only highly correlated with two and one variant in the 1000 Genomes Project AFR samples (West African ancestry) and PAGE African American individuals, respectively. At the *ABO* locus, trans-ethnic meta-analysis revealed six SNPs exhibiting stronger evidence of association (*P*<1.1×10^−11^) with LDL-C compared to other variants in the same region (*P*>2.3×10^−7^) (Figure S3A–S3D). At the locus *LCAT* for HDL-C, the association signals spanned ∼800 kb, ∼360 kb, and ∼360 kb in Europeans, East Asians, and African Americans, with a ∼50 kb overlapping region. Trans-ethnic meta-analysis of all samples localized the signal to four variants spanning this 50 kb region (Figure S4A–S4D). At HDL-C locus *ABCA1*, the reported GWAS index SNP rs1883025 consistently showed the strongest association within each of the three ancestry groups that we examined, but the significance level of the association was similar to those of the nearby SNPs. Trans-ethnic meta-analysis refined the signal by revealing that rs1883025 (*P* = 4.3×10^−17^) and rs2575876 (*P* = 1.8×10^−15^) displayed much stronger association than the neighboring SNPs (*P*>8.4×10^−10^) (Figure S5A–S5D).

**Table 4:** Trans-ethnic fine-mapping narrowed the association signals.

Locus/Trait	SNP	Meta-analysis				African American			East Asian			European		
		*P* meta^a^	Direction^b^	*P* het^c^	*I* ^2^ d	MAF	β	*P*	MAF	β	*P*	MAF	β	*P*
*GCKR*/TG	rs1260326 (P446L)	1.6E-42	+++++++++++	0.72	0	0.149	0.07	2.2E-08	0.484	0.06	1.5E-13	0.350	0.07	4.4E-24
*PPP1R3B*/HDL	rs6601299	8.8E-10	−−−−−−−−−+−	0.16	30.6	0.121	−0.06	8.0E-08	0.052	−0.02	0.29	0.160	−0.03	1.1E-04
*ABO*/LDL	rs2519093	2.2E-13	+++++++++++	0.91	0	0.105	0.10	6.6E-04	0.187	0.09	5.4E-07	0.196	0.07	1.7E-05
*LCAT/*HDL	rs3785100 (*SLC12A4*-E4G)	9.0E-12	−−−−−+−−−−−	0.55	0	0.217	−0.04	6.5E-07	0.096	−0.03	1.7E-03	0.155	−0.03	1.6E-04
*ABCA1/*HDL	rs1883025	4.3E-17	−+−−−−−−−−−	0.63	0	0.336	−0.02	0.018	0.271	−0.04	3.7E-11	0.209	−0.03	4.5E-07

a *P* meta: *P* values from meta-analysis combining samples of African American, East Asian and European ancestries.

b Direction: effect direction of each individual studies in the order of ARIC, MEC, WHI batch1, WHI batch2, HyperGEN, CLHNS, TAICHI, Finnish T2D, Finnish unaffected, Norwegian T2D and Norwegian unaffected.

c *P* het: *P* values for heterogeneity, indicating whether observed effect sizes are homogeneous across ancestry samples.

d *I* ^2^: index of the degree of heterogeneity.

### Reported functional variants were frequently the most strongly associated ones at a signal

Among loci associated with at least one lipid trait (*P*<10^−4^), at least 27 variants at 15 loci have been previously reported [18], [22], [23], [25], [26], [28]–[47] to functionally influence gene expression or protein function *in vitro* (Table 5). Among the 27 variants, 17 are present on the Metabochip and two are well-represented by perfect proxies in complete LD (*r^2^* = 1) based on the 1000 Genomes Project EUR data. Of the 19 reported functional variants, 14 (74%) exhibited the strongest association *P*-value among all SNPs at that signal in at least one population. In addition, two more reported functional variants (*APOB*-rs7575840, *P* = 7.0×10^−17^ and *LPL*-rs328, *P* = 2.3×10^−11^) were in high LD (*r^2^*>0.95) with the most strongly associated variants and showed similar evidence of association (*APOB*-rs934198, *P* = 3.7×10^−17^; *LPL*-rs1803924, *P* = 1.1×10^−11^). If we include these two variants, then 16 of the 19 (84%) reported functional variants displayed the strongest association *P*-value at the primary, secondary, or successive signals. The remaining three reported functional variants: *LDLR*-rs688 (N591N), *LPL*-rs1801177 (D9N), and *HMGCR*-rs3761740 (911C>A), were poorly tagged (LD *r^2^*<0.2) by the strongest variants in our data. Additional functional variants may exist at these loci that have not yet been reported to change gene expression/protein function or that were not identified in our literature search. For example, P2739L and P145S that represented the two signals at *APOB* (Table 1) were predicted by PolyPhen [48] to be ‘probably damaging’ with a score of ‘1’, although their functional roles were unclear.

**Table 5:** Reported functional variants exhibited the strongest association at a signal (*P*<10^−4^).

Reported functional variants [ref]	Reported functional variants on Metabochip	Variants with strongest association at a signal	Signal	Ethnic group*	MAF	Notes
*PCSK9*: rs28362286 (C679X) [22]	Yes	rs28362286	1st	AA	0.009	Same variant
*PCSK9*: rs28362263 (A443T) [29]	Yes	rs28362263	2nd	AA	0.097	Same variant
*PCSK9*: rs28362261 (N425S) [30]	Yes	rs28362261	3rd	AA	0.017	Same variant
*PCSK9*: rs67608943 (Y142X) [22]	Yes	rs67608943	4th	AA	0.004	Same variant
*PCSK9*: rs72646508 (L253F) [22]	Yes	rs72646508	5th	AA	0.003	Same variant
*APOE*: rs7412 (R176C) [23]	Yes	rs7412	1st	AA, ASN, EUR	0.056–0.110	Same variant
*APOE*: rs769455 (R163C) [31]	Yes	rs769455	2nd	AA	0.020	Same variant
*APOA5*: rs3135506 (S19W) [26]	Yes	rs3135506	1st	AA	0.058	Same variant
*APOA5*: rs651821(-3A>G) [32]	Yes	rs651821	1st	ASN	0.275	Same variant
*APOA5*: rs2075291 (G185C) [25]	Yes	rs2075291	2nd	ASN	0.064	Same variant
*GCKR*: rs1260326 (L446P) [28]	Yes	rs1260326	1st	AA, EUR	0.149–0.350	Same variant
*SORT1*: rs12740374 [18]	Yes	rs12740374	1st	AA	0.247	Same variant
*CETP*: rs17231520 [33]	Yes	rs17231520	3rd	AA	0.069	Same variant
*LIPC*: rs2070895 [34]	Proxy: rs1077834 (LD *r^2^* = 1.00)	rs1077834	1st, 2nd	AA, EUR	0.481	LD *r^2^* = 1.00
*APOB*: rs7575840 [35]	Yes	rs934198	1st	EUR	0.298	LD *r^2^* = 0.98
*LPL*: rs328 (S447X) [36]	Yes	rs1803924	1st	ASN	0.095	LD *r^2^* = 0.96
*LDLR*: rs688 (N591N) [37]	Yes	rs73015011, rs112898275	1st	AA, EUR	----	LD *r^2^*<0.01
*LPL*: rs1801177 (D9N) [38]	Yes	rs75551077, rs15285	1st	AA, EUR	----	LD *r^2^*<0.02
*HMGCR*: rs3761740 (-911C>A) [39]	Proxy: rs17238330 (LD *r^2^* = 1.00)	rs12916	1st	EUR	----	LD *r^2^*<0.20
*LDLR*: -139C>G [40]	No	----	----	----	----	----
*LPL*: rs268 (N291S) [41]	No	----	----	----	----	----
*ABCA1*: rs9282541 (R230C) [42]	No	----	----	----	----	----
*ABCA1*: rs2066715 (V825I) [43]	No	----	----	----	----	----
*LCAT*: rs28940887(R159W) [44]	No	----	----	----	----	----
*PLTP*: R235W [45]	No	----	----	----	----	----
*LIPG*: rs77960347 (A396S) [46]	No	----	----	----	----	----
*LIPG*: rs34474737 [47]	No	----	----	----	----	----

* AA, African American; EUR, European; ASN, East Asian.

Among the 16 reported functional variants and proxies that exhibited the strongest association *P*-value at a signal (Table 5), R176C at *APOE* was strongest in all three populations and *GCKR* L446P was identified in both African Americans and Europeans. The remaining 14 variants showed the strongest associations in only one of the populations, including 10 in African Americans, three in East Asians, and one in Europeans. Five of the 10 variants in African Americans were at the *PCSK9* locus. Furthermore, nine of the 16 variants represented the strongest signal at a given locus, three for a 2nd signal, and four for the 3rd or additional signals. These functional variants covered a wide allele frequency spectrum (MAF: 0.003–0.481), including five less common or rare variants observed only in African Americans.

## Discussion

This study evaluated densely spaced SNPs at 58 lipid loci across three ancestrally diverse populations. The results support evidence that allelic heterogeneity is a frequent feature of polygenic traits [5], [49] and extend the findings to non-European populations, especially to African ancestry populations that have high levels of haplotype diversity. The results also provide strong evidence that fine mapping at GWAS loci can identify population-specific signals. Despite comparable sample sizes, we identified more signals per locus and more signals overall in African Americans (34 signals at 10 loci) compared to Europeans (21 signals at nine loci) and East Asians (nine signals at four loci), and 15 of the 34 signals identified in African Americans were population-specific (Table 1, Table 2, Table 3). These observations may reflect the larger number of SNPs genotyped in African Americans (Table S2), variation across populations subject to natural selection during human evolution [14], or genetic drift [50]. Due to the varied number of signals per locus, different associated markers, and different effect sizes, the phenotypic variance explained differs across populations [51]–[53]. Sampling variability, epistasis, and gene-environment interactions may cause over- or under-estimation of the proportion of explained phenotypic variance. In this study, we also observed that many population-specific signals, including those at *PCSK9* and *APOA5,* are largely confirmatory [20], [22], [54]; however, the association evidence at other signals, in particular the additional signals at *APOE*, *LDLR*, and *APOC1* identified by the conditional analyses, requires replication in future studies.

At *PCSK9*, the strongest signal C679X identified in African Americans is population-specific and showed substantially stronger evidence of association with LDL-C (*P* = 4.1×10^−22^) compared to the GWAS index SNP rs2479409 [5] (*P* = 0.12) and the most strongly associated SNP R46L identified via fine-mapping [7] (*P* = 2.3×10^−3^), both of which were previously reported in Europeans. The proportion of phenotypic variance explained in African Americans increased from 0.16% by the GWAS index SNP to 1.3% by the Metabochip signal C679X, and all variants at the locus together explained 3.6% of the total variation in LDL-C, providing evidence that heritability at identified loci may be underestimated by GWAS [7]. A limitation of these variance estimates is that calculations included the SNPs based simply on their significant association *P* values rather than the variants with biological function, which could over-estimate effects due to the winner's curse.

Results across the genotyped loci demonstrated that the majority of signals were represented by common variants, yet high-density genotyping also identified less common and rare variants associated with lipid traits. At *PCSK9*, the MAFs of six out of the seven signals were <0.05 in African Americans. These signals, along with other low frequency variants identified at *APOE*, *LDLR*, *LCAT*, *APOB*, *APOC1*, and *LPL* provide evidence of the substantial contribution of low frequency genetic variants to the variance of lipid traits [6]. Other variants, some with very low allele frequency, may exist at these loci, suggesting that future sequencing studies may identify additional functional variants that influence lipid variation.

Sequential conditional analyses provided further insight into the genetic architecture of the established lipid loci by explaining additional phenotypic variation and revealing complex patterns of association. We observed loci at which signals were not independent of each other, but partially correlated based on moderate LD estimates and changes of association statistics before and after accounting for other signals. For these dependent signals, such as those at *TOMM4-APOE-APOC4*, the significance of residual association would increase when trait-increasing alleles were present on opposite haplotypes and decrease when trait-increasing alleles were on the same haplotype. Other signals that appeared to be independent on the basis of low pairwise LD and unchanged association evidence after conditional analysis may still be partially tagging an un-typed, yet influential, variant [55]–[57]. Therefore, deeper sequencing that identifies all variants at a locus will be required to characterize more fully the allelic heterogeneity and the patterns of association.

One of the major goals of high-density genotyping is to aid in identification of the functional variants by recognizing the most compelling candidate variants for experimental study. Because of the diverse LD structure across populations, particularly in terms of the limited LD extent in African ancestry populations, trans-ethnic fine-mapping of GWAS loci can narrow the region where functional variants are most likely to reside. This study was able to narrow the association signals at five lipid loci, based on the much smaller subsets of most strongly associated variants located in smaller regions. One signal was localized to a reported causal variant (*GCKR*-P446L) [28] and another to an uncharacterized nonsynonymous variant (*SLC12A4*-E4G near *LCAT*). These findings demonstrate that trans-ethnic association analyses can increase the resolution of fine-mapping by enlarging the haplotypic diversity of samples with different ancestries and consequently, narrowing the sets of candidate functional variants [58], [59]. The previously described functional variants at *LCAT* [44] and *ABCA1* [42], [43], which are not present on the Metabochip, were physically located 22 kb and >43 kb away from the narrowed association signals observed in this study (Table 4).

Refining signals by trans-ethnic meta-analysis largely relies not only on the existence of distinct LD patterns across ancestry groups but also on shared functional variants. If functional variants are shared across populations, as observed with *GCKR*-P446L, performing trans-ethnic meta-analysis and integrating LD information across different populations may refine the signal. On the contrary, if trait variation is influenced by distinct functional variants across populations, as our data suggest for *APOA5* (Figure S6A–S6D), the lead SNPs produced by meta-analysis would be influenced by the sample size, magnitude of genetic effects, and allele frequencies. Similarly, in the case of population-specific functional variants, such as those at *PCSK9*, the results from meta-analysis would reflect the association in one particular population rather than the combined effect across populations if signals unique to this population drive the results. Therefore, accurate assessment of allelic variability is needed on a population-by-population and locus-by-locus basis.

Although genotype imputation has become a standard practice to increase genome coverage in GWAS by predicting the genotypes at SNPs that are not directly genotyped, imputation accuracy tends to be lower for rare variants owing to the lower degree of LD and the more challenging haplotype reconstruction [60]. In addition, African American samples pose a challenge for imputation due to their varying degree of admixture [61]. A major strength of our study is that all variants we tested for association were directly genotyped using the Metabochip, which was designed to provide a high-density coverage for both overall SNPs and low frequency variants concentrated around GWAS-identified loci and/or signals [9], [10]. This approach increases the reliability of our association results overall, but in particular the variants with low allele frequencies.

In conclusion, we performed a large-scale trans-ethnic fine-mapping study to investigate the established lipid loci using the Metabochip high-density genotyping array and focusing on diverse groups including African Americans, East Asians, and Europeans. Our results highlight the value of high-density genotyping in diverse populations to identify a wider spectrum of susceptibility variants at established loci, both in terms of additional signals and in terms of population-specific and/or potentially functional variants. The additional signals revealed through the sequential conditional analyses lead to a 1.3- to 1.8-fold increase in the explained phenotypic variance across the different populations. In addition, integrating diverse LD patterns across diverse ancestry groups allows for the refinement of association signals. Lastly, our findings that 74% of the reported functional variants exhibited the strongest association at these densely typed signals suggest that at loci and signals where functional variants are unknown, the variants with strongest association may be good candidates for functional assessment.

## Materials and Methods

### Study populations and phenotypes

The 6,832 African Americans studied are comprised of individuals from the Atherosclerosis Risk in Communities Study (ARIC) [62], the Multiethnic Cohort Study (MEC) [63], and the Women's Health Initiative (WHI) [64], [65] that are part of Population Architecture using Genomics and Epidemiology (PAGE) consortium [66] and from Hypertensive Genetic Epidemiology Network (HyperGEN) [67]. The 9,449 East Asian samples are comprised of 1,716 Filipinos from the Cebu Longitudinal Health and Nutrition Survey (CLHNS) [68] and 7,733 Chinese from Taiwan-Metabochip Study for Cardiovascular Disease (TAICHI). The 10,829 European samples are comprised of Finnish and Norwegian individuals; the Finns are from the Finland-United States Investigation of NIDDM Genetics (FUSION), Dehko 2D 2007 (D2D2007), Diabetes Prevention Study (DPS), Dose-Responses to Exercise Training (DR's EXTRA), and Metabolic Syndrome in Men (METSIM) [69], [70], and the Norwegians were from the cohorts of Nord-Trøndelag Health Study (HUNT 2) and the Tromsø Study (TROMSO) [71], [72].

All study protocols were approved by Institutional Review Boards at their respective sites. Brief descriptions of the studies are provided in the Text S1. General characteristics and measurements of TG, HDL-C, and LDL-C in each cohort are summarized in Table S1. Values of triglycerides were natural log transformed to approximate normality in each study sample separately.

### Genotyping

We genotyped all study samples with the Metabochip according to the manufacturer's protocol (Illumina, San Diego, CA, USA). Table S1 summarizes the quality control criteria of genotyping, including call rate, sample success rate, Hardy-Weinberg equilibrium, and MAF that varied across studies.

### Statistical analyses

We applied multiple linear regression models and assumed an additive mode of inheritance to test for association between genotypes and HDL-C, LDL-C, or log-transformed triglycerides. We performed each test of association separately in each of the 11 groups (Table S1) prior to meta-analysis. We constructed principal components (PCs) using the software EIGENSOFT. We used age and sex as covariates in each individual cohort; other cohort-specific covariates including age^2^, enrollment site, socioeconomic status, and principal components varied across studies (Table S1). The European samples include type 2 diabetes (T2D) cases and unaffected controls; to avoid confounding due to T2D status, samples were analyzed separately as Finnish T2D patients, Finnish unaffected individuals, Norwegian T2D patients, and Norwegian unaffected individuals.

We first conducted the meta-analysis within the African Americans, East Asians, and Europeans separately. We then performed combined trans-ethnic meta-analyses by combining the statistics of each the 11 participating groups to assess the association with the SNPs at the 58 lipids loci.

At loci that exhibited evidence of association at *P*<10^−4^, we next performed a series of sequential conditional analyses by adding the most strongly associated SNP into the regression model as a covariate and testing all remaining regional SNPs for association. We conducted a set of sequential conditional analyses until the strongest SNP showed a conditional *P* value>10^−4^ and had no annotation or literature evidence that suggested a functional role.

For single SNP analyses, we applied PLINK (http://pngu.mgh.harvard.edu/~purcell/plink/) [73] for population-based studies. We used the R package GWAF [74] for the family-based study of HyperGEN. We applied an inverse variance-weighted fixed-effect meta-analysis implemented in METAL [75].

Unless otherwise noted, linkage disequilibrium estimates were obtained from the 1000 Genomes Project November 2010 release. SNP positions correspond to hg18.

We performed haplotype analysis at LDL-C locus *TOMM40-APOE-APOC4* in 5,593 unrelated African Americans from the PAGE consortium, using the ‘haplo.stat’ R package. Haplotypes and haplotype frequencies were estimated using the R function ‘haplo.em’. The association between haplotypes and LDL-C was assessed using the R function ‘haplo.glm’. An additive model was assumed, in which the regression coefficient β represents the expected change in LDL-C level with each additional copy of the specific haplotype compared with the reference haplotype, which was set as the A-A (trait increasing-increasing) haplotype.

We created the regional association plots using LocusZoom [76]. To plot the association results in Europeans and East Asians, we used the LocusZoom-implemented LD estimates from the 1000 Genomes Project (June 2010) CEU and CHB+JPT samples, whose LD structures are similar to our samples with European and East Asian ancestries. We applied the user-supplied LD calculated from the genotype data of the PAGE African American samples to plot the regional association in African Americans [9], because the LD patterns may vary from any pre-computed LD sources implemented in LocusZoom.

We evaluated the proportion of variance explained by a single SNP or any given locus by including the SNP or a set of SNPs into a linear regression model with all covariates used in association analysis and calculating the R^2^ for the full model. We subtracted the variance explained by a basic model in which only covariates were included from the variance we obtained from the full model. We performed these analyses using SAS version 9.2 (SAS Institute, Cary, NC, USA).

## Supporting Information

Figure S1 LDL-C locus *TOMM40-APOE-APOC4* exhibited seven signals in African Americans. Each SNP was colored according to its LD (*r^2^*) in PAGE consortium with the strongest SNP rs7412 (R176C) colored in purple.
(PDF)

Figure S2 Association at TG locus *GCKR* in Europeans (A), East Asians (B), African Americans (C), and trans-ethnic meta-analysis (D). Index SNP rs1260326 (P446L) is the variant showing the strongest evidence of association in trans-ethnic meta-analysis.
(PDF)

Figure S3 Association at LDL-C locus *ABO* in Europeans (A), East Asians (B), African Americans (C), and trans-ethnic meta-analysis (D). Index SNP rs2519093 is the variant showing the strongest evidence of association in trans-ethnic meta-analysis.
(PDF)

Figure S4 Association at HDL-C locus *LCAT* in Europeans (A), East Asians (B), African Americans (C), and trans-ethnic meta-analysis (D). Index SNP rs3785100 (*SLC12A4*-E4G) is the variant showing the strongest evidence of association in trans-ethnic meta-analysis.
(PDF)

Figure S5 Association at HDL-C locus *ABCA1* in Europeans (A), East Asians (B), African Americans (C), and trans-ethnic meta-analysis (D). Index SNP rs1883025 is the variant showing the strongest evidence of association in trans-ethnic meta-analysis.
(PDF)

Figure S6 Association at TG locus *APOA5* in Europeans (A), East Asians (B), African Americans (C), and trans-ethnic meta-analysis (D). The SNPs rs3741298, rs651821 (-3A>G), rs3135506 (S19W), and rs662799 that exhibited the smallest *P* values in Europeans, East Asians, African Americans, and the trans-ethnic meta-analysis are indicated.
(PDF)

Table S1 Characteristics of the study samples.
(PDF)

Table S2 Number of SNPs at each locus for analysis in each of the three ancestry groups.
(PDF)

Table S3 Lead SNP at TG (A), HDL-C (B), and LDL-C (C) loci within each ancestry group and their relative significance compared to reported GWAS index SNPs.
(PDF)

Table S4 SNPs with the strongest association at TG (A), HDL-C (B) and LDL-C (C) loci in combined trans-ethnic meta-analysis and their associations within ancestry groups.
(PDF)

Table S5 LDL-C association with haplotypes consisting of the third (rs1038026) and the fourth (rs157588) signals at *TOMM40-APOE-APOC4* cluster.
(PDF)

Text S1 Study description.
(DOCX)
