# Supplementary material for: Trans-Ethnic Fine-Mapping of Lipid Loci Identifies Population-Specific Signals and Allelic Heterogeneity That Increases the Trait Variance Explained
Source: PLoS Genet. 2013 Mar 21;9(3):e1003379. doi: 10.1371/journal.pgen.1003379 (PMC3605054; doi:10.1371/journal.pgen.1003379)
Supplement: Figure S1 — LDL-C locus TOMM40-APOE-APOC4 exhibited seven signals in African Americans. Each SNP was colored according to its LD (r2) in PAGE consortium with the strongest SNP rs7412 (R176C) colored in purple. (PDF) [file pgen.1003379.s001.pdf]

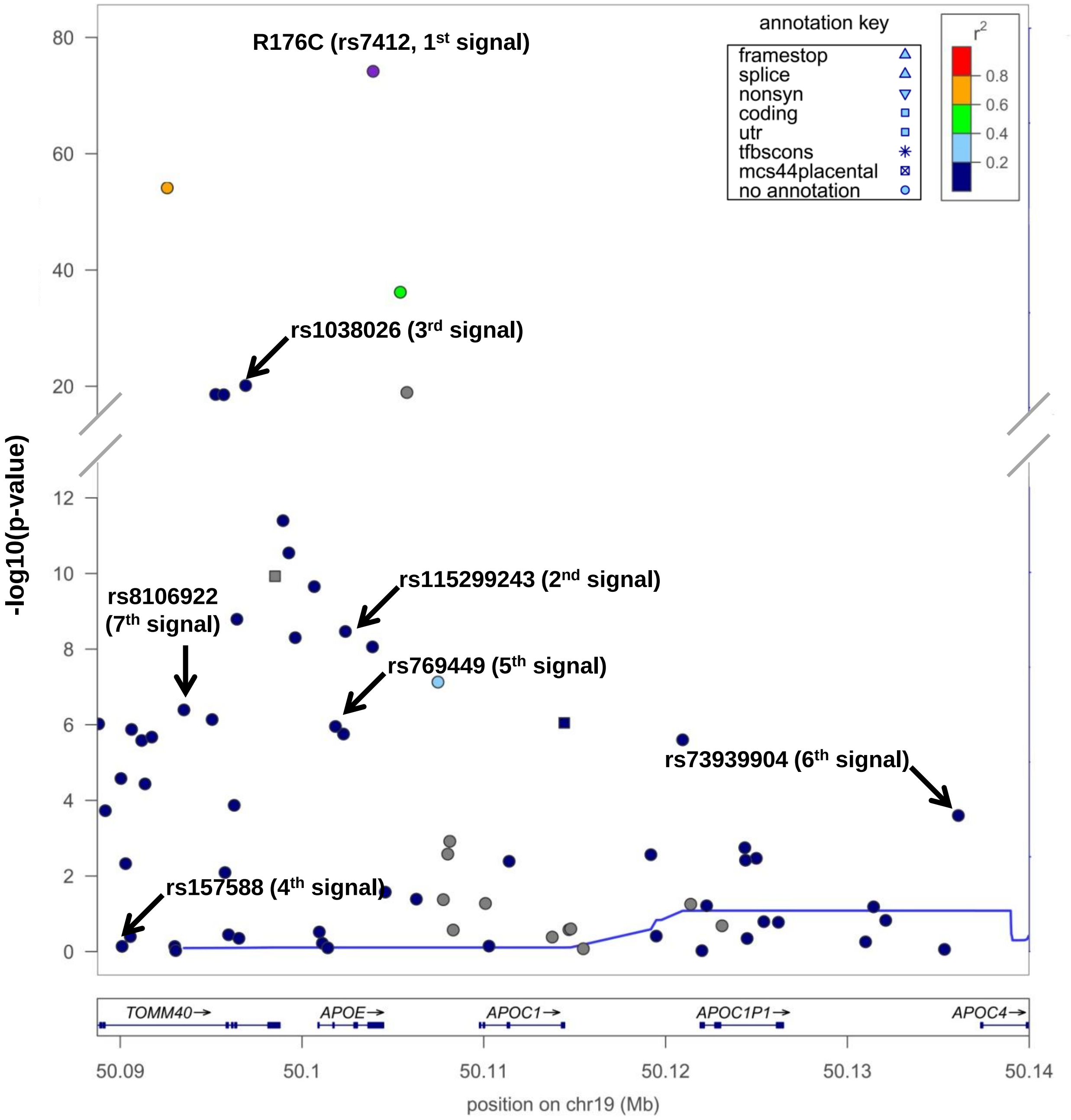

Figure S1. LDL-C locus *TOMM40-APOE-APOC4* exhibited seven signals in African Americans. Each SNP was colored according to its LD (r^2) in PAGE consortium with the strongest SNP rs7412 (R176C) colored in purple.
