# Supplementary material for: Trans-Ethnic Fine-Mapping of Lipid Loci Identifies Population-Specific Signals and Allelic Heterogeneity That Increases the Trait Variance Explained
Source: PLoS Genet. 2013 Mar 21;9(3):e1003379. doi: 10.1371/journal.pgen.1003379 (PMC3605054; doi:10.1371/journal.pgen.1003379)
Supplement: Figure S2 — Association at TG locus GCKR in Europeans (A), East Asians (B), African Americans (C), and trans-ethnic meta-analysis (D). Index SNP rs1260326 (P446L) is the variant showing the strongest evidence of association in trans-ethnic meta-analysis. (PDF) [file pgen.1003379.s002.pdf]

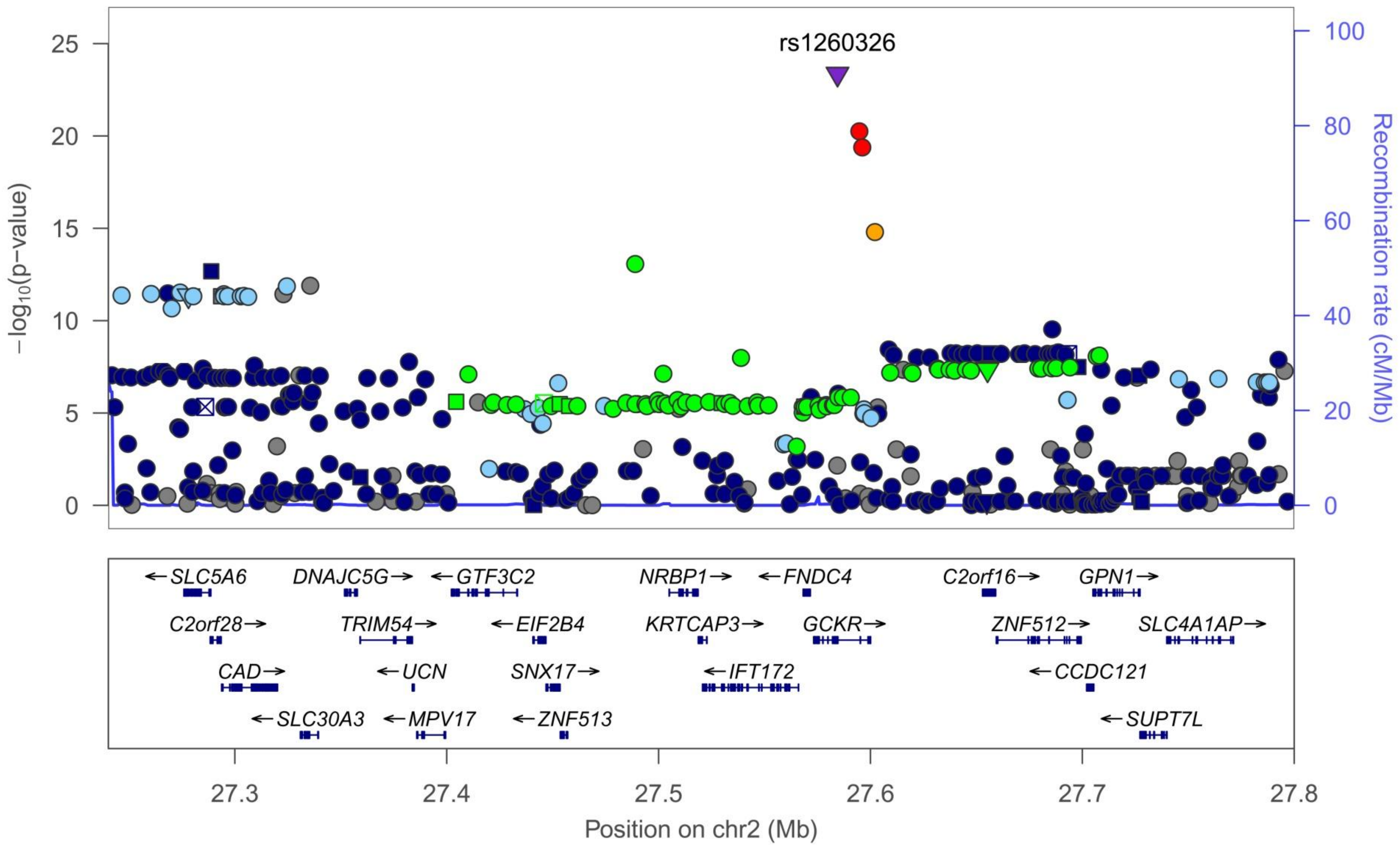

Figure S2A. Association at TG locus *GCKR* in Europeans. Index SNP rs1260326 (P446L) is the variant showing the strongest evidence of association in trans-ethnic meta-analysis.

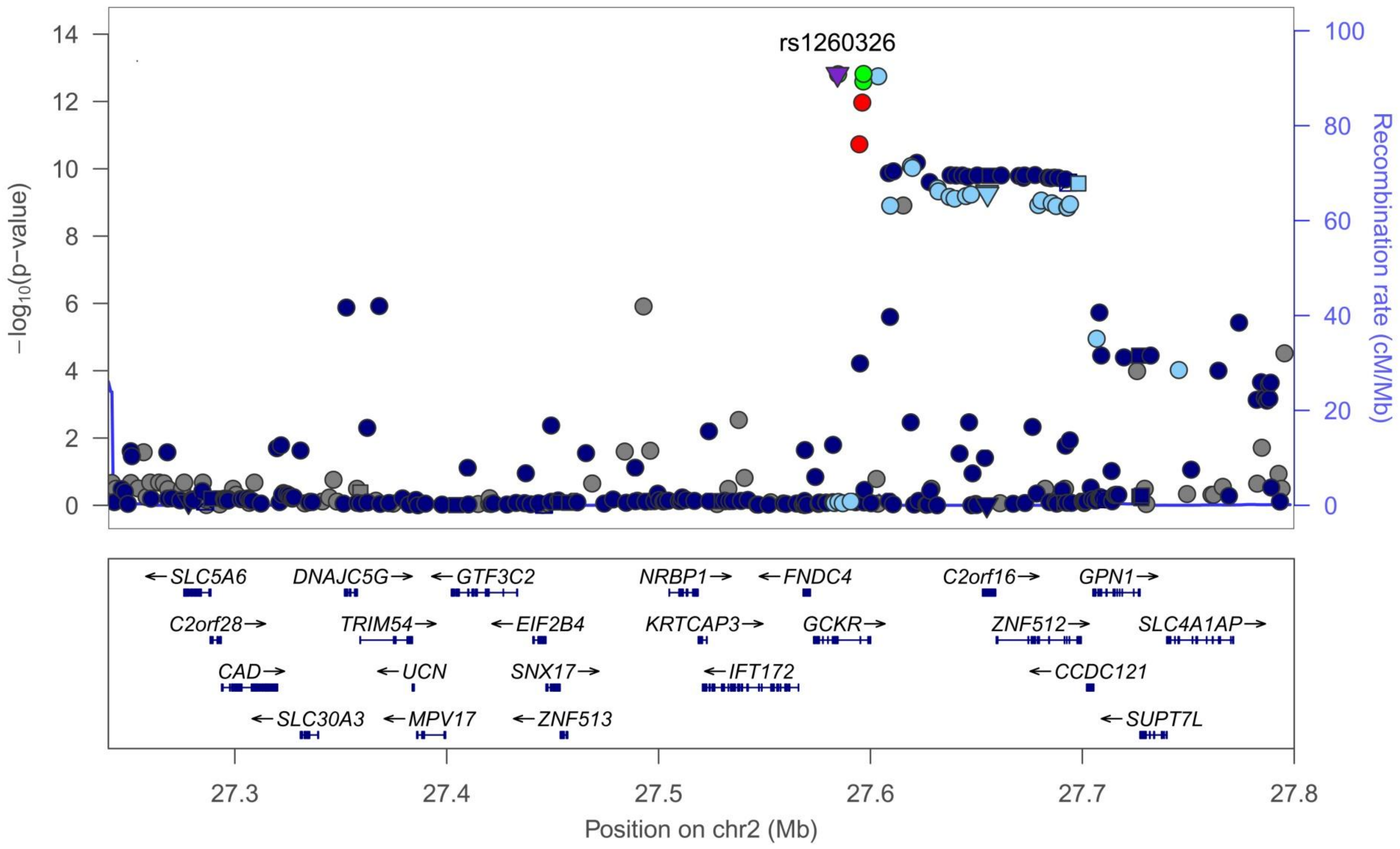

Figure S2B. Association at TG locus *GCKR* in East Asians. Index SNP rs1260326 (P446L) is the variant showing the strongest evidence of association in trans-ethnic meta-analysis.

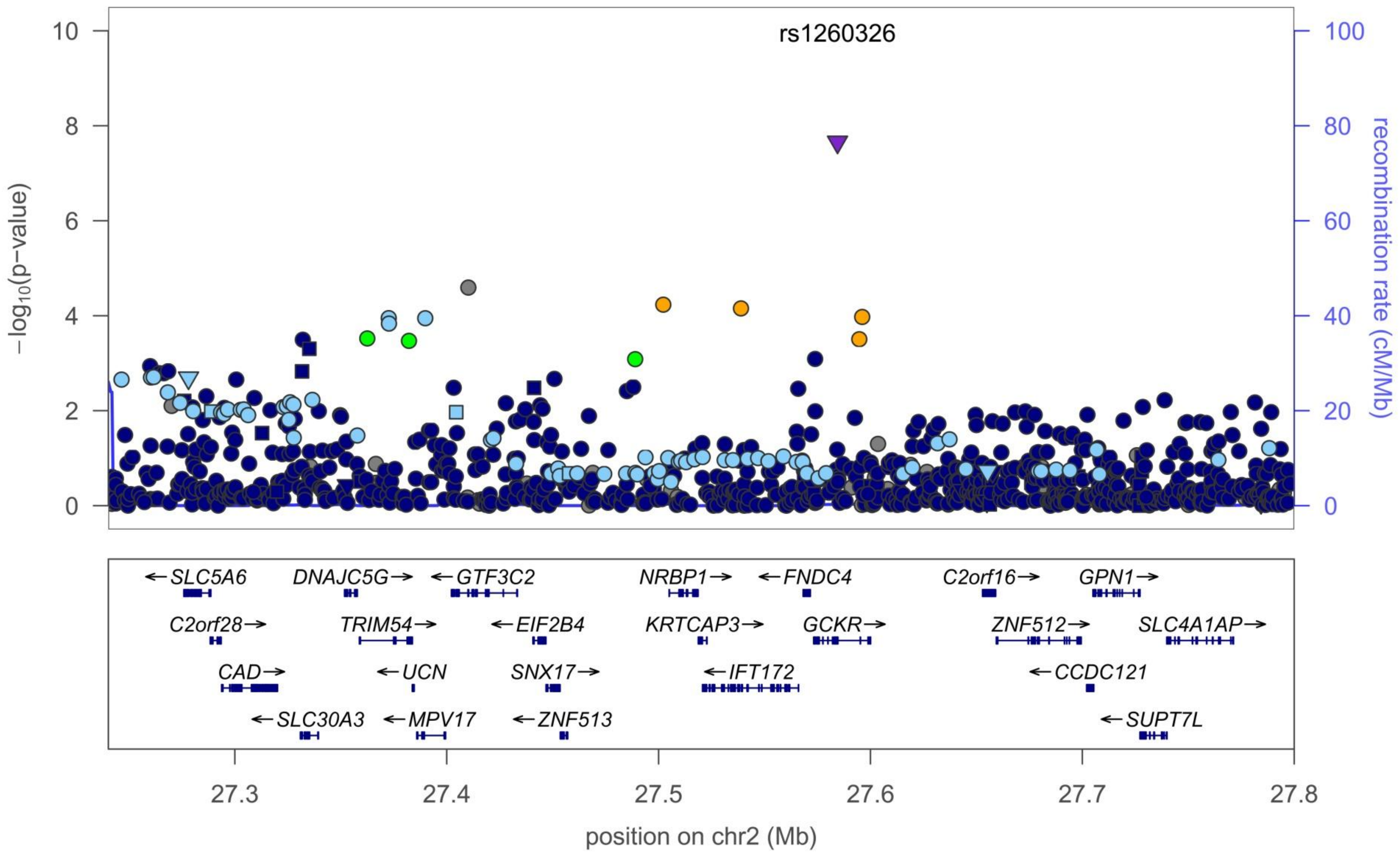

Figure S2C. Association at TG locus *GCKR* in African Americans. Index SNP rs1260326 (P446L) is the variant showing the strongest evidence of association in trans-ethnic meta-analysis.

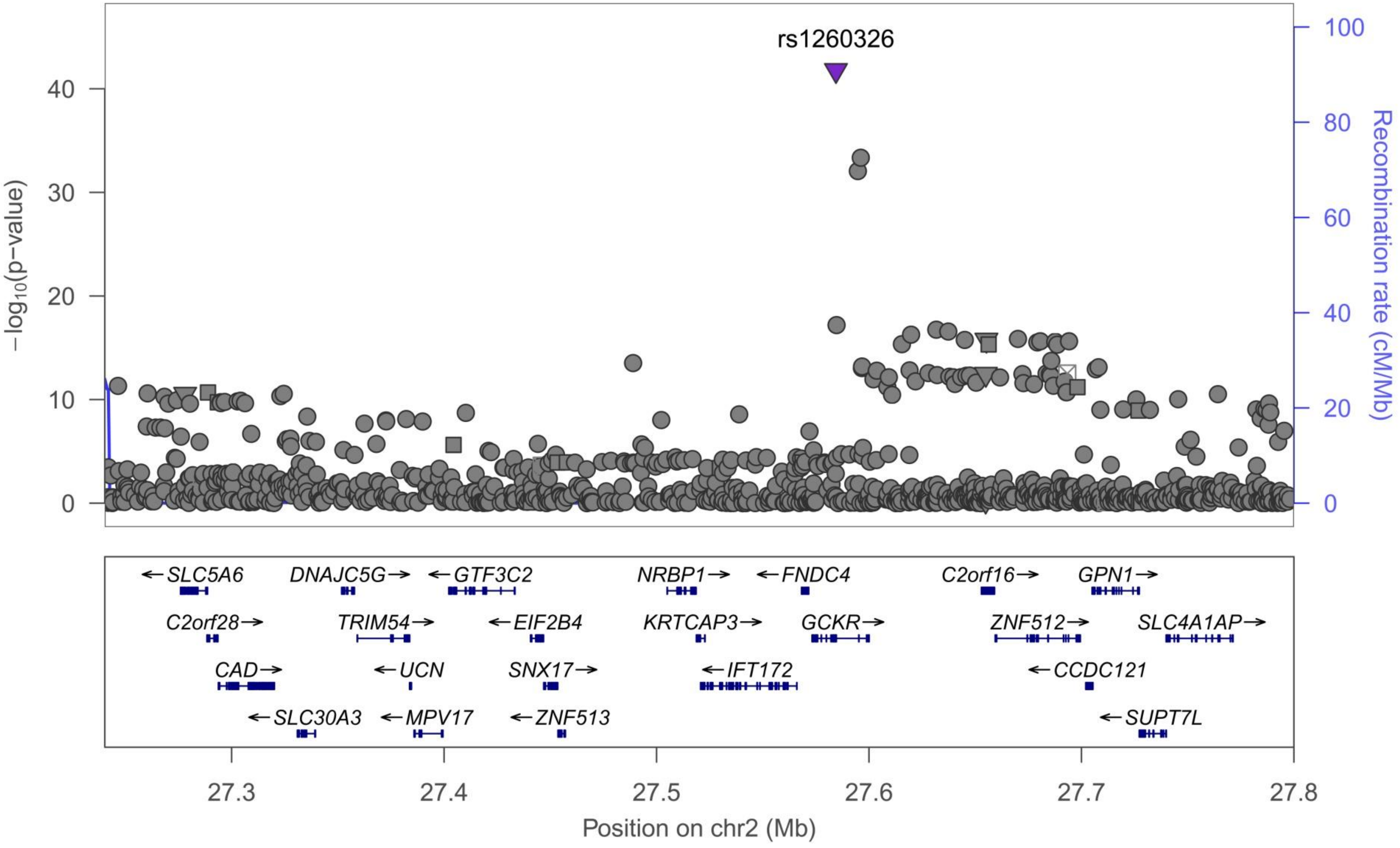

Figure S2D. Association at TG locus *GCKR* in trans-ethnic meta-analysis.
