# Supplementary material for: Trans-Ethnic Fine-Mapping of Lipid Loci Identifies Population-Specific Signals and Allelic Heterogeneity That Increases the Trait Variance Explained
Source: PLoS Genet. 2013 Mar 21;9(3):e1003379. doi: 10.1371/journal.pgen.1003379 (PMC3605054; doi:10.1371/journal.pgen.1003379)
Supplement: Figure S3 — Association at LDL-C locus ABO in Europeans (A), East Asians (B), African Americans (C), and trans-ethnic meta-analysis (D). Index SNP rs2519093 is the variant showing the strongest evidence of association in trans-ethnic meta-analysis. (PDF) [file pgen.1003379.s003.pdf]

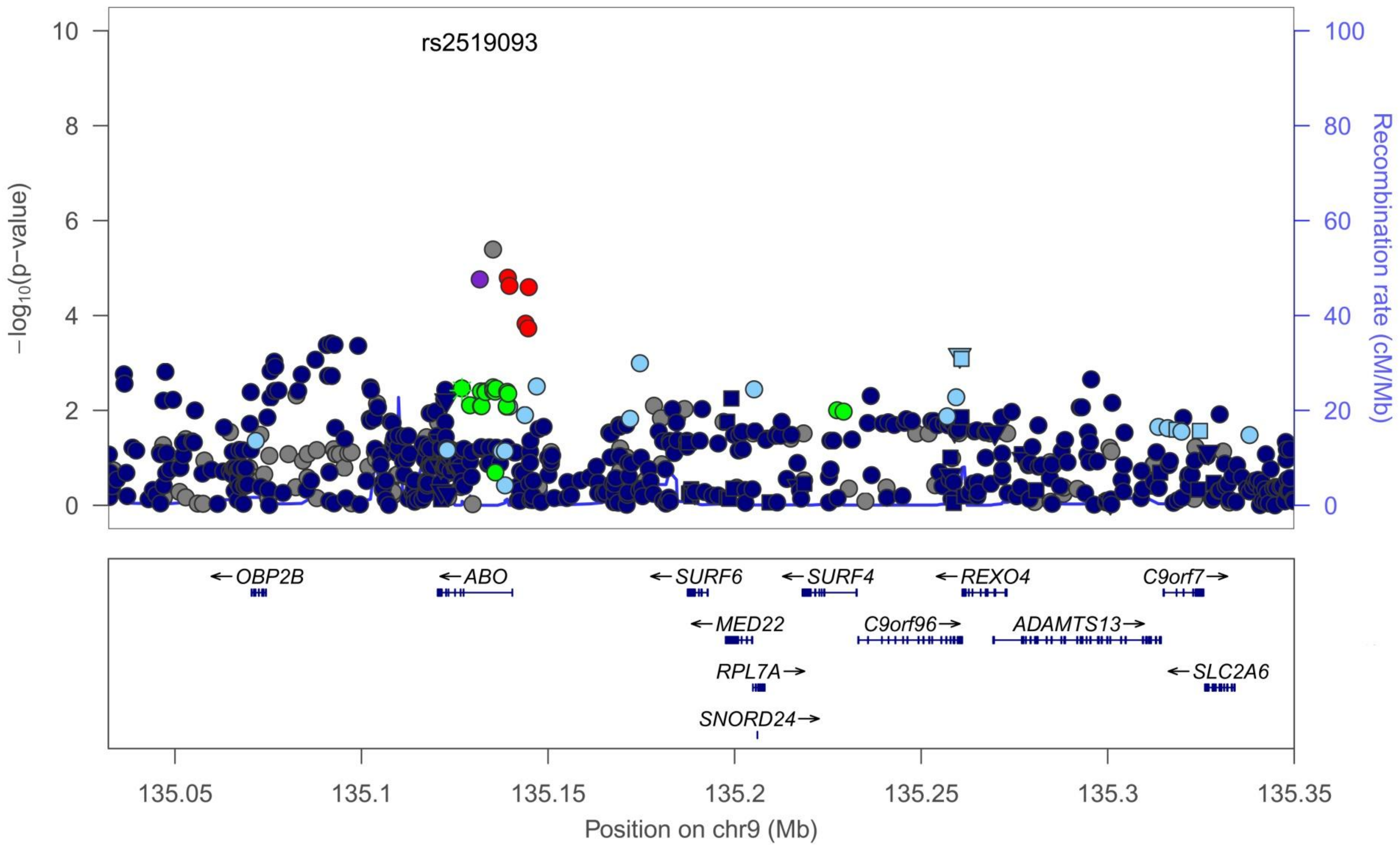

Figure S3A. Association at LDL-C locus *ABO* in Europeans. Index SNP rs2519093 is the variant showing the strongest evidence of association in trans-ethnic meta-analysis.

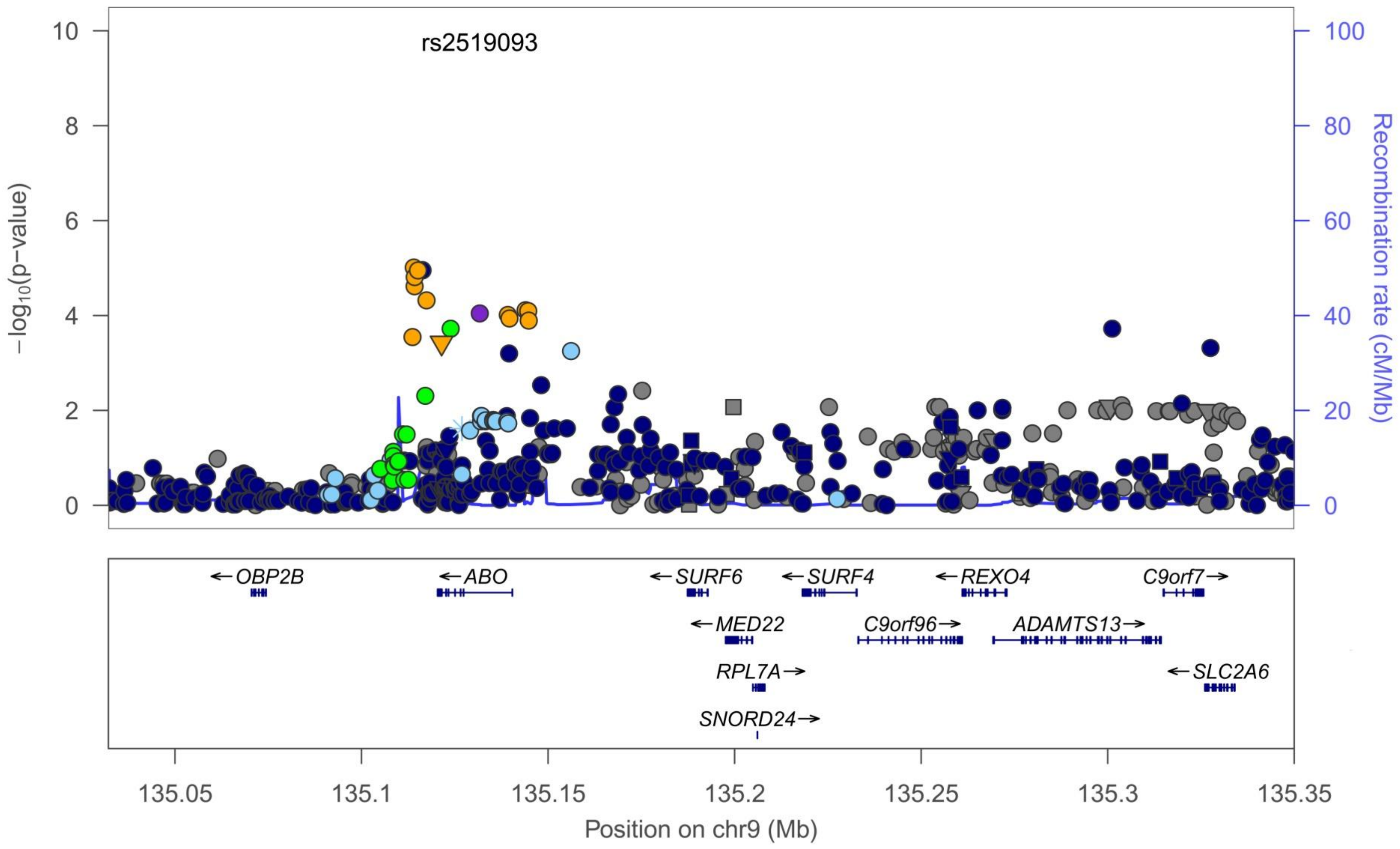

Figure S3B. Association at LDL-C locus *ABO* in East Asians. Index SNP rs2519093 is the variant showing the strongest evidence of association in trans-ethnic meta-analysis.

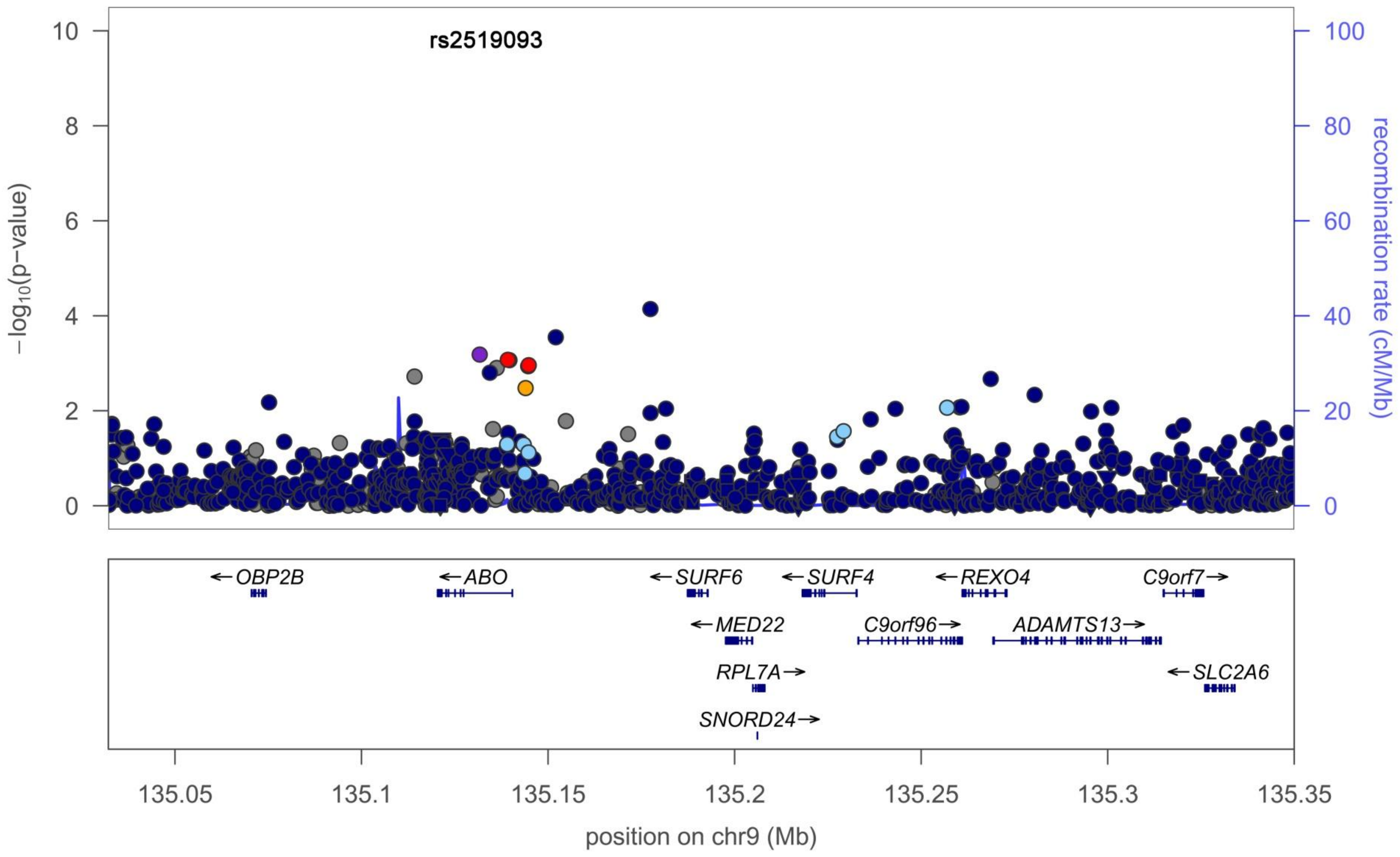

Figure S3C. Association at LDL-C locus *ABO* in African Americans. Index SNP rs2519093 is the variant showing the strongest evidence of association in trans-ethnic meta-analysis.

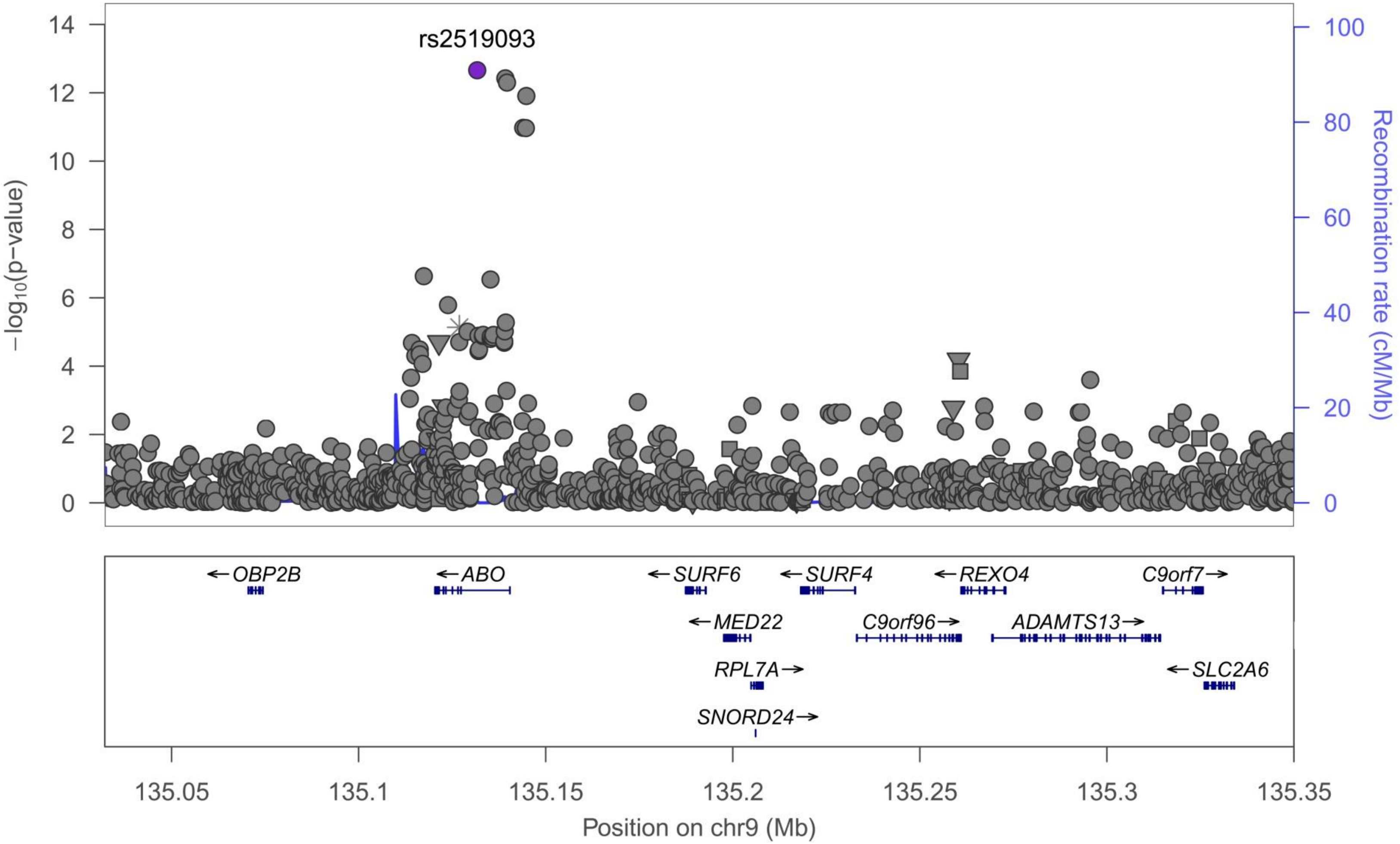

Figure S3D. Association at LDL-C locus *ABO* in trans-ethnic meta-analysis.
