# Supplementary material for: Trans-Ethnic Fine-Mapping of Lipid Loci Identifies Population-Specific Signals and Allelic Heterogeneity That Increases the Trait Variance Explained
Source: PLoS Genet. 2013 Mar 21;9(3):e1003379. doi: 10.1371/journal.pgen.1003379 (PMC3605054; doi:10.1371/journal.pgen.1003379)
Supplement: Figure S4 — Association at HDL-C locus LCAT in Europeans (A), East Asians (B), African Americans (C), and trans-ethnic meta-analysis (D). Index SNP rs3785100 (SLC12A4-E4G) is the variant showing the strongest evidence of association in trans-ethnic meta-analysis. (PDF) [file pgen.1003379.s004.pdf]

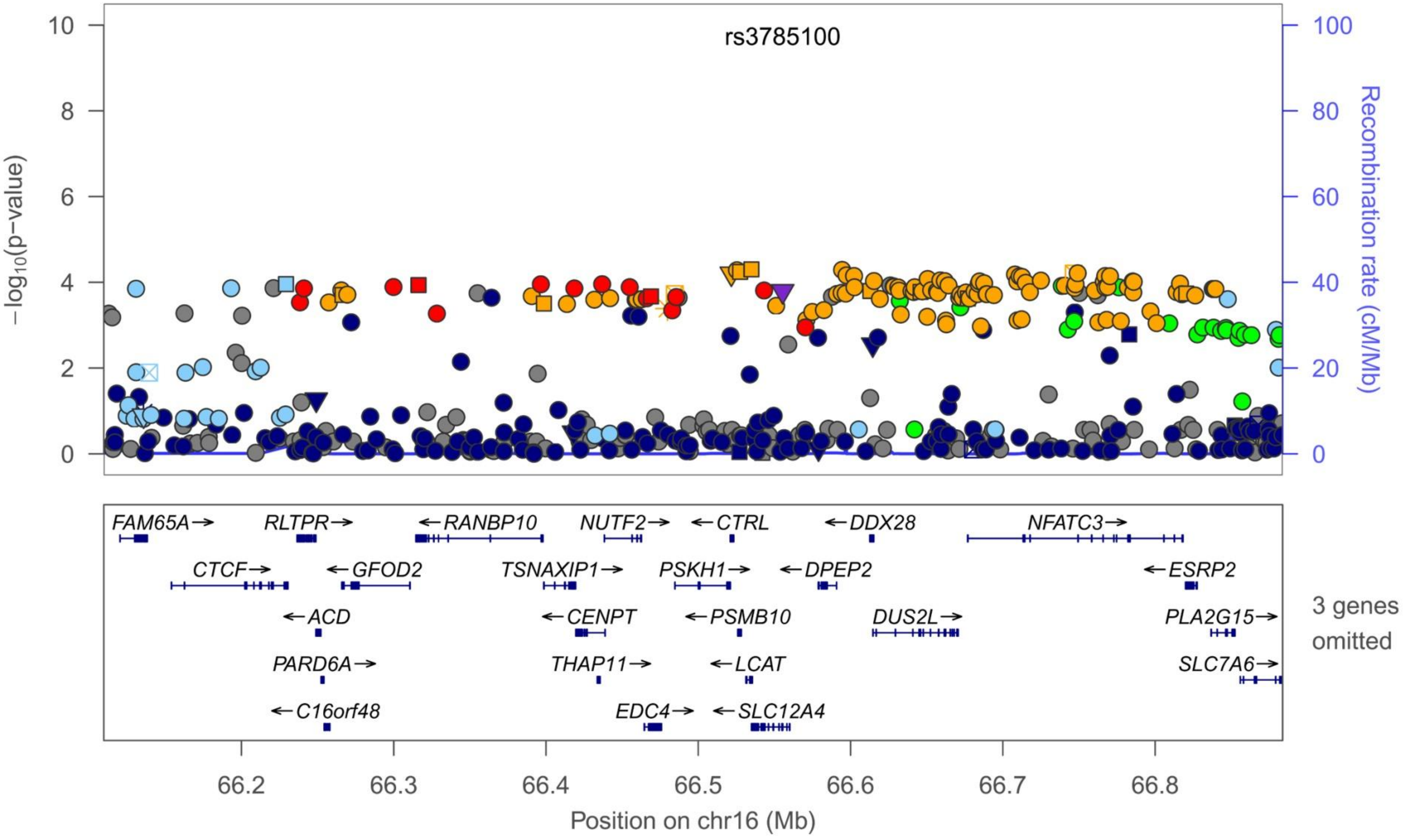

Figure S4A. Association at HDL-C locus *LCAT* in Europeans. Index SNP rs3785100 (*SLC12A4*-E4G) is the variant showing the strongest evidence of association in trans-ethnic meta-analysis.

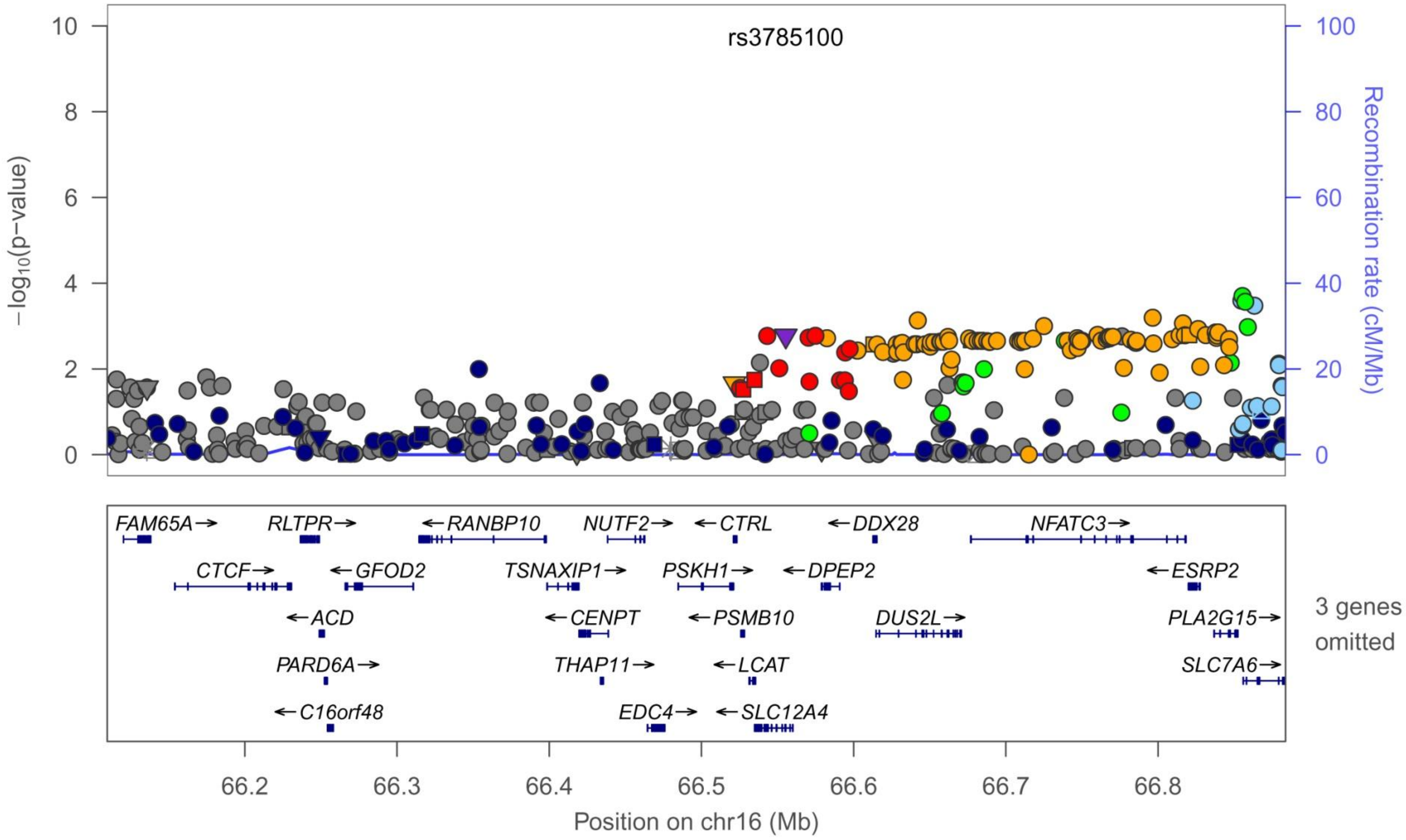

Figure S4B. Association at HDL-C locus *LCAT* in East Asians. Index SNP rs3785100 (*SLC12A4*-E4G) is the variant showing the strongest evidence of association in trans-ethnic meta-analysis.

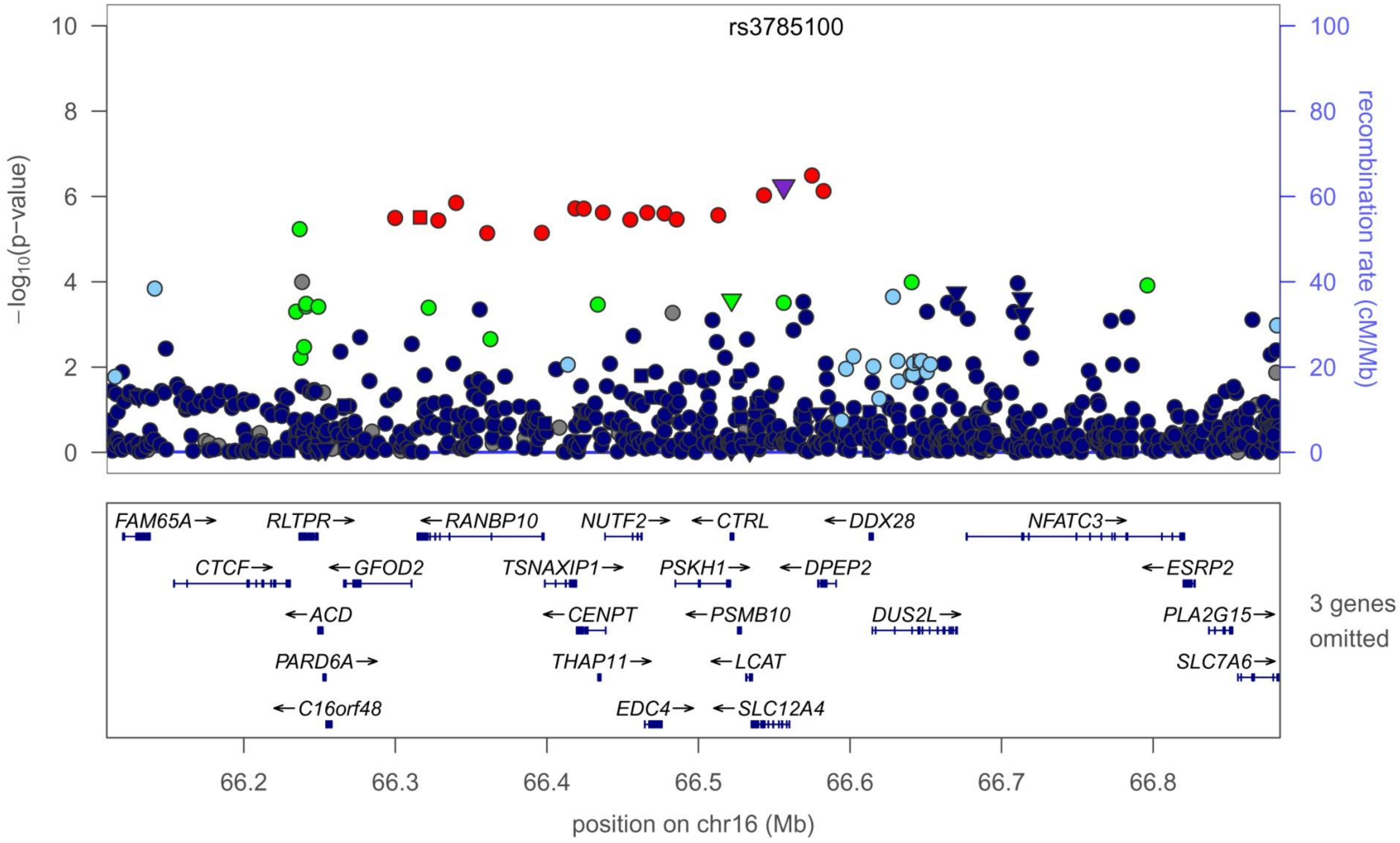

Figure S4C. Association at HDL-C locus *LCAT* in African Americans. Index SNP rs3785100 (*SLC12A4*-E4G) is the variant showing the strongest evidence of association in trans-ethnic meta-analysis.

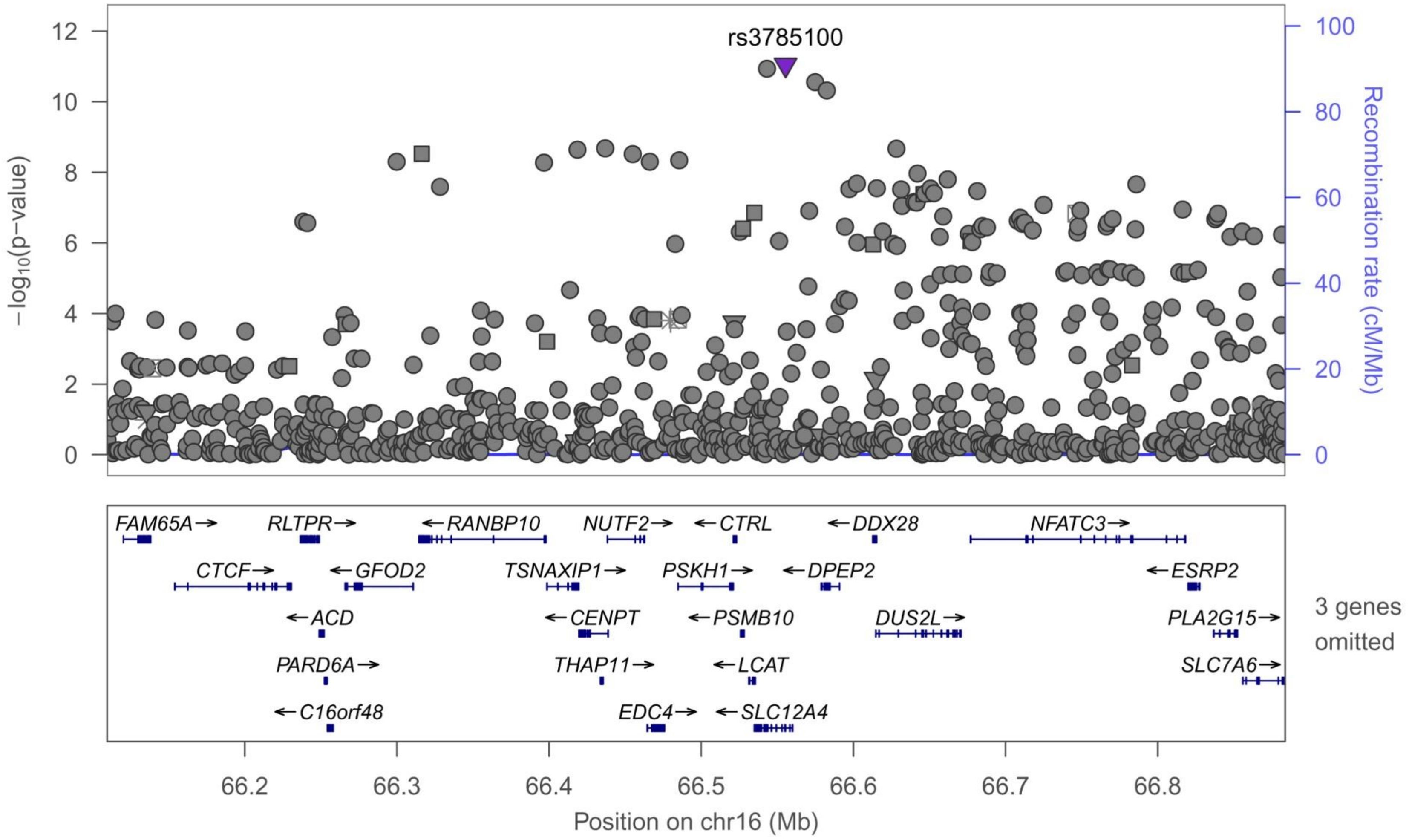

Figure S4D. Association at HDL-C locus *LCAT* in trans-ethnic meta-analysis.
