# Supplementary material for: Trans-Ethnic Fine-Mapping of Lipid Loci Identifies Population-Specific Signals and Allelic Heterogeneity That Increases the Trait Variance Explained
Source: PLoS Genet. 2013 Mar 21;9(3):e1003379. doi: 10.1371/journal.pgen.1003379 (PMC3605054; doi:10.1371/journal.pgen.1003379)
Supplement: Figure S5 — Association at HDL-C locus ABCA1 in Europeans (A), East Asians (B), African Americans (C), and trans-ethnic meta-analysis (D). Index SNP rs1883025 is the variant showing the strongest evidence of association in trans-ethnic meta-analysis. (PDF) [file pgen.1003379.s005.pdf]

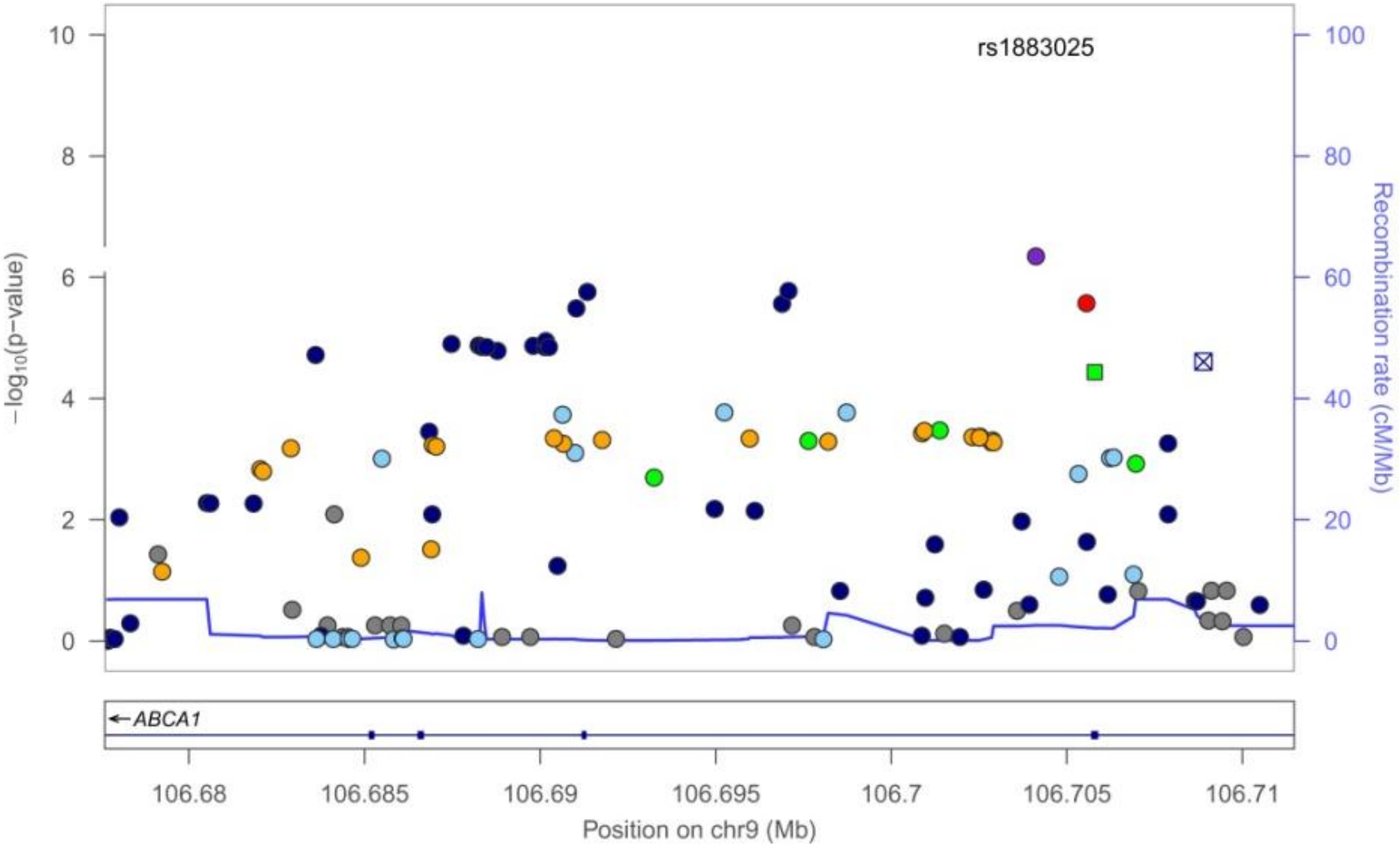


**Figure S5A.** Association at HDL-C locus *ABCA1* in Europeans. Index SNP rs1883025 is the variant showing the strongest evidence of association in trans-ethnic meta-analysis.

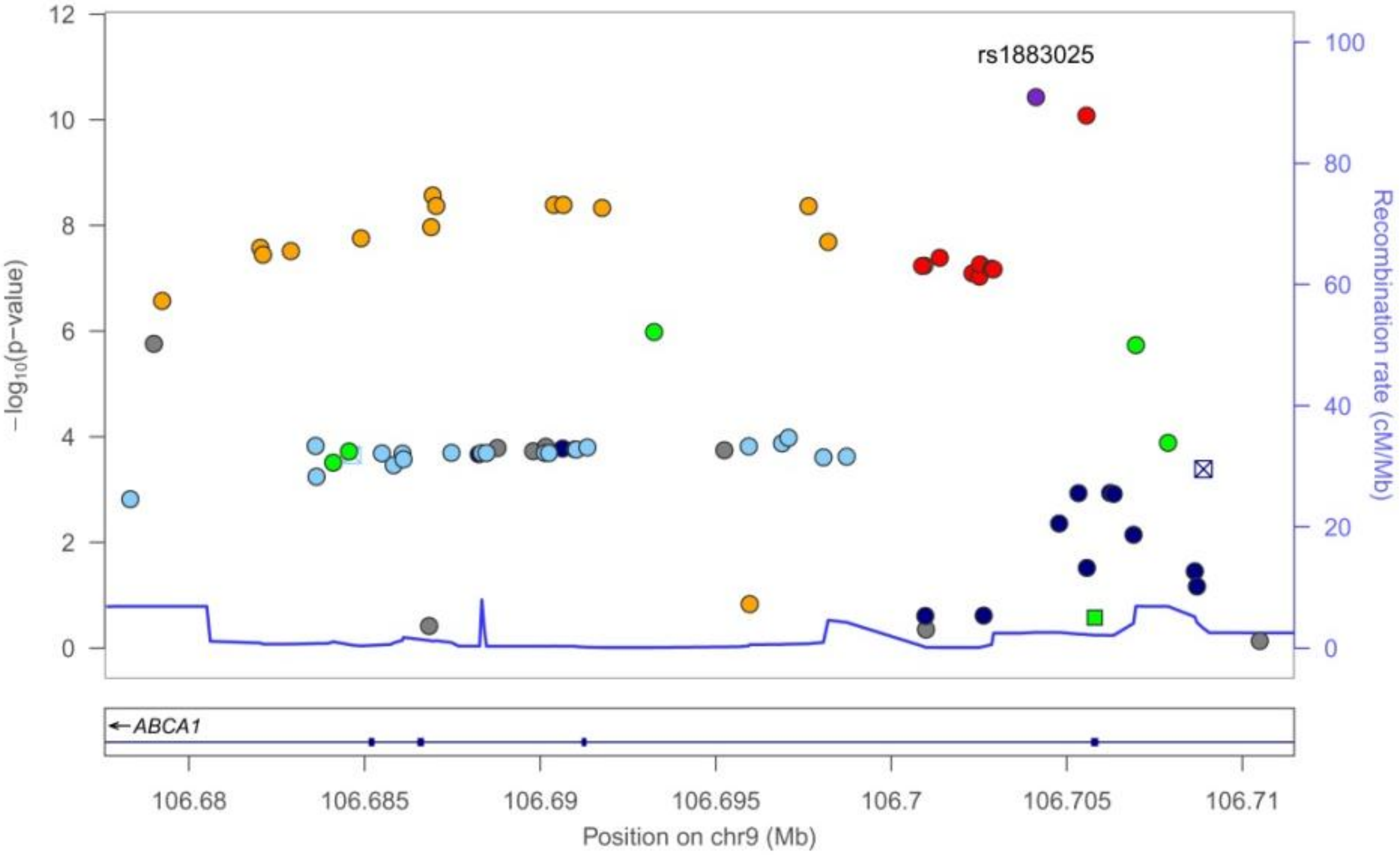


**Figure S5B.** Association at HDL-C locus *ABCA1* in East Asians. Index SNP rs1883025 is the variant showing the strongest evidence of association in trans-ethnic meta-analysis.

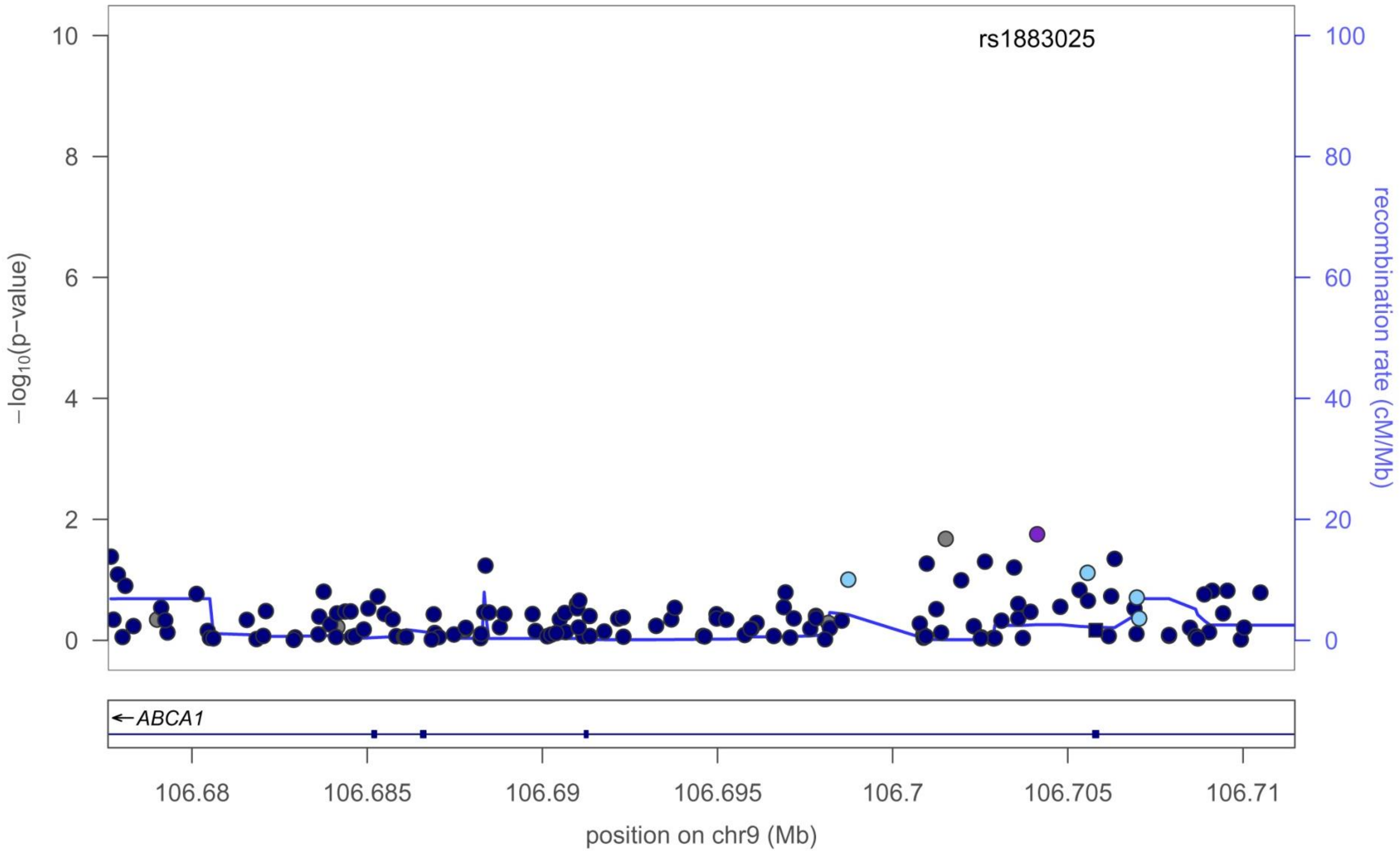


**Figure S5C.** Association at HDL-C locus *ABCA1* in African Americans. Index SNP rs1883025 is the variant showing the strongest evidence of association in trans-ethnic meta-analysis.

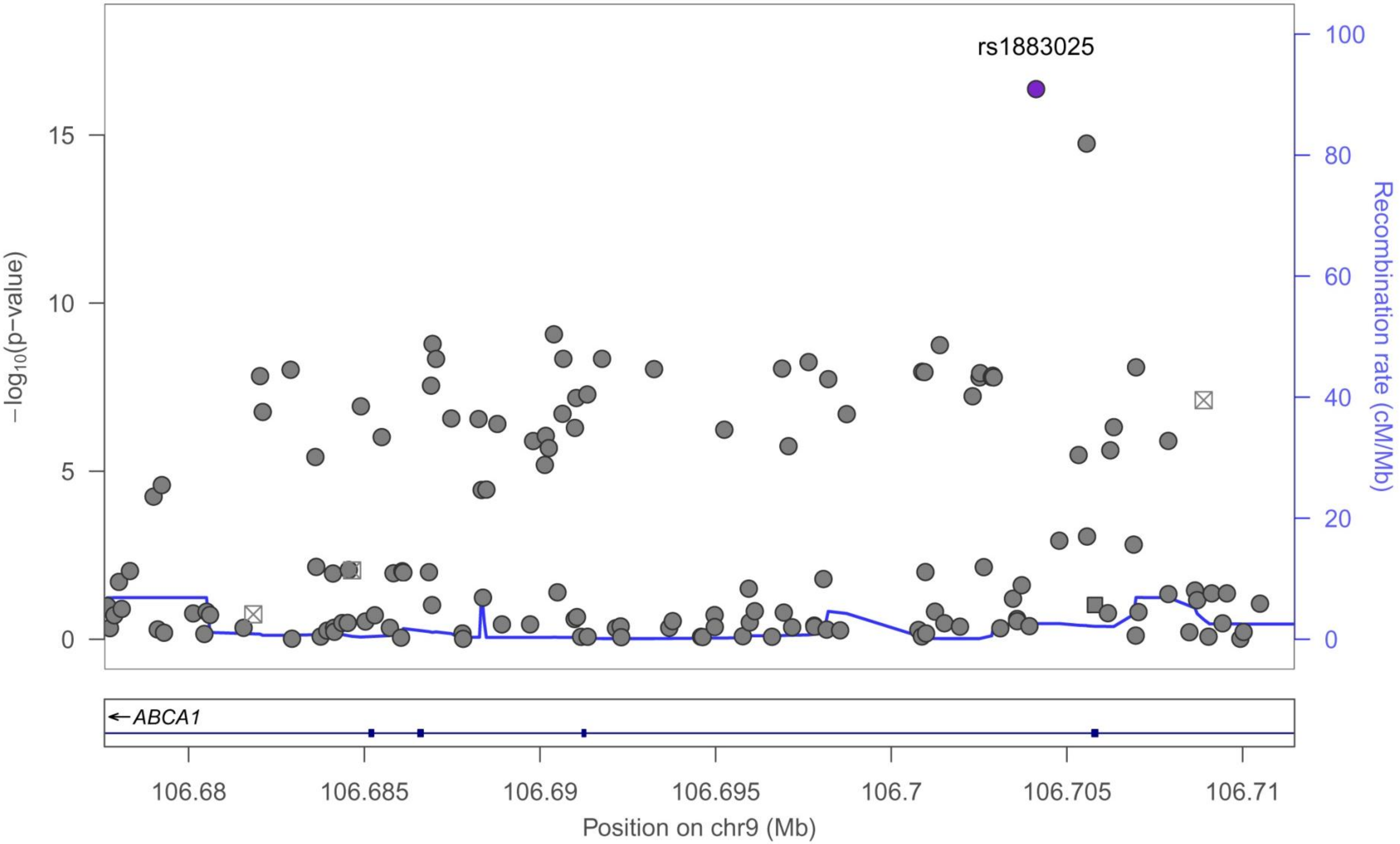


**Figure S5D.** Association at HDL-C locus *ABCA1* in trans-ethnic meta-analysis.
