# Supplementary material for: Trans-Ethnic Fine-Mapping of Lipid Loci Identifies Population-Specific Signals and Allelic Heterogeneity That Increases the Trait Variance Explained
Source: PLoS Genet. 2013 Mar 21;9(3):e1003379. doi: 10.1371/journal.pgen.1003379 (PMC3605054; doi:10.1371/journal.pgen.1003379)
Supplement: Figure S6 — Association at TG locus APOA5 in Europeans (A), East Asians (B), African Americans (C), and trans-ethnic meta-analysis (D). The SNPs rs3741298, rs651821 (-3A>G), rs3135506 (S19W), and rs662799 that exhibited the smallest P values in Europeans, East Asians, African Americans, and the trans-ethnic meta-analysis are indicated. (PDF) [file pgen.1003379.s006.pdf]

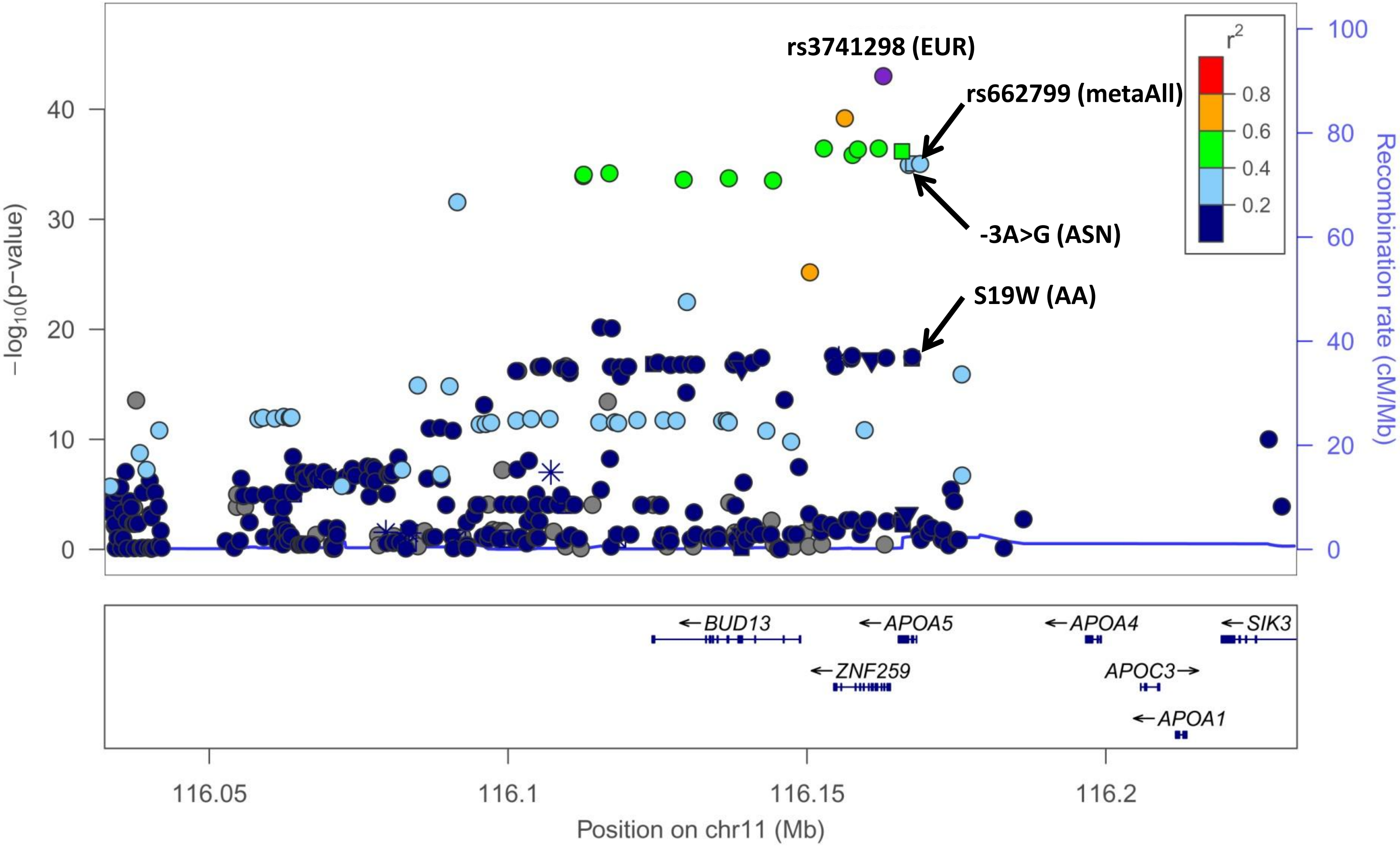

FigureS6A. Association at TG locus *APOA5* in Europeans. Index SNP rs3741298 is the variant showing the strongest evidence of association in Europeans. The SNPs with the smallest *P* values in East Asians (-3A>G), African Americans (S19W) and the trans-ethnic meta-analysis (rs662799) are also indicated.

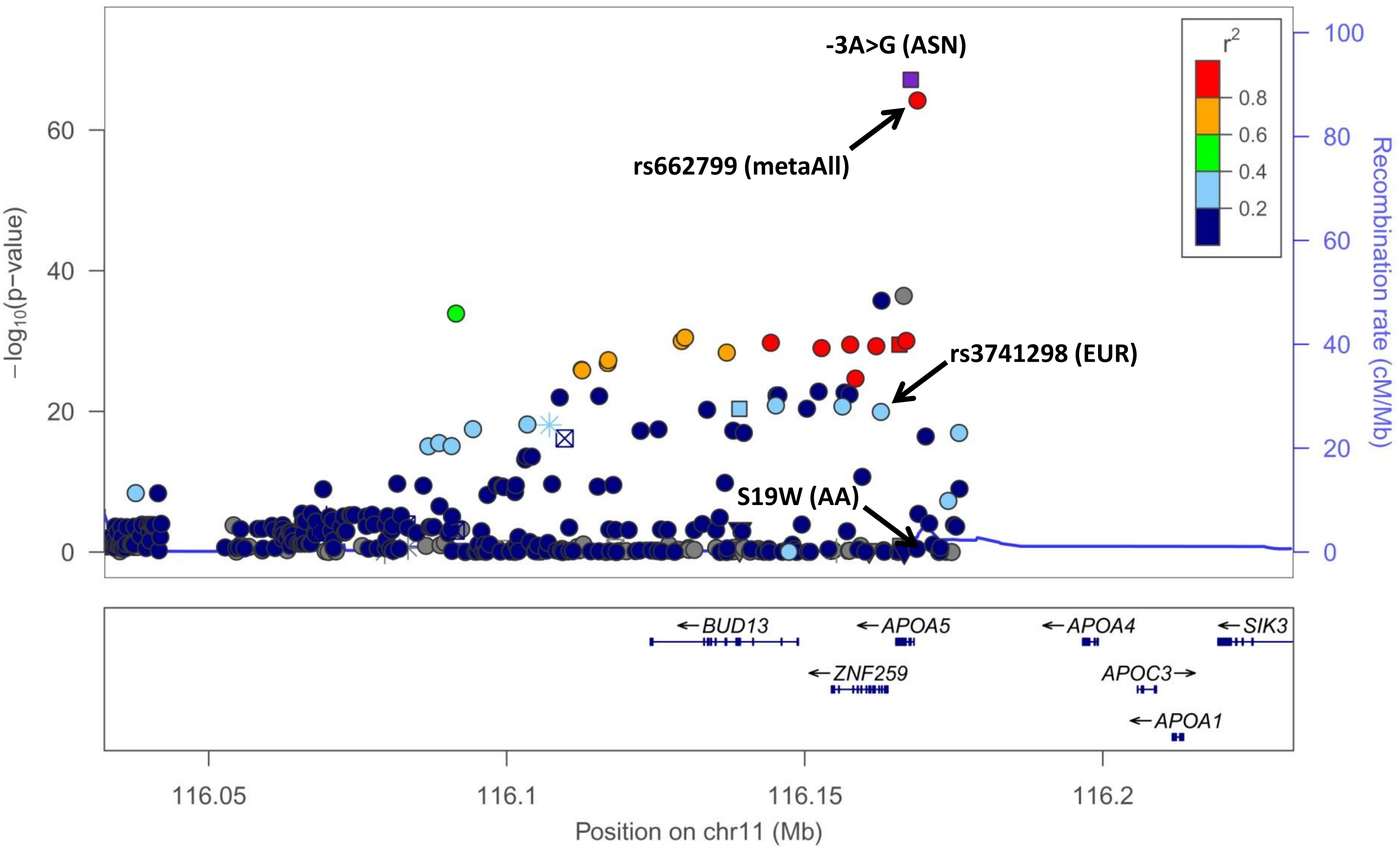

FigureS6B. Association at TG locus *APOA5* in East Asians. Index SNP -3A>G is the variant showing the strongest evidence of association in East Asians. The SNPs with the smallest *P* values in Europeans (rs3741298), African Americans (S19W) and the trans-ethnic meta-analysis (rs662799) are also indicated.

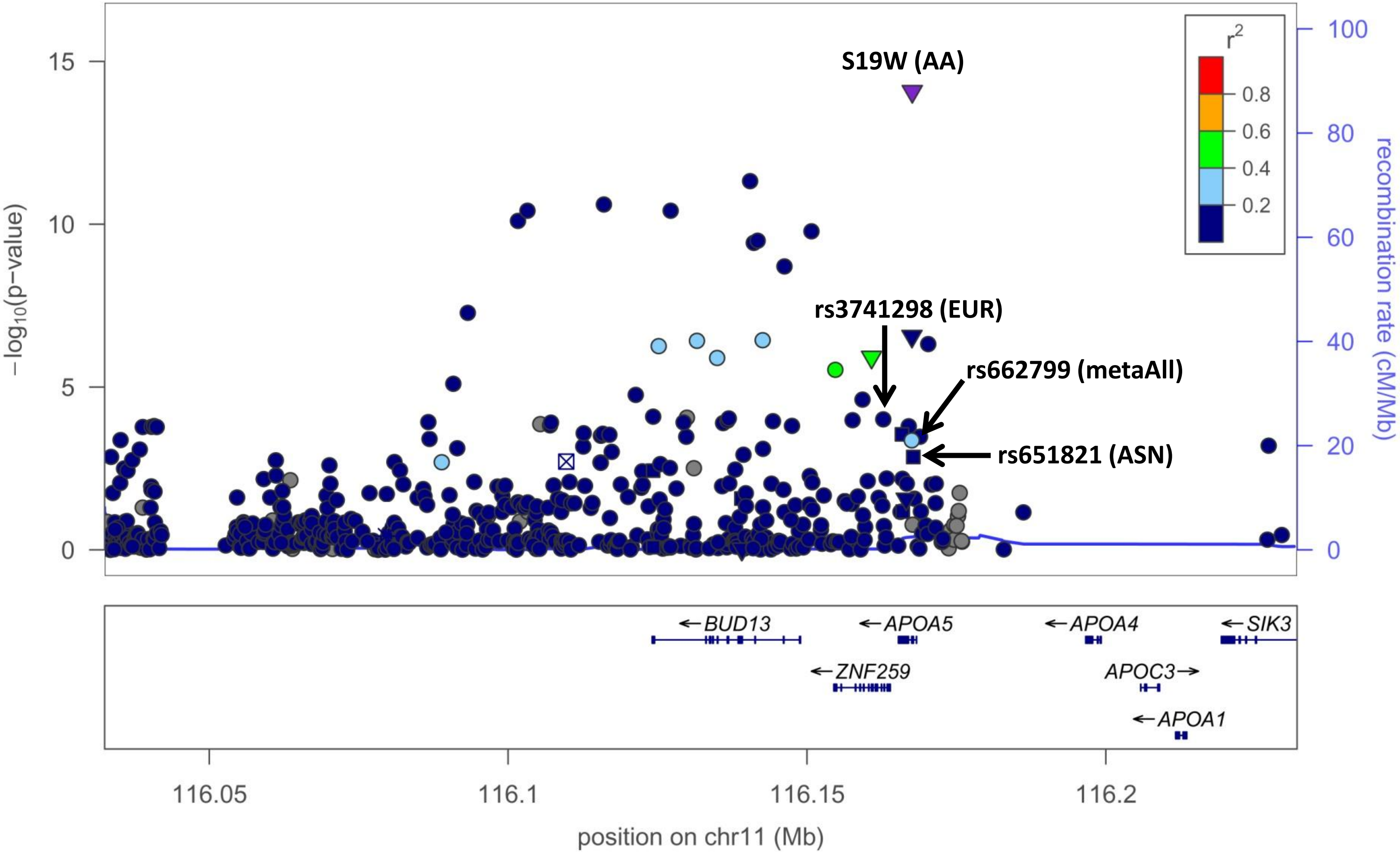

FigureS6C. Association at TG locus *APOA5* in African Americans. Index SNP S19W is the variant showing the strongest evidence of association in African Americans. The SNPs with the smallest *P* values in Europeans (rs3741298), East Asians (-3A>G) and the trans-ethnic meta-analysis (rs662799) are also indicated.

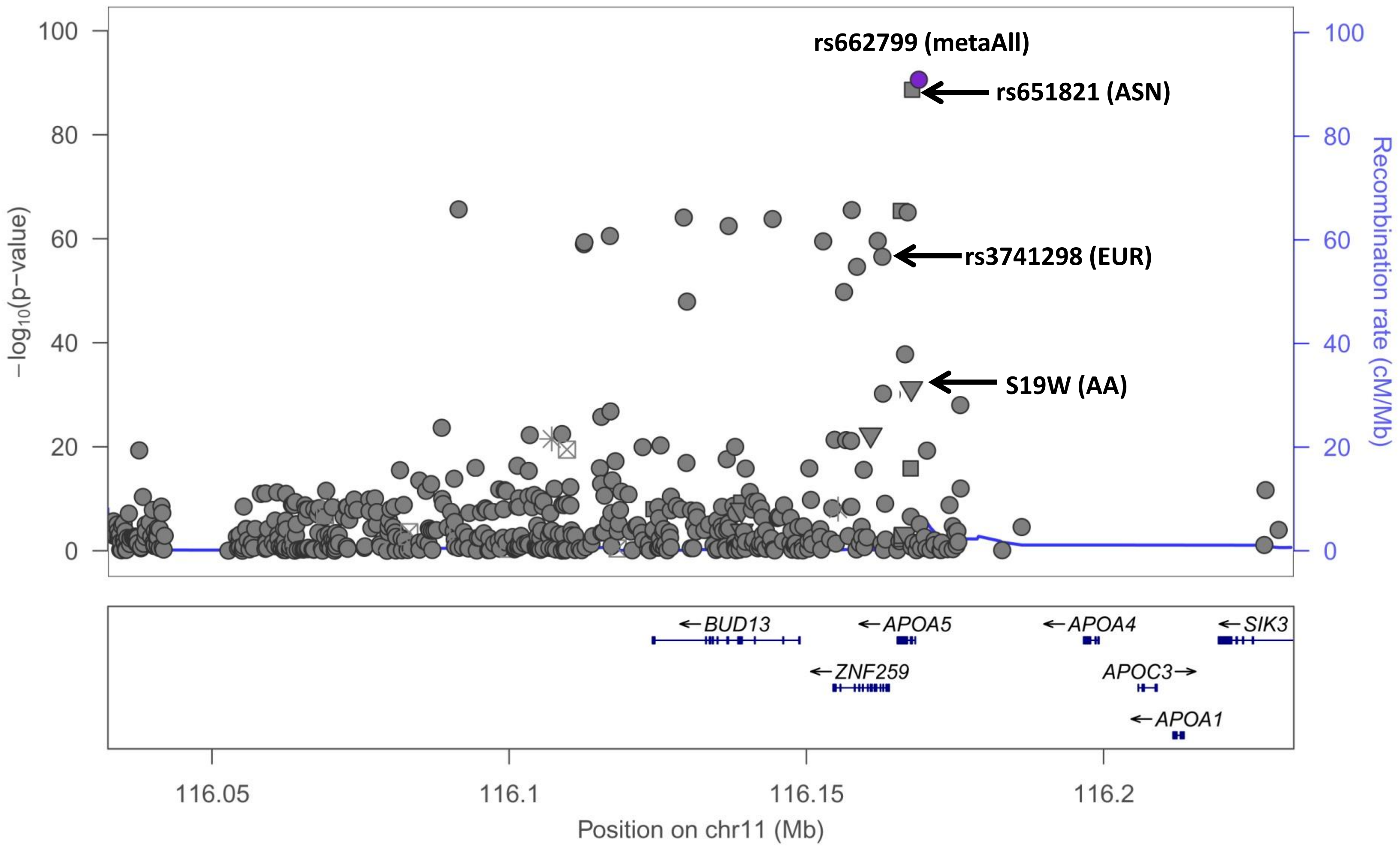

FigureS6D. Association at TG locus *APOA5* in the trans-ethnic meta-analysis. Index SNP rs662799 is the variant showing the strongest evidence of association in trans-ethnic meta-analysis. The SNPs with the smallest *P* values in Europeans (rs3741298), East Asians (-3A>G) and African Americans (S19W) are also indicated.
