# Supplementary material for: Trans-Ethnic Fine-Mapping of Lipid Loci Identifies Population-Specific Signals and Allelic Heterogeneity That Increases the Trait Variance Explained
Source: PLoS Genet. 2013 Mar 21;9(3):e1003379. doi: 10.1371/journal.pgen.1003379 (PMC3605054; doi:10.1371/journal.pgen.1003379)
Supplement: Table S1 — Characteristics of the study samples. (PDF) [file pgen.1003379.s007.pdf]

Table S1. Characteristics of the study samples

A. Study Characteristics											
Study	ARIC	MEC	WHI Batch1	WHI Batch2	HyperGEN	CLHNS	TAICHI	Finnish T2D patients [a]	Finnish unaffected [a]	Norwegian T2D [b]	Norwegian unaffected [b]
Study design	population-based	population-based	population-based	population-based	family-based	population-based	population-based	Selected T2D cases from population-based studies	Selected controls from population-based studies	Selected T2D cases from population-based studies	Selected controls from population-based studies
Ethnicity of study participants	African American	African American	African American	African American	African American	East Asian	East Asian	European	European	European	European
B. Phenotypes											
Total sample size (% female)	3,180 (62.4)	380 (17.1)	1,387 (100)	646 (100)	1,230 (66.9)	1,716 (47.6)	7,733 (38.7)	1,970 (30.7)	6,066 (45.3)	1,298 (50.0)	1,495 (49.6)
Age (mean±SD, yrs)	53.6±5.9	---- [c]	61.7±7.2	60.7±6.6	47.0±12.6	21.5±0.3	65.46 ± 11.44	60.3 ± 8.1	58.4 ± 8.4	64.2 ± 12.8	63.3 ± 13.9
Fasting status	≥ 8 hours	≥ 8 hours	≥ 8 hours	≥ 8 hours	≥ 8 hours	≥ 8 hours	various	overnight or >8 hours fast	overnight or >8 hours fast	non-fasting	non-fasting
Use of lipid-lowering medication & exclusion	NO exclusion [d]	NO exclusion [d]	NO exclusion [d]	NO exclusion [d]	NO exclusion [d]	NO exclusion [d]	NO exclusion [d]	Exclude when known to be on lipid-lowering medication	Exclude when known to be on lipid-lowering medication	NO relevant information available	NO relevant information available
Methods/reagents of triglycerides measurement	Standard enzymatic methods	Standard enzymatic methods	Standard enzymatic methods	Standard enzymatic methods	Standard enzymatic methods	Standard enzymatic methods	varied across study sites	Standard enzymatic methods	Standard enzymatic methods	Standard enzymatic methods	Standard enzymatic methods
Methods/reagents of HDL-C measurement	Standard enzymatic methods	Standard enzymatic methods	Standard enzymatic methods	Standard enzymatic methods	Standard enzymatic methods	Homogenous assay direct HDL-C (Equal Diagnostics, Exton, PA)	varied across study sites	Standard enzymatic methods	Standard enzymatic methods	Standard enzymatic methods	Standard enzymatic methods
Methods/reagents of LDL-C measurement	Calculated using Friedewald equation	Calculated using Friedewald equation	Calculated using Friedewald equation	Calculated using Friedewald equation	Calculated using Friedewald equation	Homogenous assay direct LDL-C (Equal Diagnostics, Exton, PA)	varied across study sites	Calculated using Friedewald equation	Calculated using Friedewald equation	Calculated using Friedewald equation	Calculated using Friedewald equation
Triglycerides (median [interquartile	1.10 (0.81-1.51)	1.30 (0.82-1.53)	1.35 (0.93-1.59)	1.08 (0.81-1.46)	1.04 (0.75-1.50)	1.19 (0.74-1.41)	1.33 (0.94 - 1.95)	1.83 (1.14-2.17)	1.33 (0.87-1.56)	2.60 (1.53-3.19)	1.87 (1.13-2.34)
HDL-C (mean±SD, mmol/L)	1.42 ± 0.45	1.37 ± 0.38	1.51 ± 0.39	1.47 ± 0.38	1.38 ± 0.41	1.09 ± 0.29	1.21 ± 0.36	1.33 ± 0.39	1.52 ± 0.41	1.26 ± 0.39	1.42 ± 0.41
LDL-C (mean±SD, mmol/L)	3.57 ± 1.12	3.20 ± 1.13	3.40 ± 0.99	3.69 ± 1.02	3.08 ± 0.97	2.44 ± 0.75	2.93 ± 0.91	3.32 ± 0.94	3.51 ± 0.84	4.16 ± 1.11	4.27 ± 1.14
C. Genotyping											
Metabochip genotype calling	GenCall	GenCall	GenCall	GenCall	BeadStudio	BeadStudio	BeadStudio	GenomeStudio	GenomeStudio	GenomeStudio	GenomeStudio
HWE P value threshold	> 1E-06	> 1E-06	> 1E-06	> 1E-06	> 1E-06	> 1E-06	> 0.001	n.a.	n.a.	n.a.	n.a.
SNP call rate	95%	95%	95%	95%	95%	97%	95%	95%	95%	95%	95%
SNP MAF threshold	n.a.	n.a.	n.a.	n.a.	n.a.	n.a.	n.a.	n.a.	n.a.	n.a.	n.a.
Other SNP QC	Mendelian errors in YRI > 1 (out of 30 trios); Discordant calls on YRI > 3 (out of 90 samples); Discordant calls from GenoSNP > 3.3%; PAGE consensus vs HapMap database > 3 (out of 90 samples)	Mendelian errors in YRI > 1 (out of 30 trios); Discordant calls on YRI > 3 (out of 90 samples); Discordant calls from GenoSNP > 3.3%; PAGE consensus vs HapMap database > 3 (out of 90 samples)	Mendelian errors in YRI > 1 (out of 30 trios); Discordant calls on YRI > 3 (out of 90 samples); Discordant calls from GenoSNP > 3.3%; PAGE consensus vs HapMap database > 3 (out of 90 samples)	Mendelian errors in YRI > 1 (out of 30 trios); Discordant calls on YRI > 3 (out of 90 samples); Discordant calls from GenoSNP > 3.3%; PAGE consensus vs HapMap database > 3 (out of 90 samples)	GenTrain score <0.7; Cluster Separation Score < 0.45 (both GenTrain and CSS were selected at 5% percentile)	poor genotyping cluster; MI errors in ⩾ 3 samples; SNPs with ⩾ 3 discrepancies with known HapMap genotypes among 14 CEU samples; SNPs with primers that do not map > 97% to genome or map to multiple places in genome	replication errors (1 or more)	Cluster Separation score less than 0.2 or which had more than 1 replicate error as defined with the HapMap control samples. Additional hand editing was done for X, Y and Mitochondrial loci.	Cluster Separation score less than 0.2 or which had more than 1 replicate error as defined with the HapMap control samples. Additional hand editing was done for X, Y and Mitochondrial loci.	Cluster Separation score less than 0.2 or which had more than 1 replicate error as defined with the HapMap control samples. Additional hand editing was done for X, Y and Mitochondrial loci.	Cluster Separation score less than 0.2 or which had more than 1 replicate error as defined with the HapMap control samples. Additional hand editing was done for X, Y and Mitochondrial loci.
Sample success rate	≥98.98%	≥97.74%	≥97.70%	≥97.70%	≥98.5%	≥ 98.6%	⩾ 98.49%	⩾ 98.15%	⩾ 98.15%	⩾ 98.15%	⩾ 98.15%
Concordance rate for duplicate pairs	99.51%	99.99%	99.99%	99.99%	99.98%	99.99%	----	Across all our Metabochip samples, 99.9978% reproducibility among 163 blind duplicate pairs; 99.52% concordance with HapMap genotypes (326 HapMap controls)	Across all our Metabochip samples, 99.9978% reproducibility among 163 blind duplicate pairs; 99.52% concordance with HapMap genotypes (326 HapMap controls)	Across all our Metabochip samples, 99.9978% reproducibility among 163 blind duplicate pairs; 99.52% concordance with HapMap genotypes (326 HapMap controls)	Across all our Metabochip samples, 99.9978% reproducibility among 163 blind duplicate pairs; 99.52% concordance with HapMap genotypes (326 HapMap controls)
D. Statistical analysis											
Software for association analyses	PLINK	PLINK	PLINK	PLINK	GWAF	PLINK	PLINK	EMMAX	EMMAX	EMMAX	EMMAX
Statistical model	multiple linear	multiple linear	multiple linear	multiple linear	linear mixed effect	multiple linear	multiple linear	mixed model	mixed model	mixed model	mixed model
Covariates used	age, sex, PC1-10	age, sex, PC1-10	age, PC1-10	age, PC1-10	age, sex, PC1-10	age, sex, household assests, household income, PC1	age, sex, PC1-PC5, study cohort	sex, age, age^2, study	sex, age, age^2, study	sex, age, age^2, study	sex, age, age^2, study

[a] Finnish T2D patients and unaffected individuals were from the Finland-United States Investigation of NIDDM Genetics (FUSION), Dehko 2D 2007 (D2D2007), Diabetes Prevention Study (DPS), Dose-Responses to Exercise Training (DR's EXTRA), and Metabolic Syndrome in Men (METSIM)

[b] Norwegian T2D patients and unaffected individuals were from the cohorts of Nord-Trøndelag Health Study (HUNT 2) and the Tromsø Study (TROMSO)

[c] The mean and SD were not provided for MEC because the ages of MEC subjects were provided as a categorical value with seven 5-year age groups due to confidentiality

[d] Sensitivity analyses that excluded individuals on lipid lowering medication did not appreciably alter the association results
