# Supplementary material for: Trans-Ethnic Fine-Mapping of Lipid Loci Identifies Population-Specific Signals and Allelic Heterogeneity That Increases the Trait Variance Explained
Source: PLoS Genet. 2013 Mar 21;9(3):e1003379. doi: 10.1371/journal.pgen.1003379 (PMC3605054; doi:10.1371/journal.pgen.1003379)
Supplement: Table S2 — Number of SNPs at each locus for analysis in each of the three ancestry groups. (PDF) [file pgen.1003379.s008.pdf]

Table S2. Number of SNPs at each locus for analysis in each of the three ancestry groups

Locus	Chr	Pos_start	Pos_end	Length (kb)	Trait	African American	East Asian	European
ANGPTL3	1	62605319	63131404	526.1	TG	825	480	474
APOB	2	20968276	21115932	147.7	TG	485	249	271
GCKR	2	27240303	28524485	1284.2	TG	2220	1060	1300
KLHL8	4	88199054	88388945	189.9	TG	415	248	286
MAP3K1	5	55876150	55917936	41.8	TG	116	80	80
TYW1B	7	71646192	71931044	284.9	TG	279	109	118
MLXIPL	7	72339015	72771177	432.2	TG	585	266	325
PINX1	8	10619933	10769460	149.5	TG	529	393	386
NAT2	8	18278054	18335870	57.8	TG	294	195	212
LPL	8	19772866	20011766	238.9	TG	1067	633	662
TRIB1	8	126533689	126579084	45.4	TG	191	116	126
APOA5	11	116032491	116232000	199.5	TG	543	370	398
LRP1	12	55910892	56137449	226.6	TG	103	50	54
KLF12	13	73456517	73492535	36.0	TG	99	68	72
CAPN3	15	40402264	40539209	136.9	TG	213	155	173
LIPC	15	56450000	56550000	100.0	TG	178	126	133
CETP	16	55540320	55565013	24.7	TG	100	65	72
APOC1	19	50059760	50164446	104.7	TG	109	69	79
GALNT2	1	228268651	228485397	216.7	HDL-C	694	502	536
APOB	2	20968276	21115932	147.7	HDL-C	485	249	271
COBLL1	2	165207794	165440664	232.9	HDL-C	456	297	314
PPP1R3B	8	9205275	9422842	217.6	HDL-C	888	630	666
LPL	8	19772866	20011766	238.9	HDL-C	1067	633	662
TTC39B	9	15272851	15326653	53.8	HDL-C	172	131	137
ABCA1	9	106677603	106711480	33.9	HDL-C	149	75	100
ARFGAP2	11	46654261	47310074	655.8	HDL-C	894	497	554
FADS3	11	61276118	61489925	213.8	HDL-C	551	339	336
APOA5	11	116032491	116175948	143.5	HDL-C	543	370	390
MMAB	12	108256738	108565110	308.4	HDL-C	788	600	621
SCARB1	12	123820589	123834385	13.8	HDL-C	43	30	32
LIPC	15	56450000	56550000	100.0	HDL-C	178	126	133
LACTB	15	61106592	61346662	240.1	HDL-C	454	296	319
CETP	16	55540320	55565013	24.7	HDL-C	100	65	72
LCAT	16	66109606	66883748	774.1	HDL-C	814	452	514
STARD3	17	34640966	35328044	687.1	HDL-C	842	473	500
ABCA8	17	64325421	64414888	89.5	HDL-C	181	138	132
LIPG	18	45378906	45436862	58.0	HDL-C	303	236	226
LILRA3	19	59464550	59502182	37.6	HDL-C	142	92	94
HNF4A	20	42472196	42491494	19.3	HDL-C	49	35	38
PLTP	20	43965592	44095820	130.2	HDL-C	310	176	197
LDLRAP1	1	25398494	25780828	382.3	LDL-C	535	178	255
PCSK9	1	55271537	55310000	38.5	LDL-C	96	57	59
SORT1	1	109457160	109845216	388.1	LDL-C	760	453	448
APOB	2	21080065	21305332	225.3	LDL-C	858	503	527
ABCG8	2	43910534	43954353	43.8	LDL-C	172	72	106
HMGCR	5	74603868	74991808	387.9	LDL-C	598	383	405
TIMD4	5	156263672	156437811	174.1	LDL-C	474	247	265
MYLIP	6	16212233	16242816	30.6	LDL-C	68	50	52
SLC22A1	6	160388268	160499517	111.2	LDL-C	284	186	189
TRIB1	8	126510832	126613110	102.3	LDL-C	368	213	238
ABO	9	135032145	135472297	440.2	LDL-C	1181	931	823
ST3GAL4	11	125724639	125784557	59.9	LDL-C	167	107	92
DHODH	16	70553792	70705184	151.4	LDL-C	402	277	282
LDLR	19	11044837	11072208	27.4	LDL-C	60	35	32
CILP2	19	19162232	19653250	491.0	LDL-C	946	591	606
APOE	19	50088739	50136106	47.4	LDL-C	72	41	47
MAFB	20	38516556	38561992	45.4	LDL-C	92	49	56
TOP1	20	39047398	39443459	396.1	LDL-C	556	311	346
Total:						**26143**	**15858**	**16883**
Mean:						**451**	**273**	**291**
