# Supplementary material for: Trans-Ethnic Fine-Mapping of Lipid Loci Identifies Population-Specific Signals and Allelic Heterogeneity That Increases the Trait Variance Explained
Source: PLoS Genet. 2013 Mar 21;9(3):e1003379. doi: 10.1371/journal.pgen.1003379 (PMC3605054; doi:10.1371/journal.pgen.1003379)
Supplement: Table S3 — Lead SNP at TG (A), HDL-C (B), and LDL-C (C) loci within each ancestry group and their relative significance compared to reported GWAS index SNPs. (PDF) [file pgen.1003379.s009.pdf]

Table S3A. Lead SNP at TG loci within each ancestry group and their relative significance compared to reported GWAS index SNPs

TG		African American				East Asian				European			
Locus		**Index SNP**	**MAF**	***P***	**LD (r^2/D')[a]**	**Index SNP**	**MAF**	***P***	**LD (r^2/D')[b]**	**Index SNP**	**MAF**	***P***	**LD (r^2/D')[c]**
APOA5	GWAS[d]	rs12286037	0.191	4.00E-01	0.04/0.36	rs12286037	0.008	6.60E-01	n.a.	rs12286037	0.058	4.55E-18	0.23/1.00
	Metabochip	rs3135506 (S19W)	0.058	8.36E-15		rs651821 (-3A>G)	0.275	7.21E-68		rs3741298	0.222	9.68E-44	
GCKR	GWAS	rs1260326	0.149	2.20E-08	same	rs1260326	0.484	1.54E-13	0.42/0.93	rs1260326	0.350	6.04E-24	same
	Metabochip	rs1260326 (L446P)	0.149	2.20E-08		rs814295	0.350	1.52E-13		rs1260326 (L446P)	0.350	4.40E-24	
LPL	GWAS	rs12678919	0.112	1.94E-03	0.29/0.60	rs12678919	0.094	1.25E-10	0.86/1.00	rs12678919	0.088	2.63E-11	0.31/1.00
	Metabochip	rs75551077	0.135	1.31E-09		rs1803924	0.095	1.11E-11		rs15285 (3'UTR)	0.258	1.08E-16	
TRIB1	GWAS	rs2954029	0.350	7.52E-01	0.05/0.94	rs2954029	0.452	1.74E-03	0.56/1.00	rs2954029	0.465	1.54E-16	same
	Metabochip	rs2980858	0.094	3.12E-06		rs2954018	0.290	1.70E-05		rs2954029	0.465	1.54E-16	
APOC1	GWAS	rs439401	0.155	2.06E-01	0.03/0.99	rs439401	0.430	4.02E-04	0.06/0.46	rs439401	0.301	1.82E-04	0.02/0.51
	Metabochip	rs12721054 (3'UTR)	0.119	3.57E-19		rs7259004	0.244	4.11E-05		rs445925	0.084	1.38E-04	
MLXIPL	GWAS	rs17145738	0.091	8.29E-04	0.99/1.00	rs17145738	0.099	1.85E-08	0.68/0.92	rs17145738	0.127	3.17E-05	0.32/0.94
	Metabochip	rs13246490	0.091	6.77E-04		rs67566641	0.088	7.75E-11		rs7808877	0.245	2.55E-08	
ANGPTL3	GWAS	rs2131925	0.348	1.00E-01	0.06/0.94	rs2131925	0.259	8.06E-04	0.89/1.00	rs2131925	0.281	1.41E-11	0.98/0.99
	Metabochip	rs7523145	0.121	7.19E-05		rs1748199	0.223	2.86E-05		rs1748199	0.281	9.87E-12	
APOB	GWAS	rs1042034	0.157	4.81E-06	1.00/1.00	rs1042034	0.276	7.48E-01	0.27/1.00	rs1042034	0.260	1.64E-10	0.99/0.99
	Metabochip	rs676210 (P2739L)	0.157	3.97E-06		rs13306194	0.149	9.87E-05		rs2678379	0.260	1.50E-10	
LIPC	GWAS	rs1532085	0.481	3.07E-02	0.01/0.84	rs1532085	0.465	1.69E-01	0.44/1.00	rs1532085	0.423	5.18E-05	0.64/0.98
	Metabochip	rs115573508	0.012	1.37E-03		rs7164909	0.348	9.87E-03		rs10468017	0.328	2.99E-06	
CETP	GWAS	rs3764261	0.319	5.26E-01	0.00/0.11	rs3764261	0.166	1.53E-01	0.00/0.25	rs3764261	0.282	3.66E-02	0.10/0.96
	Metabochip	rs186526203	0.144	3.90E-03		rs79374262	0.016	1.46E-02		rs11076175	0.175	1.01E-04	
NAT2	GWAS	rs1495741	n.a.	n.a.	n.a.	rs1495741	n.a.	n.a.	0.71/0.96	rs1495741	n.a.	n.a.	0.13/0.96
	Metabochip	rs78728596	0.145	1.24E-03		rs4921911	0.424	7.36E-04		rs1041983 (Y94Y)	0.282	2.39E-03	
KLHL8	GWAS	rs442177	0.499	6.31E-01	0.00/0.63	rs442177	0.421	4.58E-02	0.22/0.69	rs442177	0.261	6.09E-02	0.09/0.48
	Metabochip	rs113071385	0.009	5.00E-04		rs2061498	0.345	7.55E-04		rs4134363	0.221	6.26E-04	
PINX1	GWAS	rs11776767	0.351	2.87E-01	0.03/0.42	rs11776767	0.224	6.50E-01	0.01/0.97	rs11776767	0.313	1.27E-01	0.47/0.89
	Metabochip	rs17152623	0.253	5.56E-04		rs118016348	0.009	1.60E-02		rs7014285	0.199	1.06E-02	
TYW1B	GWAS	rs13238203	0.007	6.27E-01	0.00/1.00	rs13238203	n.a.	n.a.	n.a.	rs13238203	n.a.	n.a.	1.00/0.00
	Metabochip	rs114204448	0.020	8.95E-04		rs1892751	0.357	5.60E-02		rs71553242	0.025	1.64E-02	
LRP1	GWAS	rs11613352	0.095	3.00E-01	0.01/1.00	rs11613352	0.091	8.91E-01	0.00/1.00	rs11613352	0.247	5.87E-01	0.02/1.00
	Metabochip	rs10735872	0.087	1.83E-03		rs117312963	0.001	1.32E-01		rs11172224	0.008	3.61E-02	
MAP3K1	GWAS	rs9686661	0.239	3.40E-01	0.07/1.00	rs9686661	0.114	2.66E-01	0.06/1.00	rs9686661	0.137	4.11E-03	0.93/0.97
	Metabochip	rs74762860	0.184	6.32E-02		rs6450394	0.336	4.68E-03		rs3936510	0.143	1.40E-03	
CAPN3	GWAS	rs2412710	0.074	8.74E-01	0.00/0.18	rs2412710	n.a.	9.38E-01	0.00/0.15	rs2412710	0.013	8.00E-02	0.00/1.00
	Metabochip	rs1801449 (A149S)	0.374	2.75E-03		rs3742996	0.128	1.22E-02		rs118166665	0.010	4.44E-02	
KLF12	GWAS	rs9592961	0.018	4.29E-01	0.00/1.00	rs9592961	0.061	7.85E-01	0.00/0.40	rs9592961	0.006	9.66E-01	0.00/1.00
	Metabochip	rs9543507	0.113	2.06E-02		rs7328112	0.104	5.40E-02		rs114713792	0.007	1.52E-01	

[a] LD (r^2/D') estimates were calculated from the genotype data of the PAGE African American samples, whose LD patterns may vary from any LD sources in 1000 Genomes Project

[b] LD (r^2/D') estimates were from the 1000 Genomes Project ASN samples

[c] LD (r^2/D') estimates were from the 1000 Genomes Project EUR samples

[d] Results of association with reported GWAS index SNPs were based on current study samples

n.a. the GWAS index SNP was not genotyped by Metabochip or was excluded in quality control, or the pairwise LD estimates are not applicable

Table S3B. Lead SNP at HDL-C loci within each ancestry group and their relative significance compared to reported GWAS index SNPs

HDL-C		African American				East Asian				European			
Locus		**Index SNP**	**MAF**	***P***	**LD (r^2/D')[a]**	**Index SNP**	**MAF**	***P***	**LD (r^2/D')[b]**	**Index SNP**	**MAF**	***P***	**LD (r^2/D')[c]**
CETP	GWAS[d]	rs3764261	0.319	8.78E-27	0.74/0.99	rs3764261	0.168	1.24E-27	1.00/1.00	rs3764261	0.285	2.21E-58	0.99/0.99
	Metabochip	rs247617	0.259	1.09E-42		rs17231506 (5'UTR)	0.168	3.63E-28		rs56156922	0.284	4.68E-59	
LIPC	GWAS	rs1532085	0.481	1.27E-01	0.00/0.02	rs1532085	0.474	2.87E-10	0.04/0.21	rs1532085	0.422	6.30E-19	0.59/1.00
	Metabochip	rs1077834	0.481	2.22E-06		rs588136	0.371	3.67E-14		rs10468017	0.327	2.53E-21	
LPL	GWAS	rs12678919	0.113	4.13E-03	0.10/0.74	rs12678919	0.091	6.51E-06	1.00/1.00	rs12678919	0.088	5.33E-06	0.39/1.00
	Metabochip	rs10096633	0.421	1.01E-12		rs79236614	0.091	2.18E-06		rs15285 (3'UTR)	0.259	1.44E-09	
ABCA1	GWAS	rs1883025	0.336	1.77E-02	same	rs1883025	0.271	3.74E-11	same	rs1883025	0.209	4.53E-07	same
	Metabochip	rs1883025	0.336	1.77E-02		rs1883025	0.271	3.74E-11		rs1883025	0.209	4.53E-07	
APOA5	GWAS	rs12286037	0.191	5.20E-01	0.00/0.13	rs12286037	0.008	4.00E-01	0.00/1.00 [e]	rs12286037	0.058	4.50E-05	0.23/1.00
	Metabochip	rs189069311	0.043	4.16E-05		rs651821 (-3A>G)	0.274	4.41E-11		rs3741298	0.224	1.98E-05	
LCAT	GWAS	rs16942887	0.214	3.47E-06	0.87/0.93	rs16942887	0.029	2.44E-01	0.57/0.82	rs16942887	0.152	2.21E-04	0.79/1.00
	Metabochip	rs255054	0.211	3.24E-07		rs12447119 (5'UTR)	0.077	2.00E-04		rs1109166	0.192	5.00E-05	
APOB	GWAS	rs1042034	0.158	7.47E-03	0.88/0.95	rs1042034	0.282	3.63E-03	0.19/1.00	rs1042034	0.258	4.95E-08	1.00/1.00
	Metabochip	rs10184054	0.158	3.11E-03		rs56984418	0.073	7.81E-05		rs2678379	0.258	4.28E-08	
LIPG	GWAS	rs4939883	0.453	4.79E-01	0.04/0.57	rs4939883	0.177	5.89E-05	0.47/1.00	rs4939883	0.183	5.42E-05	0.64/1.00
	Metabochip	rs1016563	0.141	1.80E-04		rs1943973	0.178	1.90E-06		rs7244811	0.283	1.38E-05	
PPP1R3B	GWAS	rs9987289	0.184	2.48E-04	0.57/0.96	rs9987289	0.056	2.39E-01	0.24/0.67	rs9987289	n.a.	n.a.	0.40/0.74
	Metabochip	rs6601299	0.121	7.99E-08		rs7357361	0.032	2.55E-03		rs114798927	0.151	7.93E-05	
SCARB1	GWAS	rs838880	0.325	4.29E-01	0.01/1.00	rs838880	0.478	1.61E-03	0.22/0.74	rs838880	0.407	5.68E-05	0.83/0.95
	Metabochip	rs116354593	0.016	9.20E-03		rs7977729	0.342	3.61E-04		rs838876	0.401	2.09E-05	
ARFGAP2	GWAS	rs3136441	0.034	7.31E-01	0.00/0.45	rs3136441	0.438	6.14E-01	0.02/0.65	rs3136441	0.219	1.14E-05	0.13/0.61
	Metabochip	rs901750	0.204	5.72E-04		rs4647756	0.058	2.09E-03		rs1055510	0.402	1.27E-06	
GALNT2	GWAS	rs4846914	0.147	1.30E-01	0.00/0.31	rs4846914	0.248	4.91E-02	0.02/0.19	rs4846914	0.443	5.28E-04	1.00/1.00
	Metabochip	rs60506249	0.122	9.47E-03		rs78493600	0.100	4.99E-03		rs17315646	0.443	4.78E-04	
PLTP	GWAS	rs6065906	0.162	1.90E-02	0.26/0.93	rs6065906	0.027	5.01E-01	0.00/0.15	rs6065906	0.169	6.55E-03	n.a.
	Metabochip	rs4810479	0.397	9.50E-05		rs8114050	0.047	9.53E-04		rs6017720	0.156	3.74E-03	
LILRA3	GWAS	rs386000	0.299	4.83E-01	0.00/0.98	rs386000	0.388	5.21E-01	0.03/0.20	rs386000	0.304	1.31E-04	n.a.
	Metabochip	rs73055464	0.006	8.34E-04		rs434124	0.026	3.06E-02		rs798893	0.239	8.83E-05	
MMAB	GWAS	rs9943753	0.080	8.70E-01	0.00/0.02	rs9943753	0.441	6.47E-02	0.65/1.00	rs9943753	0.428	3.94E-01	0.10/1.00
	Metabochip	rs111273776	0.062	2.33E-03		rs3815575	0.257	6.40E-04		rs2287182	0.051	9.68E-03	
FADS3	GWAS	rs174546	0.084	1.25E-01	0.01/0.65	rs174546	0.384	3.68E-02	0.15/0.54	rs174546	0.408	2.75E-02	0.25/0.90
	Metabochip	rs11230827	0.196	4.18E-04		rs1534842	0.336	3.09E-03		rs174602	0.203	4.49E-03	
COBLL1	GWAS	rs12328675	0.159	4.03E-01	0.00/0.76	rs12328675	0.003	9.91E-01	0.00/1.00	rs12328675	0.097	9.96E-04	n.a.
	Metabochip	rs58133528	0.025	1.83E-02		rs34309702	0.022	1.09E-01		rs190303539	0.349	8.47E-05	
TTC39B	GWAS	rs581080	0.498	3.55E-01	0.07/0.95	rs581080	0.107	5.38E-01	0.00/1.00	rs581080	0.150	8.30E-02	0.71/1.00
	Metabochip	rs189682870	0.072	1.39E-03		rs117901166	0.005	1.01E-02		rs686030	0.122	1.69E-03	
STARD3	GWAS	rs11869286	0.260	6.86E-01	0.03/0.93	rs11869286	0.368	5.52E-02	0.12/0.69	rs11869286	0.314	3.31E-01	0.01/1.00
	Metabochip	rs78739040	0.076	3.47E-03		rs12150079	0.183	7.92E-03		rs117064469	0.007	7.78E-03	
LACTB	GWAS	rs2652834	0.289	8.00E-01	0.07/0.93	rs2652834	0.011	7.10E-01	0.00/0.02	rs2652834	0.210	3.81E-03	n.a.
	Metabochip	rs8036238	0.034	4.97E-03		rs76414746	0.007	5.33E-03		rs182185151	0.214	3.08E-03	
ABCA8	GWAS	rs4148008	0.452	8.90E-01	0.01/0.89	rs4148008	0.439	2.01E-01	0.01/1.00	rs4148008	0.321	1.31E-01	0.12/0.74
	Metabochip	rs116585097	0.014	4.92E-03		rs117047167	0.009	5.17E-03		rs4968962	0.235	1.99E-02	
HNF4A	GWAS	rs1800961	0.008	1.23E-01	0.02/1.00	rs1800961	0.013	9.89E-01	0.02/0.94	rs1800961	0.045	2.02E-02	same
	Metabochip	rs6093978	0.284	3.35E-02		rs2273618	0.346	2.68E-01		rs1800961 (T139I)	0.045	2.02E-02	

[a] LD (r^2/D') estimates were calculated from the genotype data of the PAGE African American samples, whose LD patterns may vary from any LD sources in 1000 Genomes Project

[b] LD (r^2/D') estimates were from the 1000 Genomes Project ANS samples, unless otherwise indicated

[c] LD (r^2/D') estimates were from the 1000 Genomes Project EUR samples

[d] Results of association with reported GWAS index SNPs were based on current study samples

[e] LD (r^2/D') estimates were calculated from the genotype data of the CLHNS samples, due to the SNPs are not in 1000 Genomes Project

n.a. the pairwise LD estimates are not applicable

Table S3C. Lead SNP at LDL-C loci within each ancestry group and their relative significance compared to reported GWAS index SNPs

LDL-C		African American				East Asian				European			
Locus		**Index SNP**	**MAF**	***P***	**LD (r^2/D')[a]**	**Index SNP**	**MAF**	***P***	**LD (r^2/D')[b]**	**Index SNP**	**MAF**	***P***	**LD (r^2/D')[c]**
APOE	GWAS	rs4420638	0.182	2.50E-01	0.00/0.01	rs4420638	n.a.	n.a.	0.01/1.00	rs4420638	0.227	2.03E-17	0.01/0.89
	Metabochip	rs7412 (R176C)	0.110	6.67E-75		rs7412 (R176C)	0.086	1.10E-64		rs7412 (R176C)	0.056	5.44E-76	
LDLR	GWAS	rs6511720	0.131	5.38E-14	0.63/0.95	rs6511720	0.019	3.18E-02	0.11/0.94	rs6511720	0.104	5.32E-22	0.58/1.00
	Metabochip	rs73015011	0.180	5.70E-16		rs11668477	0.142	2.37E-04		rs112898275	0.104	8.91E-23	
SORT1	GWAS	rs629301	0.350	9.00E-12	0.62/1.00	rs629301	0.038	3.08E-01	0.03/0.28	rs629301	0.228	1.82E-21	0.90/1.00
	Metabochip	rs12740374	0.247	1.45E-18		rs676961	0.047	6.88E-03		rs602633	0.230	2.08E-22	
PCSK9	GWAS	rs2479409	0.261	1.19E-01	0.02/0.93	rs2479409	0.432	1.36E-02	0.03/1.00	rs2479409	0.310	1.55E-04	0.00/0.21
	Metabochip	rs28362286 (C679X)	0.009	4.14E-22		rs2495488	0.068	7.19E-04		rs11591147 (R46L)	0.040	2.76E-30	
APOB	GWAS	rs1367117 (T98I)	0.115	3.78E-03	0.15/0.92	rs1367117 (T98I)	0.127	2.90E-02	0.72/0.92	rs1367117 (T98I)	0.284	5.72E-14	0.82/0.92
	Metabochip	rs568938	0.426	1.14E-08		rs2115838	0.135	7.60E-04		rs934198	0.298	3.71E-17	
ABO	GWAS	rs635634	0.107	1.10E-03	0.00/1.00	rs635634	0.166	1.28E-04	0.74/0.93	rs635634	0.197	2.53E-05	0.44/0.79
	Metabochip	rs9411508	0.011	7.21E-05		rs9411476	0.162	1.08E-08		rs9411378	0.301	4.06E-06	
ABCG8	GWAS	rs4299376	0.182	1.42E-02	0.16/0.67	rs4299376	0.004	4.77E-01	0.00/1.00	rs4299376	0.211	1.28E-05	0.05/1.00
	Metabochip	rs72796798	0.071	2.24E-06		rs4148217 (K400R)	0.002	4.17E-02		rs76866386	0.081	1.58E-07	
HMGCR	GWAS	rs12916	0.247	5.40E-01	0.00/0.04	rs12916	0.465	2.03E-01	0.31/0.92	rs12916	0.445	4.00E-08	same
	Metabochip	rs6453134	0.302	3.19E-03		rs253392	0.287	1.62E-03		rs12916 (3'UTR)	0.445	4.00E-08	
HPR	GWAS	rs2000999	0.072	6.88E-01	0.01/0.49	rs2000999	0.264	7.68E-01	0.61/0.90 [e]	rs2000999	0.190	3.71E-06	1.00/1.00
	Metabochip	rs1862752	0.285	1.79E-07		rs182238023	0.328	2.19E-04		rs34042070	0.190	1.59E-06	
TRIB1	GWAS	rs2954029	0.350	7.39E-01	0.05/0.87	rs2954029	0.447	6.04E-02	0.31/0.93	rs2954029	0.466	1.77E-04	0.04/0.60
	Metabochip	rs2980888	0.107	1.34E-04		rs10216723	0.439	1.45E-02		rs4419828	0.267	1.11E-04	
TIMD4	GWAS	rs6882076	0.374	4.41E-01	0.15/0.98	rs6882076	0.350	4.42E-02	0.11/1.00	rs6882076	0.325	1.64E-04	n.a.
	Metabochip	rs10075548	0.199	3.85E-03		rs72052548	0.069	2.78E-03		rs2862716 (M158T)	0.333	1.14E-04	
TOP1	GWAS	rs2902940	0.489	4.58E-01	0.00/0.06	rs2902940	0.290	6.12E-01	0.01/0.73 [e]	rs2902940	0.764	1.35E-01	0.00/0.62
	Metabochip	rs6065326	0.094	1.02E-02		rs78886984	0.060	1.58E-02		rs1883511	0.022	1.50E-05	
LDLRAP1	GWAS	rs12027135	0.460	4.78E-02	0.02/0.96	rs12027135	0.146	2.24E-01	0.63/0.90	rs12027135	0.460	8.80E-02	0.76/0.96
	Metabochip	rs3093590	0.020	1.89E-04		rs35447638	0.220	2.36E-03		rs706845	0.352	1.77E-02	
CILP2	GWAS	rs10401969	0.268	1.38E-01	0.00/1.00	rs10401969	0.049	1.53E-01	0.00/1.00	rs10401969	0.068	1.17E-03	0.64/0.80
	Metabochip	rs78712672	0.015	6.51E-03		rs149298732	0.045	1.70E-02		rs58489806	0.083	2.78E-04	
SLC22A1	GWAS	rs1564348	0.110	5.80E-01	0.00/0.32	rs1564348	0.002	9.52E-01	n.a.	rs1564348	0.145	3.24E-02	0.00/0.05
	Metabochip	rs9456502	0.187	3.97E-04		rs9457839	0.121	2.00E-03		rs3798179	0.365	5.71E-03	
MAFB	GWAS	rs2902940	0.489	4.58E-01	0.02/1.00	rs2902940	0.248	3.57E-01	0.01/1.00	rs2902940	0.236	1.35E-01	0.00/0.05
	Metabochip	rs76897158	0.018	1.68E-02		rs77016819	0.017	2.75E-03		rs2865162	0.109	6.01E-02	
ST3GAL4	GWAS	rs11220462	0.046	8.61E-01	0.00/0.21	rs11220462	0.355	6.17E-01	n.a.	rs11220462	n.a.	n.a.	0.01/1.00
	Metabochip	rs7937122	0.381	4.54E-03		rs12281220	0.003	4.73E-02		rs117603193	0.037	1.22E-02	
MYLIP	GWAS	rs3757354	0.335	1.77E-01	0.01/0.80	rs3757354	0.359	3.24E-01	0.40/1.00	rs3757354	0.260	7.72E-01	0.50/1.00
	Metabochip	rs116738939	0.020	9.04E-03		rs1011616	0.419	2.83E-02		rs72833416	0.072	1.64E-01	

[a] LD (r^2/D') estimates were calculated from the genotype data of the PAGE African American samples, whose LD patterns may vary from any LD sources in 1000 Genomes Project

[b] LD (r^2/D') estimates were from the 1000 Genomes Project ASN samples, unless otherwise indicated

[c] LD (r^2/D') estimates were from the 1000 Genomes Project EUR samples, unless otherwise indicated

[d] Results of association with reported GWAS index SNPs were based on current study samples

[e] LD (r^2/D') estimates were calculated from the genotype data of the CLHNS samples, due to the SNPs are not in 1000 Genomes Project

n.a. the pairwise LD estimates are not applicable
