# Supplementary material for: Trans-Ethnic Fine-Mapping of Lipid Loci Identifies Population-Specific Signals and Allelic Heterogeneity That Increases the Trait Variance Explained
Source: PLoS Genet. 2013 Mar 21;9(3):e1003379. doi: 10.1371/journal.pgen.1003379 (PMC3605054; doi:10.1371/journal.pgen.1003379)
Supplement: Table S4 — SNPs with the strongest association at TG (A), HDL-C (B) and LDL-C (C) loci in combined trans-ethnic meta-analysis and their associations within ancestry groups. (PDF) [file pgen.1003379.s010.pdf]

Table S4A. SNPs with the strongest association at TG loci in combined trans-ethnic meta-analysis and their associations within ancestry groups

	Trans-ethnic meta-analysis										African American				East Asian				European			
Locus	**SNP**	**A1/A2**	**EAF**	**beta**	**SE**	***P***	**Direction**	**HetI^2**	**HetP**	**Total N**	**EAF**	**beta**	**SE**	***P***	**EAF**	**beta**	**SE**	***P***	**EAF**	**beta**	**SE**	***P***
APOA5	rs662799 (5'UTR)	A/G	0.807	**-0.122**	0.006	**2.5E-91**	---+-------	82.4	1.5E-08	26232	0.880	**-0.045**	0.013	**3.4E-04**	0.722	**-0.141**	0.008	**6.1E-65**	0.921	**-0.151**	0.012	**9.3E-36**
GCKR	rs1260326 (L446P)	T/C	0.368	**0.063**	0.005	**1.6E-42**	+++++++++++	0	0.72	26226	0.149	**0.065**	0.012	**2.2E-08**	0.484	**0.056**	0.008	**1.5E-13**	0.350	**0.069**	0.007	**4.4E-24**
LPL	rs13702 (3'UTR)	T/C	0.673	**0.054**	0.005	**6.4E-29**	+++++++++++	1.9	0.42	26234	0.492	**0.044**	0.008	**6.8E-08**	0.813	**0.055**	0.010	**2.1E-08**	0.741	**0.060**	0.007	**4.2E-16**
TRIB1	rs2980888	T/C	0.248	**0.048**	0.005	**7.7E-21**	+++++++++++	58.2	7.8E-03	26209	0.109	**0.061**	0.013	**5.3E-06**	0.277	**0.035**	0.008	**3.4E-05**	0.268	**0.054**	0.007	**2.4E-13**
APOC1	rs12721054 (3'UTR)	A/G	0.881	**0.113**	0.013	**3.6E-19**	+++++??????	0	0.71	6544	0.881	**0.113**	0.013	**3.6E-19**	n.a.	n.a.	n.a.	n.a.	n.a.	n.a.	n.a.	n.a.
MLXIPL	rs6968170	A/G	0.202	**-0.045**	0.006	**7.5E-16**	-----------	37.6	0.10	26194	0.191	**-0.027**	0.011	**1.1E-02**	0.089	**-0.085**	0.013	**2.6E-10**	0.245	**-0.042**	0.008	**3.4E-08**
ANGPTL3	rs10889333	A/G	0.284	**-0.038**	0.005	**8.4E-16**	---+-------	40.4	7.9E-02	26188	0.348	**-0.027**	0.009	**1.7E-03**	0.211	**-0.034**	0.009	**2.3E-04**	0.281	**-0.049**	0.007	**1.4E-11**
APOB	rs676210 (P2739L)	A/G	0.399	**-0.031**	0.005	**6.3E-10**	-----+----+	68.9	3.7E-04	26232	0.157	**-0.052**	0.011	**4.0E-06**	0.722	**0.002**	0.009	**7.9E-01**	0.260	**-0.047**	0.007	**2.1E-10**
LIPC	rs10468017	T/C	0.254	**0.023**	0.005	**5.5E-06**	+-+----+++-	52.9	2.0E-02	26173	0.161	**0.032**	0.011	**4.0E-03**	0.182	**-0.002**	0.010	**8.0E-01**	0.328	**0.033**	0.007	**3.0E-06**
CETP	rs11076175	A/G	0.813	**-0.024**	0.006	**2.3E-05**	---+-------	0	0.82	26081	0.752	**-0.012**	0.010	**2.0E-01**	0.875	**-0.022**	0.011	**5.1E-02**	0.825	**-0.033**	0.009	**1.0E-04**
NAT2	rs4921911	T/C	0.346	**-0.018**	0.005	**4.3E-05**	-----------	0	0.85	26234	0.325	**-0.010**	0.009	**2.5E-01**	0.424	**-0.026**	0.008	**7.4E-04**	0.292	**-0.018**	0.007	**1.5E-02**
KLHL8	rs11097129	A/G	0.777	**0.020**	0.006	**5.0E-04**	+++++++++++	0	0.99	26228	0.943	**0.013**	0.018	**4.8E-01**	0.779	**0.014**	0.009	**1.4E-01**	0.746	**0.024**	0.008	**1.3E-03**
PINX1	rs10088387	T/G	0.973	**-0.089**	0.026	**6.2E-04**	-+---??????	30	0.22	6551	0.973	**-0.089**	0.026	**6.2E-04**	n.a.	n.a.	n.a.	n.a.	n.a.	n.a.	n.a.	n.a.
TYW1B	rs114204448	T/C	0.020	**0.106**	0.032	**9.0E-04**	++++???????	19.4	0.29	5405	0.020	**0.106**	0.032	**9.0E-04**	n.a.	n.a.	n.a.	n.a.	n.a.	n.a.	n.a.	n.a.
chr12	rs10735872	T/C	0.087	**0.050**	0.016	**1.8E-03**	+++-???????	33.9	0.21	5316	0.087	**0.050**	0.016	**1.8E-03**	n.a.	n.a.	n.a.	n.a.	n.a.	n.a.	n.a.	n.a.
MAP3K1	rs3936510	T/G	0.170	**0.018**	0.006	**2.7E-03**	--+++++++++	40.4	7.9E-02	26233	0.234	**0.008**	0.010	**4.2E-01**	0.113	**0.013**	0.012	**3.0E-01**	0.143	**0.030**	0.009	**1.4E-03**
CAPN3	rs117264272	A/G	0.995	**-0.196**	0.068	**3.9E-03**	----?-?????	0	0.77	7114	0.995	**-0.179**	0.070	**1.0E-02**	0.991	**-0.475**	0.286	**9.7E-02**	n.a.	n.a.	n.a.	n.a.
KLF12	rs73526970	A/G	0.117	**0.027**	0.013	**3.8E-02**	+-+++?+??-?	0	0.76	15617	0.117	**0.027**	0.013	**3.6E-02**	n.a.	n.a.	n.a.	n.a.	n.a.	n.a.	n.a.	n.a.

A1/A2: effect allele/non-effect allele

EAF: effect allele (A1) frequency

HetI^2: I^2 statistic which measures heterogeneity on scale of 0-100%

HetP: *P* values for heterogeneity across the 11 cohorts (exhibited in Table S1) included in meta-analysis

Table S4B. SNPs with the strongest association at HDL-C loci in combined trans-ethnic meta-analysis and their associations within ancestry groups

	Trans-ethnic meta-analysis										African American				East Asian				European			
Locus	**SNP**	**A1/A2**	**EAF**	**beta**	**SE**	***P***	**Direction**	**HetI^2**	**HetP**	**Total N**	**EAF**	**beta**	**SE**	***P***	**EAF**	**beta**	**SE**	***P***	**EAF**	**beta**	**SE**	***P***
CETP	rs247616	T/C	0.241	**0.088**	0.004	**7.0E-121**	+++++++++++	50.2	0.028	25375	0.258	**0.110**	0.008	**3.0E-42**	0.167	**0.071**	0.007	**1.9E-26**	0.284	**0.090**	0.006	**6.2E-59**
LIPC	rs1077834 (5'UTR)	T/C	0.621	**-0.041**	0.003	**2.5E-33**	-----------	4.5	0.40	25378	0.481	**-0.034**	0.007	**2.2E-06**	0.598	**-0.037**	0.005	**1.8E-13**	0.751	**-0.050**	0.006	**1.9E-17**
LPL	rs10096633	T/C	0.233	**0.044**	0.005	**2.8E-21**	+++++++++++	14.2	0.31	25383	0.421	**0.051**	0.007	**1.0E-12**	0.091	**0.038**	0.009	**1.6E-05**	0.101	**0.040**	0.008	**2.9E-06**
ABCA1	rs1883025	T/C	0.266	**-0.031**	0.004	**4.3E-17**	-+---------	0	0.63	25383	0.336	**-0.018**	0.008	**1.8E-02**	0.271	**-0.037**	0.006	**3.7E-11**	0.209	**-0.032**	0.006	**4.5E-07**
APOA5	rs662799 (5'UTR)	A/G	0.790	**0.033**	0.004	**1.8E-14**	+++-+++++++	34.2	0.13	25381	0.880	**0.025**	0.011	**2.4E-02**	0.722	**0.036**	0.006	**8.0E-11**	0.919	**0.033**	0.009	**4.7E-04**
LCAT	rs3785100	T/C	0.843	**-0.032**	0.005	**9.0E-12**	-----+-----	0	0.55	25348	0.783	**-0.043**	0.009	**6.5E-07**	0.904	**-0.028**	0.009	**1.7E-03**	0.845	**-0.027**	0.007	**1.6E-04**
APOB	rs1042034 (S4338N)	T/C	0.552	**-0.024**	0.004	**1.1E-10**	-++--------	26.3	0.19	25382	0.842	**-0.026**	0.010	**7.5E-03**	0.282	**-0.016**	0.006	**3.6E-03**	0.742	**-0.032**	0.006	**4.9E-08**
LIPG	rs1943973	A/G	0.833	**0.029**	0.005	**2.7E-10**	+++++++++++	0	0.75	25383	0.969	**0.026**	0.021	**2.1E-01**	0.822	**0.031**	0.007	**1.9E-06**	0.831	**0.027**	0.007	**6.9E-05**
PPP1R3B	rs6601299	T/C	0.136	**-0.034**	0.006	**8.8E-10**	---------+-	30.6	0.16	25376	0.121	**-0.059**	0.011	**8.0E-08**	0.052	**-0.016**	0.015	**2.9E-01**	0.160	**-0.028**	0.007	**1.1E-04**
SCARB1	rs921919	A/G	0.466	**-0.017**	0.003	**4.0E-07**	+++-----+--	27.9	0.18	25383	0.129	**-0.002**	0.011	**8.7E-01**	0.416	**-0.018**	0.005	**3.7E-04**	0.590	**-0.020**	0.005	**9.7E-05**
ARFGAP2	rs326222	T/C	0.570	**0.018**	0.004	**4.6E-07**	+++++-+++-+	32.7	0.14	25380	0.603	**0.021**	0.007	**4.0E-03**	0.745	**0.007**	0.006	**2.4E-01**	0.396	**0.025**	0.006	**3.6E-06**
GALNT2	rs10127775	A/T	0.600	**-0.018**	0.004	**3.5E-06**	--+++?----+	69.1	6.2E-04	23664	0.853	**-0.016**	0.011	**1.4E-01**	0.782	**-0.019**	0.007	**6.2E-03**	0.443	**-0.018**	0.005	**5.4E-04**
PLTP	rs6130969	T/G	0.074	**-0.038**	0.008	**6.0E-06**	------?----	0	0.76	19261	0.088	**-0.045**	0.013	**3.6E-04**	0.099	**-0.021**	0.016	**1.9E-01**	0.028	**-0.044**	0.016	**5.5E-03**
LILRA3	rs103294	T/C	0.247	**0.021**	0.005	**8.4E-06**	++++++?++++	0	0.77	19261	0.092	**0.024**	0.012	**5.2E-02**	0.213	**0.018**	0.011	**1.2E-01**	0.288	**0.021**	0.006	**2.1E-04**
MMAB	rs3815575	T/C	0.314	**0.016**	0.004	**1.5E-05**	++++++++-++	21.4	0.24	25372	0.540	**0.012**	0.007	**9.0E-02**	0.257	**0.020**	0.006	**6.4E-04**	0.205	**0.014**	0.006	**2.6E-02**
FADS3	rs102275	T/C	0.467	**0.013**	0.003	**1.2E-04**	+++++++++++	0	0.63	25381	0.349	**0.018**	0.008	**1.8E-02**	0.386	**0.013**	0.006	**2.4E-02**	0.590	**0.011**	0.005	**3.1E-02**
COBLL1	rs12692737	A/C	0.381	**0.015**	0.004	**1.6E-04**	++-++++++++	0	0.77	25227	0.636	**0.010**	0.007	**1.8E-01**	0.069	**0.008**	0.010	**4.2E-01**	0.340	**0.020**	0.005	**2.3E-04**
TTC39B	rs2185938	A/G	0.903	**0.020**	0.006	**9.8E-04**	++-++-++-++	23.8	0.22	25380	0.882	**0.018**	0.011	**1.0E-01**	0.971	**0.004**	0.015	**7.9E-01**	0.894	**0.026**	0.008	**1.7E-03**
STARD3	rs12150079	A/G	0.256	**0.013**	0.004	**9.9E-04**	++-+-++-+++	34.1	0.13	25371	0.084	**0.019**	0.013	**1.5E-01**	0.183	**0.017**	0.007	**7.9E-03**	0.337	**0.009**	0.005	**9.2E-02**
LACTB	rs185973484	T/C	0.291	**0.022**	0.007	**1.3E-03**	+??????++++	0	0.87	13450	0.233	**0.023**	0.016	**1.5E-01**	n.a.	n.a.	n.a.	n.a.	0.303	**0.022**	0.008	**4.2E-03**
ABCA8	rs116585097	A/G	0.014	**-0.086**	0.031	**4.9E-03**	-+---??????	0	0.75	6742	0.014	**-0.086**	0.031	**4.9E-03**	n.a.	n.a.	n.a.	n.a.	n.a.	n.a.	n.a.	n.a.
HNF4A	rs1800961 (T139I)	T/C	0.036	**-0.024**	0.010	**2.1E-02**	----?-+----	0	0.62	24235	0.008	**-0.072**	0.046	**1.2E-01**	0.013	**0.000**	0.022	**9.9E-01**	0.045	**-0.029**	0.012	**2.0E-02**

A1/A2: effect allele/non-effect allele

EAF: effect allele (A1) frequency

HetI^2: I^2 statistic which measures heterogeneity on scale of 0-100%

HetP: *P* values for heterogeneity across the 11 cohorts (exhibited in Table S1) included in meta-analysis

Table S4C. SNPs with the strongest association at LDL-C loci in combined trans-ethnic meta-analysis and their associations within ancestry groups

	Trans-ethnic meta-analysis										African American				East Asian				European			
Locus	**SNP**	**A1/A2**	**MAF**	**beta**	**SE**	***P***	**Direction**	**HetI^2**	**HetP**	**Total N**	**MAF**	**beta**	**SE**	***P***	**MAF**	**beta**	**SE**	***P***	**MAF**	**beta**	**SE**	***P***
APOE	rs7412 (R176C)	T/C	0.083	**-0.476**	0.015	**5.9E-209**	-----------	69.7	2.7E-04	24010	0.110	**-0.536**	0.026	**6.7E-75**	0.086	**-0.411**	0.024	**1.1E-64**	0.056	**-0.505**	0.027	**5.4E-76**
LDLR	rs73015011	T/C	0.872	**0.195**	0.015	**7.4E-38**	+++++++++++	0	0.98	24145	0.820	**0.194**	0.024	**5.7E-16**	0.987	**0.140**	0.060	**2.0E-02**	0.896	**0.202**	0.021	**1.5E-22**
SORT1	rs12740374	T/G	0.208	**-0.144**	0.011	**7.6E-37**	-----------	62.6	2.9E-03	24162	0.247	**-0.188**	0.021	**1.4E-18**	0.060	**-0.060**	0.029	**4.1E-02**	0.228	**-0.144**	0.015	**9.9E-22**
PCSK9	rs11591147 (R46L)	T/G	0.039	**-0.390**	0.033	**4.6E-32**	--+-???----	0	0.78	15329	0.003	**-0.595**	0.196	**2.3E-03**	n.a.	n.a.	n.a.	n.a.	0.040	**-0.384**	0.034	**2.8E-30**
APOB	rs7575840	T/G	0.230	**0.093**	0.010	**4.2E-19**	+++++++++++	18.4	0.27	24143	0.150	**0.065**	0.026	**1.2E-02**	0.136	**0.063**	0.020	**1.6E-03**	0.298	**0.115**	0.014	**7.0E-17**
ABO	rs2519093	T/C	0.181	**0.080**	0.011	**2.2E-13**	+++++++++++	0	0.91	24139	0.105	**0.103**	0.030	**6.6E-04**	0.187	**0.088**	0.018	**5.3E-07**	0.196	**0.068**	0.016	**1.7E-05**
ABCG8	rs76866386	T/C	0.924	**0.116**	0.018	**4.0E-11**	+++++++++++	0	0.56	24162	0.909	**0.133**	0.032	**3.7E-05**	0.976	**0.052**	0.048	**2.8E-01**	0.919	**0.122**	0.023	**1.6E-07**
HMGCR	rs7722186	A/G	0.522	**0.046**	0.008	**4.0E-08**	+++++++++++	0	0.77	24162	0.608	**0.051**	0.019	**7.6E-03**	0.571	**0.032**	0.014	**2.0E-02**	0.443	**0.056**	0.013	**1.1E-05**
HPR	rs72626182	T/G	0.206	**0.052**	0.011	**2.0E-06**	++++-++++++	0	0.48	24153	0.059	**0.015**	0.040	**7.0E-01**	0.255	**0.042**	0.016	**8.1E-03**	0.177	**0.070**	0.017	**2.4E-05**
TRIB1	rs4870941	C/G	0.228	**0.047**	0.010	**4.8E-06**	+-++-++++++	13.3	0.32	24159	0.107	**0.059**	0.030	**4.9E-02**	0.185	**0.043**	0.018	**1.8E-02**	0.280	**0.048**	0.014	**6.4E-04**
TIMD4	rs9715911	A/G	0.640	**0.040**	0.009	**5.7E-06**	++++-++++++	0	0.91	24162	0.461	**0.024**	0.019	**1.9E-01**	0.704	**0.039**	0.015	**9.8E-03**	0.681	**0.049**	0.013	**2.6E-04**
TOP1	rs1883511	A/C	0.980	**0.187**	0.041	**6.5E-06**	-+++++-++++	34.3	0.12	24162	0.992	**0.145**	0.107	**1.8E-01**	n.a.	n.a.	n.a.	n.a.	0.978	**0.195**	0.045	**1.5E-05**
LDLRAP1	rs35447638	A/G	0.423	**0.033**	0.009	**1.8E-04**	+-++++++-++	0	0.95	24160	0.408	**0.034**	0.019	**6.6E-02**	0.220	**0.051**	0.017	**2.4E-03**	0.545	**0.023**	0.013	**7.4E-02**
CILP2	rs17216525	T/C	0.071	**-0.055**	0.017	**8.9E-04**	-+--+-----+	50.8	0.026	24162	0.037	**0.053**	0.049	**2.8E-01**	0.081	**-0.058**	0.025	**1.9E-02**	0.070	**-0.081**	0.025	**1.3E-03**
SLC22A1	rs9457838	A/G	0.094	**-0.102**	0.032	**1.5E-03**	---+-??-?+?	9.5	0.36	13556	0.094	**-0.098**	0.032	**2.3E-03**	n.a.	n.a.	n.a.	n.a.	0.0002	**-0.018**	0.011	**9.1E-02**
chr20	rs77016819	T/G	0.017	**-0.158**	0.053	**2.8E-03**	?????--????	0	0.76	7693	n.a.	**n.a.**	n.a.	n.a.	0.017	**-0.158**	0.053	**2.8E-03**	n.a.	n.a.	n.a.	n.a.
ST3GAL4	rs114540788	T/C	0.100	**0.081**	0.031	**8.2E-03**	+++++??-+??	0	0.47	13862	0.100	**0.081**	0.031	**8.7E-03**	n.a.	n.a.	n.a.	n.a.	n.a.	n.a.	n.a.	n.a.
MYLIP	rs116738939	T/C	0.020	**-0.174**	0.067	**9.0E-03**	-+---??????	0	0.48	6436	0.020	**-0.174**	0.067	**9.0E-03**	n.a.	n.a.	n.a.	n.a.	n.a.	n.a.	n.a.	n.a.

A1/A2: effect allele/non-effect allele

EAF: effect allele (A1) frequency

HetI^2: I^2 statistic which measures heterogeneity on scale of 0-100%

HetP: *P* values for heterogeneity across the 11 cohorts (exhibited in Table S1) included in meta-analysis
