# Supplementary material for: Trans-Ethnic Fine-Mapping of Lipid Loci Identifies Population-Specific Signals and Allelic Heterogeneity That Increases the Trait Variance Explained
Source: PLoS Genet. 2013 Mar 21;9(3):e1003379. doi: 10.1371/journal.pgen.1003379 (PMC3605054; doi:10.1371/journal.pgen.1003379)
Supplement: Table S5 — LDL-C association with haplotypes consisting of the third (rs1038026) and the fourth (rs157588) signals at TOMM40-APOE-APOC4 cluster. (PDF) [file pgen.1003379.s011.pdf]

Table S5. LDL-C association with haplotypes consisting of the third (rs1038026) and the fourth (rs157588) signals at *TOMM40-APOE-APOC4* cluster

rs1038026	rs157588	hap.freq	beta	SE	*P* .glm
A	A	0.187	reference	reference	reference
G	A	0.634	-0.294	0.028	9.4E-26
A	G	0.170	-0.229	0.036	1.5E-10
G	G	0.010	-0.312	0.117	7.5E-03

The ‘haplo.glm' function implemented in the ‘haplo.stats' R package was used to calculate the coefficient β and *P*-value for each haplotype compared with the reference haplotype, which was set as the A-A (trait increasing-increasing) haplotype. The same covariates used for genotype analysis were applied in haplotype analysis. Haplotype analysis was performed in 5,593 unrelated African Americans from the PAGE consortium
