# Supplementary material for: Trans-Ethnic Fine-Mapping of Lipid Loci Identifies Population-Specific Signals and Allelic Heterogeneity That Increases the Trait Variance Explained
Source: PLoS Genet. 2013 Mar 21;9(3):e1003379. doi: 10.1371/journal.pgen.1003379 (PMC3605054; doi:10.1371/journal.pgen.1003379)
Supplement: Text S1 — Study description. (DOCX) [file pgen.1003379.s012.docx]

**Text S1. Study Description**

**I. African Americans**

***The Population Architecture Using Genomics and Epidemiology (PAGE) program.*** The PAGE program consists of study sites and a Coordinating Center [1]. The four study sites comprise large, diverse population-based studies: the Epidemiologic Architecture for Genes Linked to Environment (EAGLE), which accesses three National Health and Nutrition Examination Surveys; the Multiethnic Cohort (MEC); the Women's Health Initiative (WHI); and Causal Variants Across Life Course (CALiCo), a collection of cardiovascular cohorts including the Atherosclerosis Risk in Communities Study (ARIC). This project was limited to African American participants from ARIC [2], MEC [3] and WHI [4].

***The Hypertension Genetic Epidemiology Network (HyperGEN) study.*** HyperGEN is a multicenter family-based study to research the genetic causes of hypertension and related conditions [5]. HyperGEN recruited African American and Caucasian participants at five field centers, with recruitment based largely on ongoing population-based studies. Study participants were recruited as one of three main types of subjects: 1) as part of a hypertensive sibship with at least two siblings diagnosed with hypertension; 2) random subjects, who were age-matched with hypertensive sibs; or 3) unmedicated adult offspring of one of the hypertensive siblings. Subjects were brought into the clinic for a one day exam, and data were collected from questionnaires, a physical exam, and blood and urine samples. The study involves: University of Utah (Network Coordinating Center, Field Center, and Molecular Genetics Lab); University of Alabama at Birmingham (Field Center and Echo Coordinating and Analysis Center); Medical College of Wisconsin (Echo Genotyping Lab); Boston University (Field Center); University of Minnesota (Field Center and Biochemistry Lab); University of North Carolina (Field Center); Washington University (Data Coordinating Center); Weil Cornell Medical College (Echo Reading Center); National Heart, Lung, & Blood Institute. This study obtained informed consent from participants and approval from the appropriate institutional review boards.

**II. East Asians**

***The Cebu Longitudinal Health and Nutrition Survey (CLHNS).*** CLHNS is an on-going community-based study that began in 1983. The baseline survey randomly recruited 3,327 mother-child pairs from 17 urban and 16 rural areas from the Metropolitan Cebu area, the Philippines [6]. Overnight fasting blood samples for biomarkers and DNA were obtained at the 2005 survey. In this study, a total of 1,716 offspring samples aged 21-23 years were included in analysis.

***The Taiwan-Metabochip Study for Cardiovascular Disease (TAICHI) study.*** The TAICHI study was formed through a collaborative effort between investigators based in the U.S. and Taiwan, with the main aim to identify genetic determinants of atherosclerosis and diabetes related traits in East Asians and to fine map validated loci identified in other race/ethnic groups.

The main U.S academic sites participating in the TAICHI consortium include Stanford University School of Medicine in Stanford, California; Hudson-Alpha Biotechnology Institute in Huntsville, Alabama; and Cedars-Sinai Medical Center in Los Angeles, California. The main academic sites in Taiwan include National Health Research Institutes (NHRI); National Taiwan University Hospital (NTUH); Taipei and Taichung Veteran’s General Hospitals (VGH) and Tri-Service General Hospital (TSGH).

To help accomplish the consortium’s principal aims, a relatively large, well-phenotyped East Asian sample set consisting of ~13,500 Han Chinese subjects living in Taiwan representing several cohorts was amalgamated. A variety of relevant qualitative and quantitative traits are available in either a subset or in all cohorts. Seven cohorts comprise the current TAICHI bio-resource. Each cohort is described in more detail below.

HALST (Healthy Aging Longitudinal Study in Taiwan): a population-based epidemiologic study of older adults living in all major geographic regions of Taiwan, established by NHRI. Over 5000 subjects have been recruited over a four-year period from seven recruitment sites across the country. HALST has already ascertained or measured many clinical and biochemical phenotypes and is serving as one the main “control” cohorts for most of the qualitative phenotypes.

SAPPHIRe (Stanford-Asian Pacific Program in Hypertension and Insulin Resistance): a family-based study established in 1995 with an initial goal of identifying major genetic loci underlying hypertension and insulin resistance through linkage in East Asian populations. SAPPHIRe was also one of four networks participating the NHLBI’s Family Blood Pressure Program (FBPP). At the outset, SAPPHIRe involved recruitment sites in the San Francisco Bay Area, Hawaii, and Taiwan. Many metabolic variables were examined in baseline and regular follow-up visits by a programmatic collaboration between the NHLBI’s FBPP and the National Health Research Institutes in Taiwan. Like HALST, SAPPHIRe will also predominantly serve as a “control” cohort for most qualitative traits in TAICHI.

TCAGEN (Taiwan Coronary Artery Disease GENetic) study: an ongoing cohort study that has been enrolling patients undergoing coronary angiography or percutaneous intervention at the National Taiwan University Hospital (NTUH) in the setting of either stable angina pectoris or prior myocardial infarction. Participants are not only from the north of Taiwan where the main NTU medical school/hospital is located, but also from Yulin branch of NTUH, located in south/central Taiwan. Fasting blood samples were collected before cardiac catheterization while 10 ml of peripheral blood was collected in the catheter lab specifically for buffy coat isolation and DNA extraction.

TACT (TAiwan Coronary and Transcatheter intervention) cohort study: this study enrolled patients with angina pectoris and objective documentation of myocardial ischemia who underwent diagnostic coronary angiography and/or revascularization any time after October 2000 at the National Taiwan University Hospital (NTUH). This cohort is similar to TCAGEN but was collected independently. Participants provided clinically relevant information including use of cardiovascular related medication through a standardized questionnaire. Clinically relevant information is also available through a comprehensive electronic medical records database that includes information on drug use and surgical interventions. Fasting blood samples were collected before cardiac catheterization.

Taiwan DRAGON (Taiwan Diabetes and RelAted Genetic COmplicatioN) study: a cohort study with Type 2 diabetes at the Veteran’s General Hospital in Taichung, Taiwan (Taichung VGH). Participants include individuals with either newly diagnoses or established diabetes who visit the diabetes outpatient clinic on a regular basis. Subjects with hyperglycemia who do not meet criteria for Type 2 diabetes defined by IDF are not included. Individuals participate in a health examination program at Taichung VGH are also interviewed. Specialized tests include an oral glucose tolerance tests (OGTT) in subjects without an established diagnosis of diabetes.

TCAD (Taichung CAD study): the study includes patients with a variety of cardiovascular diseases receiving care at the Taichung Veterans General Hospital.  Specifically, individuals who were hospitalized for diagnostic and interventional coronary angiography examinations and treatment are included in TAICHI. Also included in TAICHI are subjects with a history of MI or revascularization of any type. This Taichung VGH’s CAD cohort is similar to but substantially larger than TCAGEN and TAC as it represents ~70% of the TAICHI CHD cases.

TUDR (Taiwan USA Diabetes Retinopathy): a cohort that enrolled subjects with Type 2 diabetes receiving care at Taichung Veteran’s General Hospital, a small number of subjects were included from TSGH. All TUDR subjects underwent a complete fundoscopic examination to carefully document the presence and extent of retinopathy. To date, a total of 2,222 unrelated type 2 diabetes subjects with and without retinopathy were ascertained and have undergone the Metabochip genotyping. In addition to DNA and buffy coats, fasting blood for future measurement of serum/plasma biomarkers has also been banked. A variety of additional clinical related phenotypes are available. All 2,222 overlap with the Taiwan Dragon Study.

Of the 13,500 subjects included in this sample set, buffy coat or DNA aliquots for ~11,000 were transferred to Cedars Sinai and HudsonAlpha after acquiring appropriate IRB and Taiwan Department of Health permissions for this collaboration. This transfer was followed by careful DNA extraction and/or plating. Samples were then genotyped with the Metabochip at HudsonAlpha.

**III. Europeans**

***Finland-United States Investigation of NIDDM Genetics (FUSION) Study.*** The FUSION study has been described in detail previously [7,8]. The FUSION stage 2 includes subjects chosen from the following studies:

Dehko 2D (D2D) 2004: a population-based study to screen individuals regarding T2D risk and to prevent T2D development.

Finrisk 1987: an early round of the 5-yearly Finrisk national population-based health surveys [9].

Finrisk 2002: a population-based survey of non-communicable diseases in >13,000 individuals aged 25-74 years living in 80 communities of Finland.

Action LADA: a study of latent autoimmune diabetes in adults (LADA). Action LADA investigators screened individuals aged 30-69 years with recently-diagnosed diabetes. They identified 373 T2D cases who agreed to participate in FUSION.

Health 2000: a population-based study of people aged ≥30 years from throughout Finland.

Savitaipale Diabetes Study: a study of diabetes in the town of Savitaipale in eastern Finland.

***The Dehko 2D 2007 (FIN-D2D2007) study.*** The purpose of the study is to gather information about prevalence of diabetes and cardiovascular diseases and of the risk factors associated with these within the Finnish population. The survey assists in the evaluation of the effects of the national type 2 diabetes prevention plan. The sample of the study consists of 4,500 people randomly selected from the Finnish population register. The subjects are between the ages of 45 and 74 years old and live in one of the three hospital districts chosen for the study: South Ostrobothnia, Central Finland and Pirkanmaa [10].

***The Finnish Diabetes Prevention Study (DPS).*** DPS is a prospective randomized controlled trial aimed at preventing the progression from IGT to diabetes. The original DPS was initiated in 1993. A total of 522 middle-aged, overweight subjects with IGT at baseline were randomized into either a lifestyle intervention or a standard-care control group. They were followed for occurrence of diabetes until the year 2000, when the first interim analysis of the data was carried out as originally planned. At this point the randomized trial was prematurely terminated due to markedly lower diabetes incidence rate in the lifestyle intervention group as compared to the control group. Since the termination of the randomized phase of the DPS, the original cohorts are no longer offered different treatments. However, all participants are monitored with yearly visits for long-term development of type 2 diabetes and complications [11].

***The Dose-Responses to Exercise Training (DR's EXTRA) study.*** The DR's EXTRA Study is a 4-year randomized controlled trial on the health effects of aerobic and resistance exercise training and a low-saturated fat, high-unsaturated-fat and high-fiber diet in a population sample of middle-aged and older men and women. The target population was a representative sample of 3,000 individuals (1,500 men and 1,500 women) who lived in the city of Kuopio in Finland and who were 55-74 years of age in 2002, when they were randomly selected from the national population register. Of these individuals, 2,062 were willing to participate and 1,479 (72%) participated in the baseline examinations in 2005-2006. Finally, 1,410 individuals were randomly allocated into one of the 6 study groups, each of which included about 235 persons [12].

***The METabolic Syndrome In Men Study (METSIM).*** The METSIM study aims to investigate the metabolic syndrome, type 2 diabetes, cardiovascular disease, and cardiovascular risk factors. It is an ongoing study of men aged 50 to 70 years, randomly selected from the population registry of the town of Kuopio, in Eastern Finland [13].

***The Nord-Trøndelag Health Study (HUNT 2).*** The HUNT study is a large population-based health study in Nord-Trøndelag county, central Norway (population 127,000). HUNT 2 was carried out in 1995-1997; data were collected through questionnaires, clinical exam, and blood and urine samples. 74,000 individuals (71%) participated [14].

***TROMSO*.** The Tromsø study is a prospective population-based study in the city of Tromsø in Northern Norway (population 69,000). There have been 6 study waves to date. The participation rate was >65% each time; 40,051 adults have participated at least once. For Tromsø 4 (1994), all inhabitants aged ≥25 years were invited and 27,159 (77%) participated [15].

**References**

1. Matise TC, Ambite JL, Buyske S, Carlson CS, Cole SA, et al. (2011) The Next PAGE in understanding complex traits: design for the analysis of Population Architecture Using Genetics and Epidemiology (PAGE) Study. Am J Epidemiol 174: 849-859.
2. The ARIC investigators. (1989) The Atherosclerosis Risk in Communities (ARIC) Study: design and objectives. Am J Epidemiol 129: 687-702.
3. Kolonel LN, Altshuler D, Henderson BE (2004) The multiethnic cohort study: exploring genes, lifestyle and cancer risk. Nat Rev Cancer 4: 519-527.
4. The Women's Health Initiative Study Group. (1998) Design of the Women's Health Initiative clinical trial and observational study. Control Clin Trials 19: 61-109.
5. Williams RR, Rao DC, Ellison RC, Arnett DK, Heiss G, et al. (2000) NHLBI family blood pressure program: methodology and recruitment in the HyperGEN network. Hypertension genetic epidemiology network. Ann Epidemiol 10: 389-400.
6. Adair LS, Popkin BM, Akin JS, Guilkey DK, Gultiano S, et al. (2011) Cohort profile: the Cebu longitudinal health and nutrition survey. Int J Epidemiol 40: 619-625.
7. Valle T, Tuomilehto J, Bergman RN, Ghosh S, Hauser ER, et al. (1998) Mapping genes for NIDDM. Design of the Finland-United States Investigation of NIDDM Genetics (FUSION) Study. Diabetes Care 21: 949-958.
8. Scott LJ, Mohlke KL, Bonnycastle LL, Willer CJ, Li Y, et al. (2007) A genome-wide association study of type 2 diabetes in Finns detects multiple susceptibility variants. Science 316: 1341-1345.
9. Tuomilehto J, Korhonen HJ, Kartovaara L, Salomaa V, Stengard JH, et al. (1991) Prevalence of diabetes mellitus and impaired glucose tolerance in the middle-aged population of three areas in Finland. Int J Epidemiol 20: 1010-1017.
10. Kotronen A, Yki-Jarvinen H, Mannisto S, Saarikoski L, Korpi-Hyovalti E, et al. (2010) Non-alcoholic and alcoholic fatty liver disease - two diseases of affluence associated with the metabolic syndrome and type 2 diabetes: the FIN-D2D survey. BMC Public Health 10: 237.
11. Tuomilehto J, Lindstrom J, Eriksson JG, Valle TT, Hamalainen H, et al. (2001) Prevention of type 2 diabetes mellitus by changes in lifestyle among subjects with impaired glucose tolerance. N Engl J Med 344: 1343-1350.
12. Kouki R, Schwab U, Lakka TA, Hassinen M, Savonen K, et al. (2012) Diet, fitness and metabolic syndrome - The DR's EXTRA Study. Nutr Metab Cardiovasc Dis 22: 553-560.
13. Stancakova A, Javorsky M, Kuulasmaa T, Haffner SM, Kuusisto J, et al. (2009) Changes in insulin sensitivity and insulin release in relation to glycemia and glucose tolerance in 6,414 Finnish men. Diabetes 58: 1212-1221.
14. Midthjell K, Kruger O, Holmen J, Tverdal A, Claudi T, et al. (1999) Rapid changes in the prevalence of obesity and known diabetes in an adult Norwegian population. The Nord-Trondelag Health Surveys: 1984-1986 and 1995-1997. Diabetes Care 22: 1813-1820.
15. Jacobsen BK, Eggen AE, Mathiesen EB, Wilsgaard T, Njolstad I (2011) Cohort profile: The Tromso Study. Int J Epidemiol. In press. Electronically published 2011 Apr 21.
